# Anatomical Network Analyses of Pre‐ and Post‐Metamorphic Cranial Systems Support the Adaptive Decoupling Hypothesis in Anura

**DOI:** 10.1002/jmor.70185

**Published:** 2026-10-29

**Authors:** Lorenz Guschker, Maximilian A. Stüwe, Hendrik Müller, Benjamin Naumann

**Affiliations:** ^1^ Institut für Biowissenschaften, Allgemeine und Spezielle Zoologie Universität Rostock Rostock Germany; ^2^ Zoologische Sammlung, Zentralmagazin Naturwissenschaftlicher Sammlungen Martin‐Luther‐Universität Halle‐Wittenberg Halle (Saale) Germany; ^3^ Institut für Biowissenschaften, Fachdidaktik Biologie Universität Rostock Rostock Germany

**Keywords:** complex life cycle, modularity, *Smilisca phaeota*, tadpole, topology

## Abstract

Complex life cycles characterized by distinct life phases connected by a metamorphosis raise the question whether traits of these distinct life phases can evolve independently or remain evolutionarily coupled. Because metamorphosis can include trait reorganization between life phases, the adaptive decoupling hypothesis proposes that larval and adult traits may evolve largely independent of each other. In anurans, the cranial system undergoes a major reorganization during metamorphosis, transitioning from a distinct cartilaginous larval cranium to an ossified adult cranium, yet this transformation has never been quantitatively characterized across species. We applied Anatomical Network Analysis (AnNA) to the cranial musculoskeletal systems of larval and adult anurans with different feeding and developmental modes. AnNA describes the topological organization of the cranium without the need for homologous landmarks, allowing comparisons between morphologically disparate systems. We calculated topological parameters describing integration and modularity, as well as connectivity modules (Q‐modules) within each cranial network. As part of that analysis, expanded morphological descriptions of the cranial musculoskeletal system of larval and adult *Smilisca phaeota* were made. Larval and embryonic crania are topologically highly integrated and less modular, whereas adult crania are less integrated and organized into distinct modules. As a result, larval and adult life phases occupy distinct regions within a topological morphospace, whereby larvae and embryos of endotrophic anurans, despite lacking a free‐living larval stage, cluster consistently within the larval topological space. Together, these findings support the adaptive decoupling hypothesis for the cranial system across anuran life phases.

## Graphical abstract

Cranial musculoskeletal descriptions of anurans can be used to describe the topology (organizational structure). The topology of pre‐metamorphic anurans is more integrated and less modular than that of post‐metamorphics. Topologies cluster consistently with similar life phases, despite different feeding or reproductive modes and thereby support the adaptive decoupling hypothesis for the cranial system across anuran life phases.

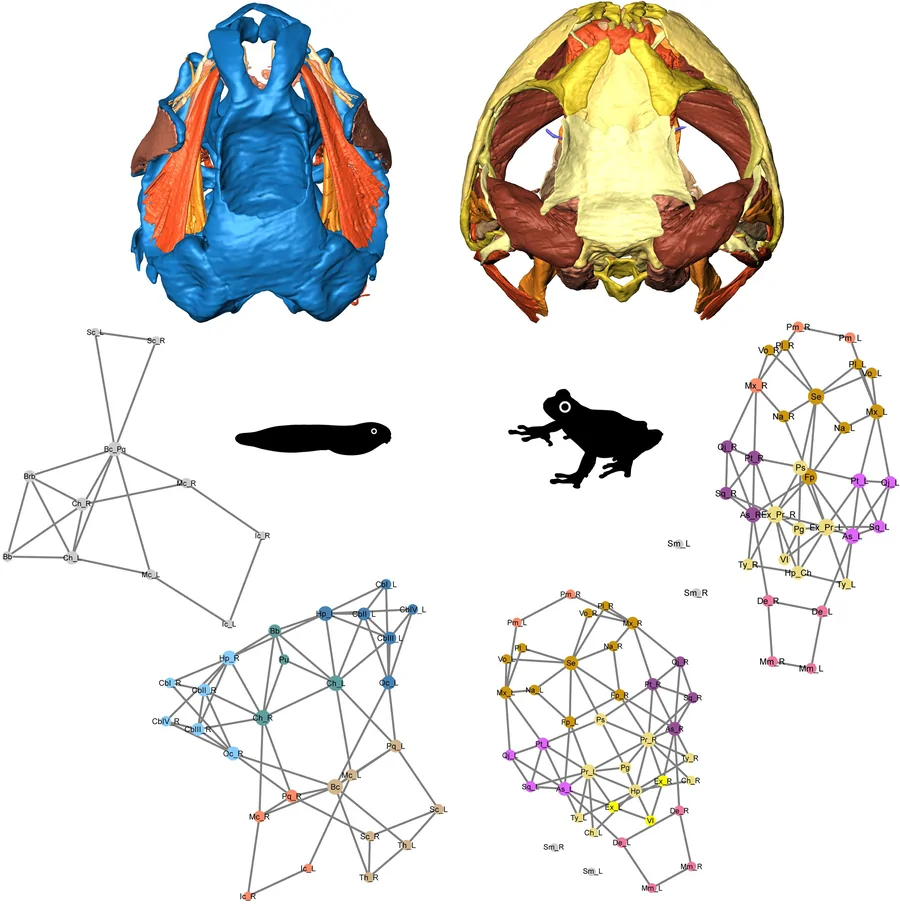

## 1 Introduction

Many animals have an indirect, or complex life cycle that consists of two or more distinct post embryonic life phases (Raff 2008). Often, these life phases differ profoundly in morphology, physiology, ecology, and behavior and are connected via a process of profound transformation called metamorphosis (Ebenman 1992; Moran 1994; Wilbur 1980). The transformation of many morphological, physiological, behavioral, and ecological characters during metamorphosis may have allowed adaptations of each distinct life phase to different selective pressures. These observations and ideas led to the formulation of the adaptive decoupling hypothesis (ADH), which states that within complex life cycles traits of different life phases can evolve largely independently (Istock 1967; Moran 1994).

Potential evidence for the ADH is found in frogs (Anura). The highly specialized larva of anurans, the tadpole, is adapted for an aquatic, omnivorous life‐style (Montaña et al. 2019). The rounded, drop shaped body and muscular tail facilitate swimming, while a completely cartilaginous cranium and a unique larval jaw, consisting of movable rostral cartilages, associated musculature and keratinous beaks enable feeding (Svensson and Haas 2005). Feeding relies on the buccal floor being raised and lowered through rotation of the ceratohyal, generating a pumping current. Keratinous beaks and labial denticles scrape algae, biofilms, and dead organic matter from the substrate, which is then carried into the pharynx by this current (Altig et al. 2007; Wassersug and Hoff 1979). Anurans show a great diversity in tadpole forms, feeding modes and habitats, which may have contributed to their evolutionary success, shown in their wide distribution range and species richness compared to the other extant amphibian groups, Gymnophiona and Caudata (Altig and Johnston 1989; Sherratt et al. 2018). Metamorphosis in many anurans includes a shift from an aquatic to a more terrestrial lifestyle. During this process, the tadpole completely transforms: limbs grow and differentiate, the tail and gills are lost and the cranium and feeding apparatus are remodeled, transitioning from specific larval mouthparts to the biting jaws of the predatory adult (Altig et al. 2007; Duellman and Trueb 1994; Wassersug 1975). In addition to this ancestral aquatic, indirect development with feeding tadpoles (exotrophy), anurans show a great diversity of reproductive and developmental modes, including many with a reduced dependency on aquatic habitats in form of water bodies (Altig and Johnston 1989; Liedtke et al. 2022). These include species that lay terrestrial eggs, from which tadpoles hatch and subsequently live an aquatic lifestyle. Others have lost the exotrophic tadpole stage entirely and instead reside within a nest, obtaining energy from the yolk (endotrophy) until metamorphosis is complete. This ecological and reproductive diversity raises the question of whether the anuran metamorphosis has allowed the more or less independent evolution of tadpole and adult traits as hypothesized by the ADH.

Evidence in favor for the ADH as an explanation for anuran diversity and species richness is not unambiguous. Traits regarding body size, behavior and the genetic basis of body size and locomotion, are correlated between life phases, as several studies indicate (Blouin 1992; Dehling and Sinsch 2019; Phung et al. 2020; Watkins 2001). The majority of anuran studies however support the idea that life phases have evolved to some degree independently, for example regarding body shape changes (in contrast to body size) and locomotor traits (Almeida‐Silva and Vera Candioti 2024; Blouin 1992; Johansson et al. 2010; Phillips 1998; Phung et al. 2020; Sherratt et al. 2017; Wollenberg Valero et al. 2017). The cranium and associated musculature are involved in many traits that exhibit profound metamorphic changes such as respiration, feeding, and, in adults, vocal communication (Duellman and Trueb 1994). However, a quantitative comparison of the cranial musculoskeletal system across both life phases is complicated by their different morphological organizations (e.g., the presence of bony components in adults vs. a mostly cartilaginous skull structure in tadpoles) and, consequently, an insufficient number of unambiguous homologous landmarks.

Anatomical network analysis (AnNA) enables the comparison of the organization (topology) between the pre‐metamorphic (tadpole/embryo) and corresponding post‐metamorphic (“adult”) cranial musculoskeletal system by quantifying the topological complexity and integration of the skeletal elements (nodes) and their connections (links), as well as the modularity of the entire cranial system (Rasskin‐Gutman and Esteve‐Altava 2014; Rawson et al. 2022). Additionally, AnNA allows for the identification of connectivity modules (Q‐modules) that can be interpreted biologically (Rasskin‐Gutman and Esteve‐Altava 2014; Werneburg et al. 2019).

In this study we test if the analysis of larval and adult cranial topology can support the ADH for anurans. We first examined the pre‐metamorphic and corresponding post‐metamorphic cranial musculoskeletal topology of the aquatic indirect developing New Granada cross‐banded tree frog, *Smilisca phaeota* (Cope 1862). We therefore provide detailed descriptions of the cranial anatomy in the tadpole and the post‐metamorphic life phase. Using these anatomical data, we performed the conventional application of AnNA, as well as a second analysis incorporating the potential influence of the developmental origin of cranial structures. We then extend the analysis to include nine different anuran species from six families (Ascaphidae, Bombinatoridae, Pipidae, Eleutherodactylidae, Alsodidae and Hylidae) to test if the results from *S. phaeota* are representative for anurans with different larval feeding and developmental modes (Cope 1862). We examine whether species with an endotrophic developmental mode (terrestrial tadpole or direct development) exhibit a closer grouping of their pre‐ and post‐metamorphic life phases compared to species with exotrophic tadpoles.

## 2 Materials and Methods

### 2.1 Specimens and Data Collection

Larval and adult *Smilisca phaeota*, provided by private breeders, were kept at the Institut für Zoologie und Evolutionsforschung (Friedrich‐Schiller‐Universität, Jena, Germany) in 2016 according to §11 of the TierSchG. Animals were euthanized using 1% tricaine methanesulfonate (MS‐222), fixed in 4% phosphate buffered formalin and stored in 70% ethanol. Staging of larval *S. phaeota* followed Gosner (1960).

Data of adult anurans and the corresponding larvae or embryos of six families (Ascaphidae, Bombinatoridae, Pipidae, Eleutherodactylidae, Alsodidae and Hylidae), which represent different feeding and developmental modes, were collected from the literature and from scans obtained from MorphoSource (Table 1) (Blackburn et al. 2024). Pre‐metamorphic topologies were derived from descriptions of tadpole crania ranging up to Gosner stage 39 (mean: Go 32). For *S. phaeota*, larval skeletal topology was supplemented with data from Ziermann‐Canabarro and Lukas (2024), while topology with muscular connections were based on descriptions from the present study. The adult topology of *Eupsophus roseus* was compared to the tadpole topology of the closely related species *E. calcaratus* and *E. emiliopugini*, for which anatomical data was available (Vera Candioti et al. 2005, 2011). The adult topology of *Eleutherodactylus coqui*, was compared to the topology of the corresponding embryo at developmental stage TS 11 (Townsend and Stewart 1985), at which embryonic musculature is fully developed, but before remodeling occurs (Hanken et al. 1997; Ziermann and Diogo 2014).

**Table 1:** Literature sources used to reconstruct the cranial musculoskeletal networks.

species	phase	literature used	3D data used
*Ascaphus truei*	adult	Altig (1969), Carroll and Holmes (1980), Johnston (2011)	morphosource: Media 000011253
*Ascaphus truei*	larva	Gradwell (1973), Haas (1997), Pusey (1943), Reiss (1997)	
*Bombina orientalis*	adult	Maglia and Púgener (1998)), Trewavas (1932)	morphosource: Media 000593900
*Bombina orientalis*	larva	Haas (1997), Haas (2001), Lukas and Olsson (2020)	
*Eleutherodactylus coqui*	adult	Hanken et al. (1992), Hanken et al. (1997), Kerney et al. (2010), Lynch (1993), Schlosser and Roth (1997), Ziermann and Diogo (2014)	Morphosource: Media 000600923
*Eleutherodactylus coqui*	embryo	Hanken et al. (1992), Hanken et al. (1997), Kerney et al. (2010), Schlosser and Roth (1997), Ziermann and Diogo (2014)	
*Eupsophus roseus*	adult		morphosource: Media 000600927, morphosource: Media 000021083
*Eupsophus calcaratus*	larva	Vera Candioti et al. (2005)	
*Eupsophus emiliopugini*	larva	Vera Candioti et al. (2011)	
*Hymenochirus boettgeri*	adult	Paterson and Goodrich (1946), Ridewood (1900), Sokol (1969)	
*Hymenochirus boettgeri*	larva	Lukas et al. (2024), Sokol (1962)	
*Smilisca phaeota*	adult	Duellman and Trueb (1966), Trueb (1968)	this study, morphosource: Media 000101187
*Smilisca phaeota*	larva	Ziermann‐Canabarro and Lukas (2024)	this study
*Xenopus laevis*	adult	Paterson (1939), Porro and Richards (2017), Smirnov (1994), Trueb and Hanken (1992), Ziermann and Diogo (2014)	
*Xenopus laevis*	larva	Candioti (2007), Lukas and Olsson (2018), Paterson (1939), Trueb and Hanken (1992), Ziermann and Diogo (2014)	

### 2.2 X‐Ray Microscopy and 3D Reconstruction

Specimens were contrasted in 2% phosphotungstic acid for several days. X‐ray microscopic imaging was performed using a Zeiss XRadia Versa 410. The scanning parameters for the adult reconstructed in this study were 60 kV, 8 W and an exposure time of 11 ms (pixel size 29.474 µm). For the tadpole scan reconstructed in this study, scanning parameters were 60 kV, 8 W and an exposure time of 3 ms (pixel size 7.3751 µm). The acquired tiff image stacks were processed using Fiji. Scans for reconstruction, as well as scans acquired from morphosource were analyzed using Amira 2021.2 (Thermo Fisher, USA). Scans of *S. phaeota* (adult: *n* = 2, tadpole: *n* = 1) were used to reconstruct the cranial skeletal elements and musculature. The terminology of adult skeletal elements and muscles follows Duellman and Trueb (1994), while the terminology of tadpole anatomy follows Haas (2003).

### 2.3 Anatomical Network Analysis

Unweighted, undirected network models were created using adjacency matrices as done previously (Esteve‐Altava et al. 2018, 2019; Plateau and Foth 2020; Rawson et al. 2022; Strong et al. 2022; Werneburg et al. 2019). Nodes represent skeletal structures and links between nodes describe contacts via sutures, joints, muscles, and ligaments. Connections of nodes were coded as “1”, while absence was coded as “0”. Adjacency matrices were compiled in Excel and analyzed in R 4.5.2 (Team 2025) and RStudio 2025.09.2 (Posit Team 2025) with the package *igraph* (Csardi and Nepusz 2006), using a modified script from Esteve‐Altava (2022), Rawson et al. (2022) and Werneburg et al. (2019) that calculates a number of mathematical parameters. These parameters describe the topology of the network and include the number of nodes (N) and links (K), density of connections (D), clustering coefficient (C), mean shortest path length (L), heterogeneity (H) and parcellation (P). Density of connections (D) describes the realized connections in the network compared to the maximum possible, while clustering coefficient (C) measures the number of triangular motifs in the network. Mean shortest path length (L) is the arithmetic mean of the shortest path length between all pairs of nodes. Together, density of connections (D), clustering coefficient (C) and mean shortest path length (L) describe the complexity of an anatomical system. Heterogeneity (H) describes the disparity of connectivity of nodes and represents anisomerism, while parcellation (P) determines whether nodes are divided into many subunits and is used to describe modularity of networks. All parameters were calculated as described previously (Rasskin‐Gutman and Esteve‐Altava 2014; Rawson et al. 2022).

For each species and life phase, we constructed two adjacency matrices, each based on a different criterion for defining nodes, while the definition of links remained the same between them. The first represents the conventional application of this method, in which fused skeletal elements are accounted for as a single node, which we will refer to as topological analysis. Additionally, alternative matrices were generated to assess the potential influence of the developmental origin of cranial structures. In this second approach, termed developmental‐topological analysis, nodes are defined according to chondrification centers, so that skeletal elements are coded as separate nodes even if they fuse during development. For example, the otic capsules and palatoquadrate merge to the neurocranium and ultimately form part of the braincase. In the topological analysis, the fused braincase is reflected as a single node, whereas in the developmental‐topology analysis, they remain separate nodes corresponding to its distinct chondrification center.

### 2.4 Q‐Module Analysis

Network modules were identified using a hierarchical clustering of the generalized topological overlap similarity matrix among nodes (GTOM) (Plateau and Foth 2020; Strong et al. 2022; Werneburg et al. 2019). GTOM expects that nodes that connect to the same other nodes are more likely to belong to the same module. This analysis results in a hierarchical grouping of nodes in a dendrogram, where proximity of nodes represents similar connections to other nodes (Figures S1.1, S1.2). Modules were detected using the optimization function modularity Q (Clauset et al. 2004), which compares the partition of the dendrogram to randomly assembled dendrograms. Q close to zero represents module partition not discernable from random networks. Modules of a network are best partitioned at Q_max_, where the dendrograms are cut, with strongly modular networks usually ranging from 0.3 to 0.7. Networks exhibiting Q_max_ values below 0.3 were considered not significantly modular and connectivity modules recovered by GTOM were accordingly not considered in subsequent interpretation and network coloring. Recovered clusters of the cut dendrograms represent Q modules. Nodes not connected to any other node were not treated as distinct modules. A jackknife procedure estimated the error of Q by treating each link as an independent observation. Each dendrogram cluster was tested with a one‐sided Wilcoxon rank‐sum test to assess whether it exhibits more in‐module connections than out‐module connections. The statistical significance of modularity is displayed at the roots of the clusters (no symbol: *p* ≥ 0.5; white: *p* < 0.05; grey: *p* < 0.01; black: *p* < 0.001) (Figures S1.1, S1.2). Following Werneburg et al. (2019), these statistical modules were interpreted in relation to the Q‐modules.

### 2.5 Principal Component Analysis

To visualize topological variation, principal component analyses (PCA) were performed of five topological parameters for both the topological and developmental‐topological analyses. The number of nodes (N) and links (K) were excluded to mitigate the effect of network size (see PCA including N and K in Supporting Information S1: Figure S1.3). The significance of topological variation between larvae/embryo and adults was tested using a PERMANOVA in the *adonis2* function of the R package *vegan* (Dixon 2003) implementing 10,000 replications (Esteve‐Altava et al. 2019; Rawson et al. 2022). Rejection of the null hypothesis, that larval and adult cranial network topology do not differ, would indicate that ontogenetic stage is a significant predictor of topological organization.

### 2.6 Topological Similarity Across Life History Stages

To evaluate whether each larval anatomical network more closely resembled its corresponding adult network or the networks of larvae of other species, we computed a pairwise Euclidean distance matrix based on the extracted network parameters. Each value in the matrix represents the dissimilarity between two individuals, enabling comparisons both within and across life history stages. For each larva, we extracted the distance to its corresponding adult (d_LA_) and the mean distance to all other larvae/embryo (d_LL_). We then calculated the difference between these values (diff = d_LA_ – d_LL_) as a measure of similarity. A negative value indicates that the larva/embryo more closely resembles its corresponding adult, whereas a positive value indicates greater similarity to other larvae. An equivalent analysis was applied to adults. For each adult, we extracted the distance to the corresponding larva/embryo (d_AL_) and the mean distance to all other adults (d_AA_), then calculated the difference (diff = d_AL_ – d_AA_). Here, a negative value indicates that the adult more closely resembles its corresponding larva than other adults, whereas a positive value shows greater similarity to other adults. This analysis allowed us to assess topological similarity within and across life history stages, providing a measure of species‐specific changes in topology across metamorphosis.

As the distribution of these differences was non‐normal and the sample size was small, we used a Wilcoxon signed‐rank test for both analyses. Each test was applied against zero using the one‐sided alternative hypothesis, that d_LA_ > d_LL_ and d_AL_ > d_AA_, respectively. In other words, we tested whether distances between pre‐ and post‐metamorphic individuals were systematically greater than distances within the same stage, which would suggest that individuals at the same life history stage are more similar to each other than to their corresponding post‐metamorphic stages.

### 2.7 Software

Images acquired from 3D reconstructions, as well as plots generated during analyses were edited and arranged using Adobe Illustrator CS6. Icons representing adult *Smilisca phaeota* and *Bombina orientalis* were obtained from PhyloPic (phylopic.org), where they were freely available in the public domain.

## 3 Results

### 3.1 Cranial Musculoskeletal Anatomy of Pre‐ and Post‐Metamorphic *Smilisca phaeota*

The cranial development of larval *S. phaeota* has been previously described in detail by Ziermann‐Canabarro and Lukas (2024), the chondrocranium at Gosner 26–27 is briefly described here (Figure 1).

**Figure 1:**
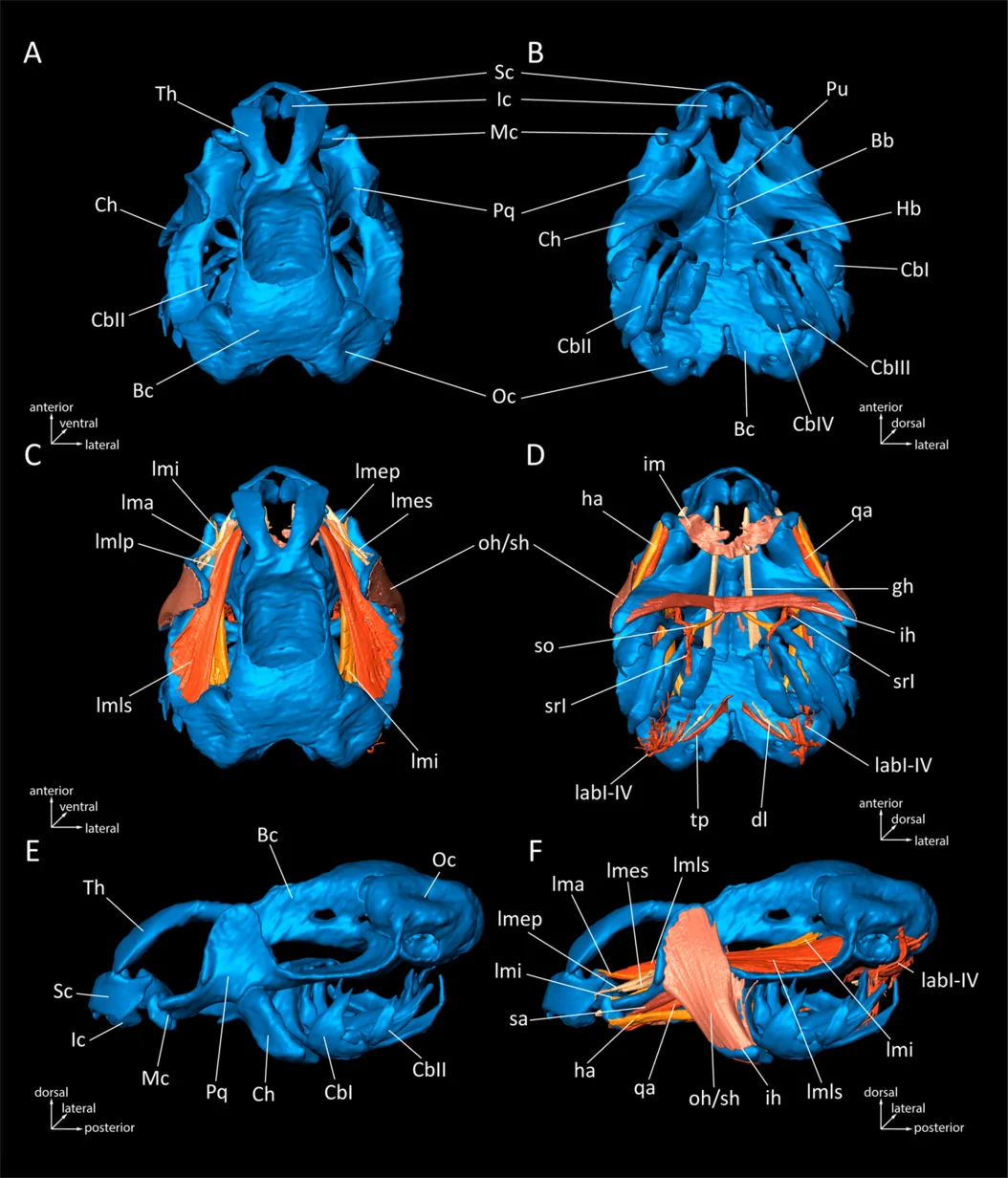
Chondrocranium and musculature of larval *Smilisca phaeota* (Gosner stage 26–27). (A and B) Chondrocranium in dorsal (A) and ventral (B) view. (C and D) Chondrocranium and cranial musculature in dorsal (C) and ventral (D) view. (E and F) Chondrocranium in lateral view without (E) and with (F) cranial musculature. Bb = Basibranchial, Bc = Braincase, CbI‐IV = Ceratobranchial I‐IV, Ch = Ceratohyal, Hb = Hypobranchial plate, Ic = Infrarostral cartilage, Mc = Meckel's cartilage, Oc = Otic capsule, Pq = Palatoquadrate, Pu = Pars reuniens, Sc = Suprarostral cartilage, Th = Trabecular horn, dl = M. dilatator laryngis, gh = M. geniohyoideus, ha = M. hyoangularis, ih = M. interhyoideus, im = M. intermandibularis, labI‐IV = Mm. levator arcuum branchialium I‐IV, lma = M. levator mandibulae articularis, lmep = M. l. m. externus profundus, lmes = M. l. m. externus superficialis, lmi = M. l. m. internus, lmlp = M. l. m. longus profundus, lmls = M. l. m. longus superficialis, oh = M. orbitohyoideus, qa = M. quadratoangularis, sa = M. suspensorioangularis, sh = M. suspensoriohyoideus, so = M. subarcualis obliquus, srI = M. subarcualis rectus I, tp = M. tympanopharyngeus.

The maximum width of the chondrocranium is in the middle of the muscular process and the subocular bar. The robust, paired suprarostral cartilages meet medially and form a U‐shaped structure with pronounced dorsal processes that articulate with the trabecular horns. The trabecular horns correspond to approximately one quarter of the total chondrocranial length and diverge from the braincase. The lateral walls of the braincase are well chondrified, leaving a dorsal opening of the chondrocranium. The ovoid otic capsules are dorsally connected by a large cartilaginous bridge that projects anteriorly to cover the posterior third of the brain. The palatoquadrate shows a high muscular process and attaches to the braincase via a broad quadratocranial commissure, an ascending process anteroventrally to the otic capsule and a short otic process. Meckel's cartilages are comma‐shaped and bear rounded retroarticular processes. The infrarostral cartilage is robust, curved and articulate medially. In the hyobranchial skeleton, the ceratohyal is long and has a curved, anteriorly directed anterior process, a short anterolateral process and a rounded lateral process. The ceratohyals are joined medially by a pars reuniens similar in length to the basibranchial. The hypobranchial plates join medially and diverge posteriorly. The four pairs of ceratobranchials are robust and characterized by numerous protrusions, with terminal commissure III representing the only clearly discernible commissure. Forty cranial muscles were identified in the *S. phaeota* tadpole, see Figure 1 and Table 2 for further details.

**Table 2:** Cranial, hyoid, and hyobranchial musculature of larval *Smilisca phaeota* (Gosner stage 26–27).

Muscle	Insertions	Comment
Intermandibularis	Ventral surface of Meckel's cartilage—median raphe	Thin muscle sheet
Interhyoideus	Posteroventral surface of the ceratohyal—median raphe	
Levator mandibulae internus	Ventral surface of ascending process of palatoquadrate and braincase—lateral part of Meckel's cartilage	Inserts via a long tendon, inserts more medial on the ascending process than lmls and lmlp
Levator mandibulae externus superficialis	Anteromedial margin of muscular process—dorsolateral part of Meckel's cartilage	Inserts lower on muscular process than lmep and lma
Levator mandibulae externus profundus	Anteromedial margin of muscular process—lateral margin of suprarostral	Inserts more ventral on muscular process to lma and dorsal to lmes, insertion shared with lmlp
Levator mandibulae articularis	Anteromedial margin of muscular process of palatoquadrate— dorsomedial edge of Meckel's cartilage	Very few muscle fibers, inserts more medial than lmls, insertion on muscular process more dorsal to lmes and lmep
Levator mandibulae longus superficialis	Posterodorsal surface of palatoquadrate—dorsomedial edge of Meckel's cartilage	Insertion shared with lmlp, inserts more lateral to lmls
Levator mandibulae longus profundus	Posterodorsal surface of palatoquadrate—lateral margin of Meckel's cartilage	Insertion shared with lmls, inserts together with lmep
Orbitohyoideus	Anterodorsal margin of muscular process—lateral edge of ceratohyal	Big muscle, covers muscular process laterally
Suspensorioangularis	Posteroventral margin of muscular process—lateroventral margin of Meckel's cartilage	Course medial to oh, inserts medial to ha
Quadratoangularis	Ventral surface of palatoquadrate at muscular process—lateroventral margin of Meckel's cartilage	Insertion posterior to sa and ha
Hyoangularis	Anterolateral surface of the ceratohyal—lateroventral margin of Meckel's cartilage	Insertion lateral to sa
Geniohyoideus	Posteroventral surface of the hypobranchial plate— posteroventral surface of the infrarostral	
Levator arcuum branchialium I	Posteroventral surface of otic capsule—posteroventral surface of ceratobranchial I	Very small muscle, only few muscle fibers
Levator arcuum branchialium II	Posteroventral surface of otic capsule—posteroventral surface of ceratobranchial II	Very small muscle, only few muscle fibers
Levator arcuum branchialium III	Posteroventral surface of otic capsule—posteroventral surface of ceratobranchial III	
Levator arcuum branchialium IV	Posteroventral surface of otic capsule—posteroventral surface of ceratobranchial IV	
Subarcualis rectus I	Posteromedial margin of ceratohyal—ventrolateral surface of hypobranchial plate	Consists of two slips
Subarcualis obliquus	Posterior margin of basibranchial— anteroventral margin of of ceratobranchial II and III	Splits in two separate slips leading to to CbII and CbIII
Tympanopharyngeus	Ventral surface of the otic capsule—lateral surface of glottis	Inserts dorsal to dl
Dilatator laryngis	Ventral surface of the otic capsule—lateral surface of glottis	Inserts ventral to tp

A brief additional description of the adult *S. phaeota* cranium is provided here (Figure 2). The head of the adult is notably wider than long, with a width‐to‐length ratio of approximately 1.3 and its greatest width occurring at the suture between the maxillary and quadratojugal. The anterior third of the maxillary extends dorsally, forming a flange twice the width of the posterior maxillary. The nasal, anterodorsal to the frontoparietal, possesses a long maxillary process extending nearly to the maxillary. The sphenethmoid is fully ossified. The frontoparietals are completely fused and bear strong supraorbital flanges. The squamosal is large, the long anterior arm extends approximately one‐third of the maxillary length. The vomers meet medially and extend anteriorly as distinct processes. The palatine contacts the sphenethmoid medially and extends laterally and posteriorly to nearly reach the pterygoid. The large pterygoid extends anteriorly to nearly half the length of the maxillary. The lower jaw is composed primarily of the angulosplenial, with the dentary positioned laterally and extending from the mentomeckelian bone to about the mid‐length of the angulosplenial. The mentomeckelian bones are clearly separated medially. The cartilaginous hyoid plate is about four times wider than long, bearing well‐ossified thyrohyals (posteromedial processes) posteriorly and thin anterolateral processes anteriorly. The hyale is thin and curves anterolaterally, then posterolaterally and dorsally toward the lateral surface of the prootic. Forty one muscles were identified in adult *S. phaeota* (Figure 2 and Table 3).

**Figure 2:**
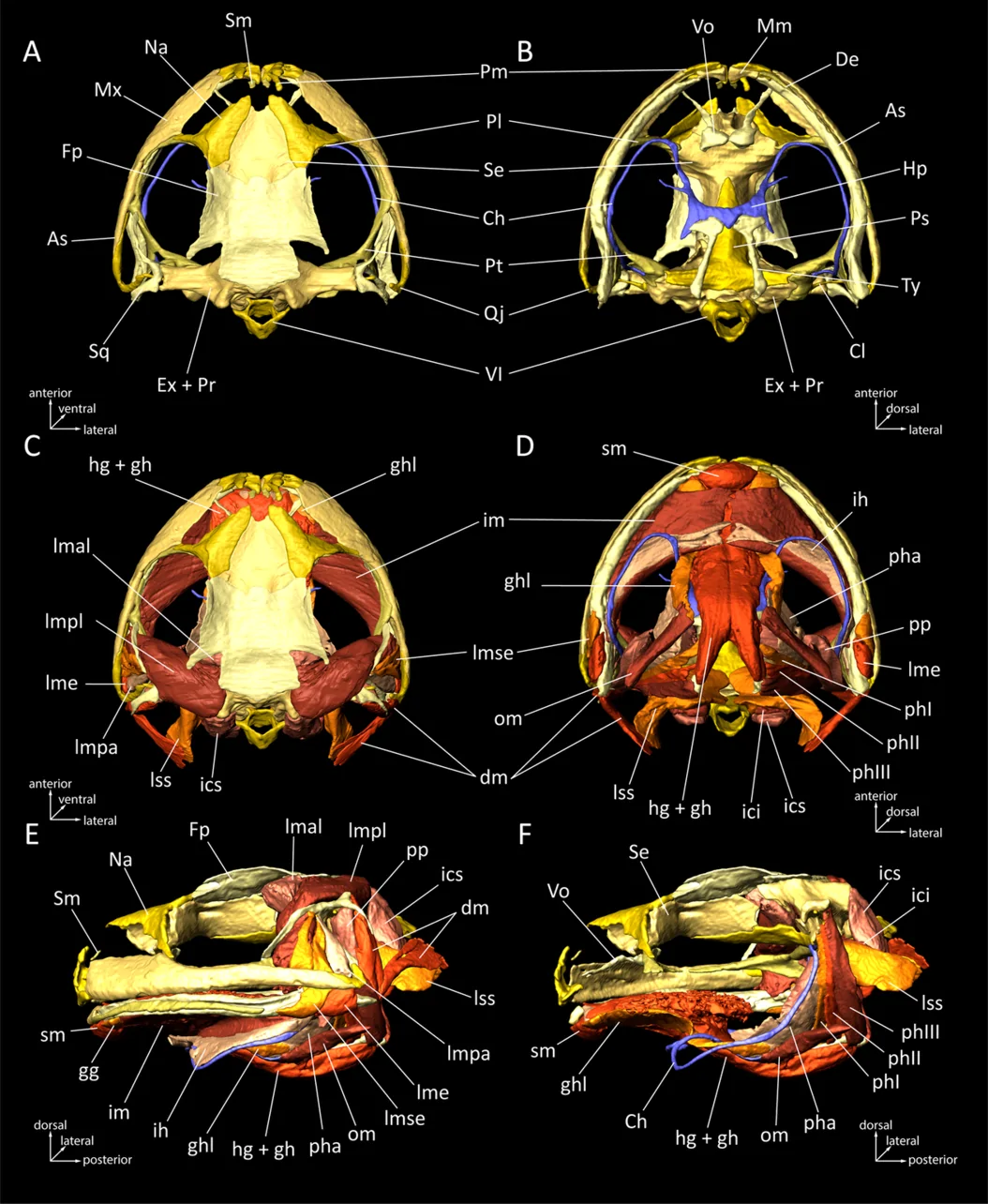
Cranium and musculature of adult *Smilisca phaeota*. (A and B) Cranium in dorsal (A) and ventral (B) view. (C and D) Cranium and cranial musculature in dorsal (C), ventral (D) and lateral (E) view. (F) Lateral view with the jaw (De, As, Mm, Mx, Pt, Sq, Pm) and associated jaw musculature (lmal, lme, lmpa, lmpl, lmpla, lmse, im, ih, pp) removed. As = Angulosplenial, Ch = Ceratohyal, Cl = Columella, De = Dentary, Ex = Exoccipital, Fp = Frontoparietal, Hp = Hyoid plate, Mm = Mentomeckelian bones, Mx = Maxillary, Na = Nasal, Pg = Pectoral girdle, Pl = Palatine, Pm = Premaxillary, Pr = Prootic, Ps = Parasphenoid, Pt = Pterygoid, Qj = Quadratojugal, Se = Sphenethmoid, Sm = Septomaxilla, Sq = Squamosal, Ty = Thyrohyal, VI = Vertebra I, Vo = Vomer, dm = M. depressor mandibulae, gg = M. genioglossus basalis, gh = M. geniohyoideus, ghl = M. geniohyoideus lateralis, hg = M. hyoglossus, ici = M. intertransversarius capitis inferior, ics = M. intertransversarius capitis superior, ih = M. interhyoideus, im = M. intermandibularis, lmal = M. levator mandibulae anterior longus, lme = M. l. m. posterior externus, lmpa = M. l. m. posterior articularis, lmpl = M. l. m. posterior longus, lmse = M. l. m. posterior subexternus, lss = M. levator scapula superior, om = M. omohyoideus, pha = M. petrohyoideus anterior, phI‐III = Mm. petrohyoideus I‐III, pp = M. protractor pectoralis, sm = M. submentalis.

**Table 3:** Cranial, hyoid, and hyobranchial musculature of adult *Smilisca phaeota*.

Muscle	Insertions	Comment
Submentalis	Medial surface of anterior Angulosplenial—medial surface of anterior Angulosplenial	
Intermandibularis	Nearly entire median surface of Angulosplenial—median raphe to meet the muscle from the other side	Fuses posteriorly with ih
Interhyoideus	Dorsomedial surface of ascending Hyale—median raphe to meet the muscle from the other side	Fuses anteriorly with im
Levator mandibulae anterior longus	Ventrolateral surface of Frontoparietal, lateral and ventrolateral surface of Prootic— dorsomedial margin of Angulosplenial	Insertion via a long tendon, insertion on As quite posterior
Levator mandibulae posterior longus	Dorsolateral surface of Exoccipitals and Prootic—dorsomedial margin of Angulosplenial	Insertion on As anterior to lmpa
Levator mandibulae posterior articularis	Anteroventral surface of Squamosal (ventral ramus)—dorsomedial margin of Angulosplenial	Insertion on ascending ventral ramus of Sq, insertion on As posterior to lmpl and anterior to lmal
Levator mandibulae posterior subexternus	Anteroventral margin of Squamosal (zygomatic ramus)—ventrolateral surface of Angulosplenial	Insertion on As more anterior than lme
Levator mandibulae posterior externus	Ventromedial surface of Quadratojugal—ventral and ventrolateral surface of Angulosplenial	Insertion on As more posterior than lmse
Levator mandibulae posterior lateralis	Anteroventral margin of Squamosal (anterior ramus)—lateral surface of Angulosplenial	Small muscle, insertion on As between Mlmse and Mlme
Depressor mandibulae	Dorsal fascia, posterior surface of Squamosal and posterolateral Prootic—posterodorsal surface of jaw joint	Two distinct parts
Geniohyoideus	Base of thyrohyal—anteromedial surface of Angulosplenial	Lies ventral to Hyoglossus, course not clearly distinguishable from hg
Geniohyoideus lateralis	Posterolateral margin of hyoid plate—anteromedial surface of Angulosplenial	
Genioglossus basalis	Anteromedial surface of Angulosplenial—medial surface of Mentomeckelian bones	
Hyoglossus	Posterior end of thyrohyal—tip of tongue	Biggest muscle in tongue
Omohyoideus	Posterolateral surface of hyoid plate—medial surface of Scapula	
Petrohyoideus anterior	Lateral surface of Prootic—broad insertion on lateral margin of hyoid plate	Sheet‐like, shared origin with phI, phII, phIII
Petrohyoideus I	Lateral surface of Prootic—lateral surface of base of thyrohyal	Shared insertion with pha, phII, phIII
Petrohyoideus II	Lateral surface of Prootic—lateral surface of thyrohyal	Shared insertion with pha, phI, phIII
Petrohyoideus III	Lateral surface of Prootic—lateral surface of distal part of thyrohyal	Shared insertion with pha, phI, phII
Protractor pectoralis	Lateral surface of Prootic—dorsal surface of Scapula	Insertion lateral to ph and medial to dm
Levator scapula superior	Posteroventral surface of Parasphenoid and Prootic—medial surface of Suprascapula	
Intertransversarius capitis inferior	Posterior surface of Prootic—ventral surface of diapophysis of Vertebra II	
Intertransversarius capitis superior	Dorsal surface of Exoccipital—dorsal surface of diapophysis of Vertebra II	

### 3.2 Metamorphosis of Cranial Topology in *Smilisca phaeota*

We used two different anatomical network approaches to study the topology of larval and adult *S. phaeota*. Networks of the first type are based solely on the observed connectivity (topological) pattern of skeletal elements (called topology networks from hereon) (Figure 3A,B). Networks of the second type include information on the developmental origin of the skeletal elements in form of distinct chondrification and ossification centers, resulting in larger networks (called developmental‐topology networks from hereon) (Figure 3C,D). Both analyses reveal comparable changes in topology across metamorphosis, though with varying degrees of intensity, as topology networks show more pronounced parameter changes than developmental‐topology networks. When comparing the two approaches directly, adult topology networks and developmental‐topology networks are highly similar to one another, whereas the corresponding tadpole networks differ more substantially between the two approaches.

**Figure 3:**
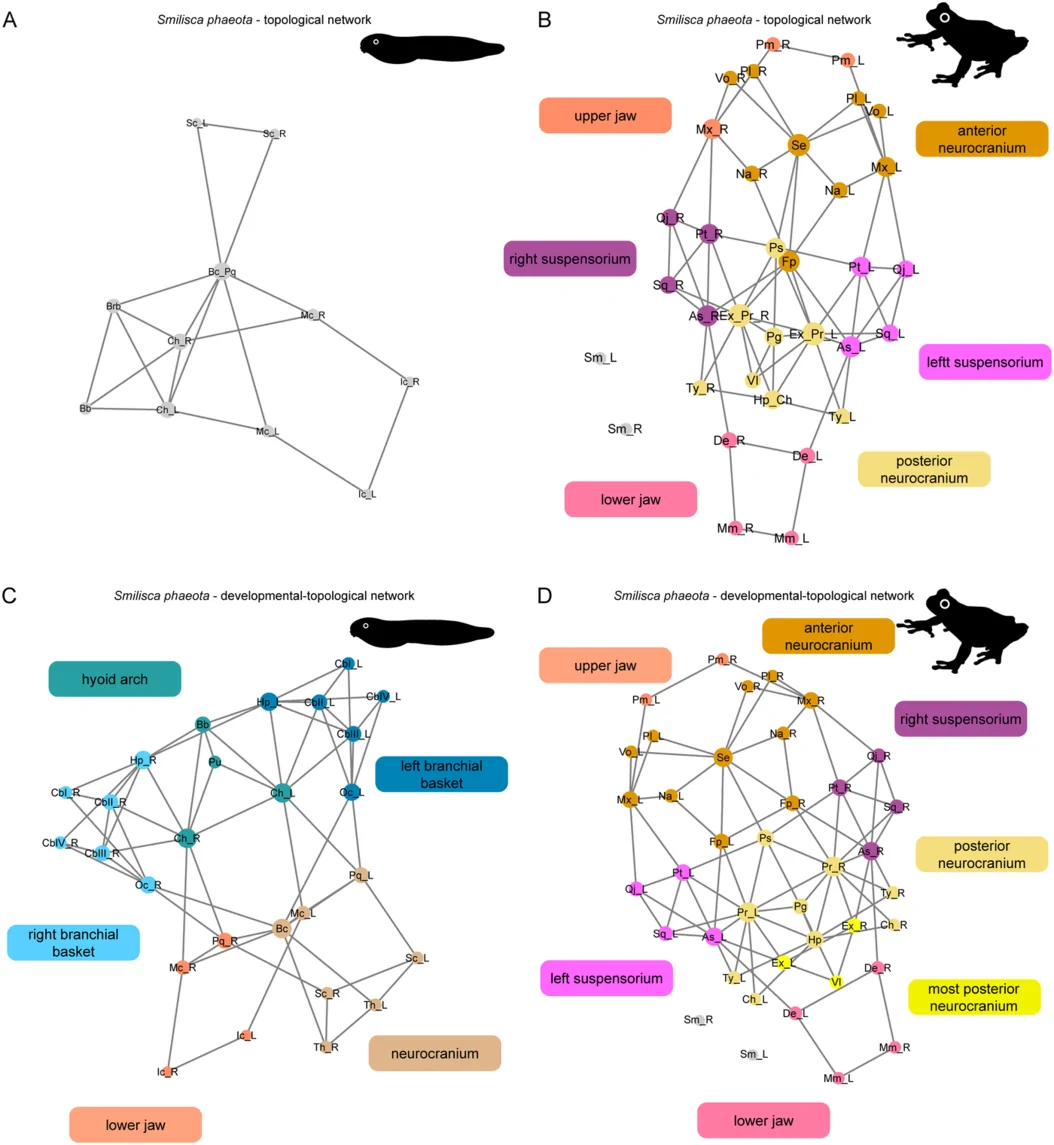
Topology networks and identified Q‐modules of (A) larval and (B) adult *Smilisca phaeota*. Developmental‐topology networks with identified Q‐modules of (C) larval and (D) adult *Smilisca phaeota*. Networks with non‐significant modularity were colored entirely in grey. Abbreviations of the skeletal elements for the networks are found in Table 5.

The number of nodes (N) and links (K) increases with metamorphosis. Density of connections (D) and clustering coefficient (C) decrease, likely connected to the addition of new skeletal elements (e.g., bones). Additionally, the mean shortest path length (L) increases. Network heterogeneity (H) is generally higher in the adult, reflecting the existence of more defined hubs, most notable is the prootic, which connects multiple regions including the neurocranium, hyoid and suspensorium, followed by the sphenethmoid and angulosplenial (Table 4).

Modularity also increases with metamorphosis, as reflected by higher parcellation (P). GTOM analysis of topological networks in tadpoles recovers no support for significant modularity (Q_max_ = 0.21, significantly modular networks show Q_max_ between 0.3 and 0.7) (Clauset et al. 2004; Werneburg et al. 2019), whereas adult topological networks exhibit six connectivity modules. In the developmental‐topological analysis, five connectivity modules were recovered in the tadpole and seven in the adult (Table 4).

**Table 4:** Network and GTOM parameters from topological and developmental‐topological analyses for all examined species. The number in brackets indicate the number of Q‐modules, when skeletal elements not integrated in the network are included.

Topological analysis											
Species	Stage	N	K	D	C	L	H	P	Q Modules	Q_max_	Q_max_ error
*Ascaphus truei*	Adult	35	49	0.082	0.413	2.823	0.825	0.856	5 (13)	0.519	0.064
*Ascaphus truei*	Larva	8	11	0.393	0.294	1.750	0.466	0.469	2	0.211	0.150
*Bombina orientalis*	Adult	33	61	0.116	0.440	2.535	0.84	0.843	5 (7)	0.379	0.064
*Bombina orientalis*	Larva	7	12	0.571	0.628	1.524	0.371	0.490	2	0.080	0.127
*Eleutherodactylus coqui*	Adult	34	75	0.134	0.452	2.550	0.627	0.682	5 (7)	0.422	0.054
*Eleutherodactylus coqui*	Embryo	6	8	0.533	0.727	1.467	0.512	0.444	3	0.008	0.179
*Eupsophus roseus*	Adult	34	73	0.130	0.378	2.571	0.724	0.682	7 (9)	0.343	0.060
*Eupsophus calcaratus*	Larva	7	12	0.571	0.628	1.524	0.371	0.490	2	0.080	0.127
*Eupsophus emiliopugini*	Larva	12	21	0.318	0.770	1.803	0.650	0.486	2	0.244	0.100
*Hymenochirus boettgeri*	Adult	33	71	0.134	0.409	2.831	0.568	0.812	8	0.414	0.060
*Hymenochirus boettgeri*	Larva	7	13	0.619	0.833	1.429	0.432	0.490	2	0.047	0.118
*Smilisca phaeota*	Adult	34	71	0.127	0.320	2.647	0.600	0.837	6 (8)	0.433	0.058
*Smilisca phaeota*	Larva	11	19	0.345	0.544	1.873	0.473	0.661	5	0.219	0.105
*Xenopus laevis*	Adult	25	57	0.190	0.520	2.071	0.664	0.810	4 (6)	0.264	0.068
*Xenopus laevis*	Larva	7	13	0.619	0.595	1.381	0.256	0.490	2	0.047	0.118

Developmental‐topological analysis											
*Ascaphus truei*	Adult	41	69	0.084	0.465	2.775	0.840	0.819	4 (12)	0.475	0.051
*Ascaphus truei*	Larva	29	77	0.190	0.439	2.298	0.367	0.744	4	0.370	0.054
*Bombina orientalis*	Adult	42	85	0.099	0.468	2.812	0.730	0.839	6 (8)	0.451	0.053
*Bombina orientalis*	Larva	27	64	0.182	0.313	2.328	0.373	0.647	5	0.385	0.061
*Eleutherodactylus coqui*	Adult	39	94	0.127	0.438	2.589	0.611	0.664	7 (9)	0.382	0.050
*Eleutherodactylus coqui*	Embryo	20	34	0.179	0.330	2.295	0.629	0.645	4	0.410	0.084
*Eupsophus roseus*	Adult	41	90	0.110	0.337	2.711	0.681	0.722	7 (9)	0.403	0.052
*Eupsophus calcaratus*	Larva	26	59	0.182	0.428	2.428	0.319	0.725	5	0.445	0.061
*Eupsophus emiliopugini*	Larva	29	62	0.153	0.312	2.532	0.414	0.737	6	0.446	0.062
*Hymenochirus boettgeri*	Adult	33	70	0.133	0.409	2.854	0.586	0.812	8	0.421	0.060
*Hymenochirus boettgeri*	Larva	16	30	0.250	0.413	2.058	0.430	0.727	4	0.302	0.093
*Smilisca phaeota*	Adult	39	82	0.111	0.324	2.743	0.627	0.832	7 (9)	0.445	0.054
*Smilisca phaeota*	Larva	27	62	0.177	0.391	2.393	0.402	0.743	5	0.449	0.060
*Xenopus laevis*	Adult	33	77	0.146	0.438	2.344	0.593	0.736	6 (8)	0.404	0.057
*Xenopus laevis*	Larva	23	54	0.213	0.460	2.154	0.366	0.745	5	0.331	0.068

### 3.3 Cranial Topology Through Anuran Metamorphosis

To assess whether the cranial changes observed in *S. phaeota* represent a general pattern, we extended the analysis to species with differing feeding and developmental modes. Two additional indirectly developing anurans with grazing tadpoles, *Ascaphus truei* (Figure 4A–D) and *Bombina orientalis* (Figure 4E–H) were included. The indirectly developing *Hymenochirus boettgeri*, which has a predatory tadpole and an aquatic adult (Figure 5A–D) and *Xenopus laevis*, with a suspension feeding tadpole and aquatic adult were also included (Figure 5E–H). Finally, endotrophic development is represented by the directly developing *Eleutherodactylus coqui* (Figure 6A–D) and indirect developing *Eupsophus* (Figure 6E–K). Across all taxa, metamorphic changes in cranial topology are largely similar, with differences between adult and tadpole topologies being more distinct in topology networks than in developmental‐topology networks (Table 4).

**Figure 4:**
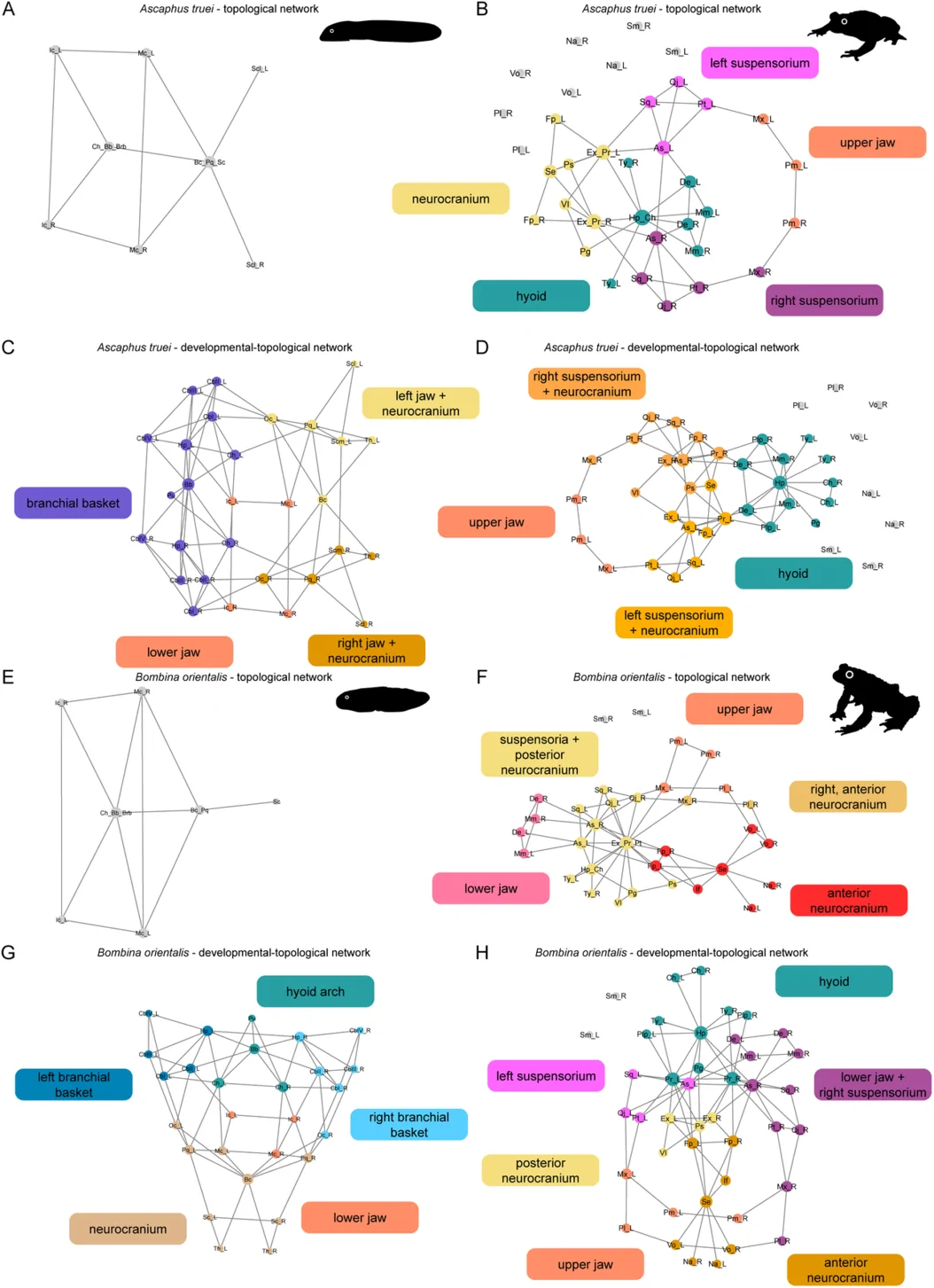
Anatomical networks and connectivity modules (Q‐modules) of *Ascaphus truei* (A–D) and *Bombina orientalis* (E–H), shown for larvae (A, C, E, G) and adults (B, D, F, H). For each species, the upper network represents topological analysis (A, B, E, F) and the lower network represents development‐topological analysis (C, D, G, H). Networks with non‐significant modularity were colored entirely in grey. Abbreviations of the skeletal elements for the networks are found in Table 5.

**Figure 5:**
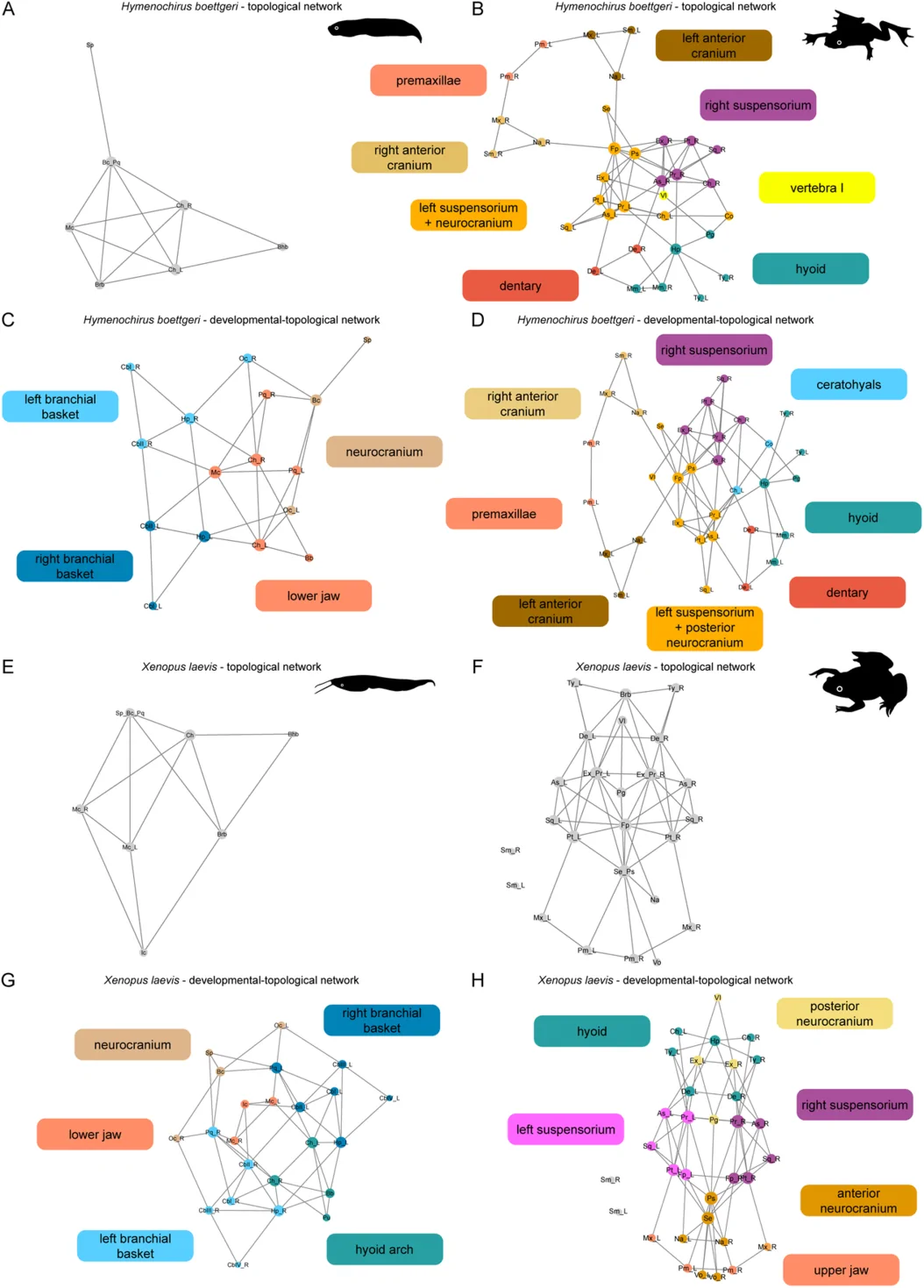
Anatomical networks and connectivity modules (Q‐modules) of *Hymenochirus boettgeri* (A–D) and *Xenopus laevis* (E–H), shown for larvae (A,C,E,G) and adults (B, D, F, H). For each species, the upper network represents topological analysis (A, B, E, F) and the lower network represents development‐topological analysis (C, D, G, H). Networks with non‐significant modularity were colored entirely in grey. Abbreviations of the skeletal elements for the networks are found in Table 5.

**Figure 6:**
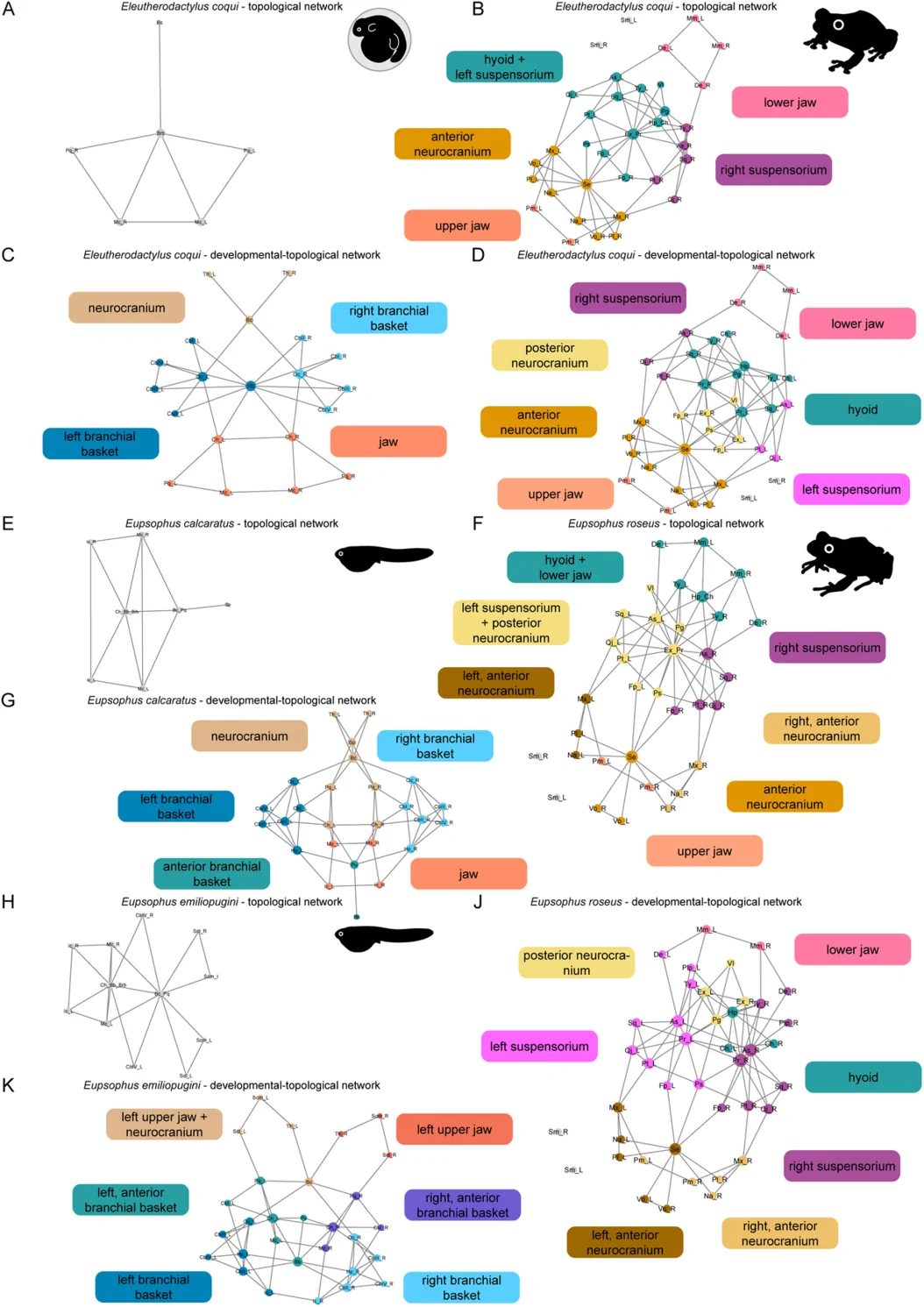
Anatomical networks and connectivity modules (Q‐modules) of embryonic (A, C) and adult *Eleutherodactylus coqui* (B, D), larval *Eupsophus calcaratus* (E, G), adult *Eupsophus roseus* (F, J) and larval *Eupsophus emiliopugini* (H, K). For each species, the upper network represents topological analysis (A, B, E, F, H) and the lower network represents development‐topological analysis (C, D, G, J, K). Networks with non‐significant modularity were colored entirely in grey. Abbreviations of the skeletal elements for the networks are found in Table 5.

**Table 5:** Abbreviations representing structures of the generated anatomical networks (Figures 3, 4, 5, 6).

general			
_L	Left side of the cranium	_R	Right side of the cranium
*Larva*		*Adult*	
Bb	Basibranchial	As	Angulosplenial
Bc	Braincase	Ch	Ceratohyal
Brb	Branchial basket	Co	Columella
CbI	Ceratobranchial I	De	Dentary
CbII	Ceratobranchial II	Ex	Exoccipital
CbIII	Ceratobranchial III	Fp	Frontoparietal
CbIV	Ceratobranchial IV	Hp	Hyoid plate
Ch	Ceratohyal	If	Interfrontal
Hp	Hypobranchial plate	Mm	Mentomeckelian bones
Ic	Infrarostral cartilage	Mx	Maxillary
Lj	Lower jaw	Na	Nasal
Mc	Meckel's cartilage	Pg	Pectoral girdle
Oc	Otic capsule	Pl	Palatine
Pq	Palatoquadrate	Plp	Posterolateral process
Pu	Pars reuniens	Pm	Premaxillary
Sc	Suprarostral cartilage	Pr	Prootic
Scl	Lateral suprarostral cartilage	Ps	Parasphenoid
Scm	Medial suprarostral cartilage	Pt	Pterygoid
Sp	Suprarostral plate	Qj	Quadratojugal
Th	Trabecular horn	Se	Sphenethmoid
		Sm	Septomaxilla
		Sq	Squamosal
		Ty	Thyrohyal
		VI	Vertebra I
		Vo	Vomer

### 3.4 Changes of Topology Networks

Adult topology networks contain more nodes (N) and links (K) than larval/embryonic ones (Table 4 and Figure 7). Tadpole/embryo networks exhibit higher density of connections (D) and higher clustering coefficient (C), whereas adults have a higher mean shortest path length (L). In adults, hubs are more pronounced, reflected by higher heterogeneity (H). Hubs were identified as the most highly connected nodes within the network. Across nearly all taxa, the prootic and angulosplenial act as hubs, while the sphenethmoid does so in several species. This pattern is consistent with hubs observed in *S. phaeota*. Adults additionally exhibit higher modularity, described by higher parcellation (P).

**Figure 7:**
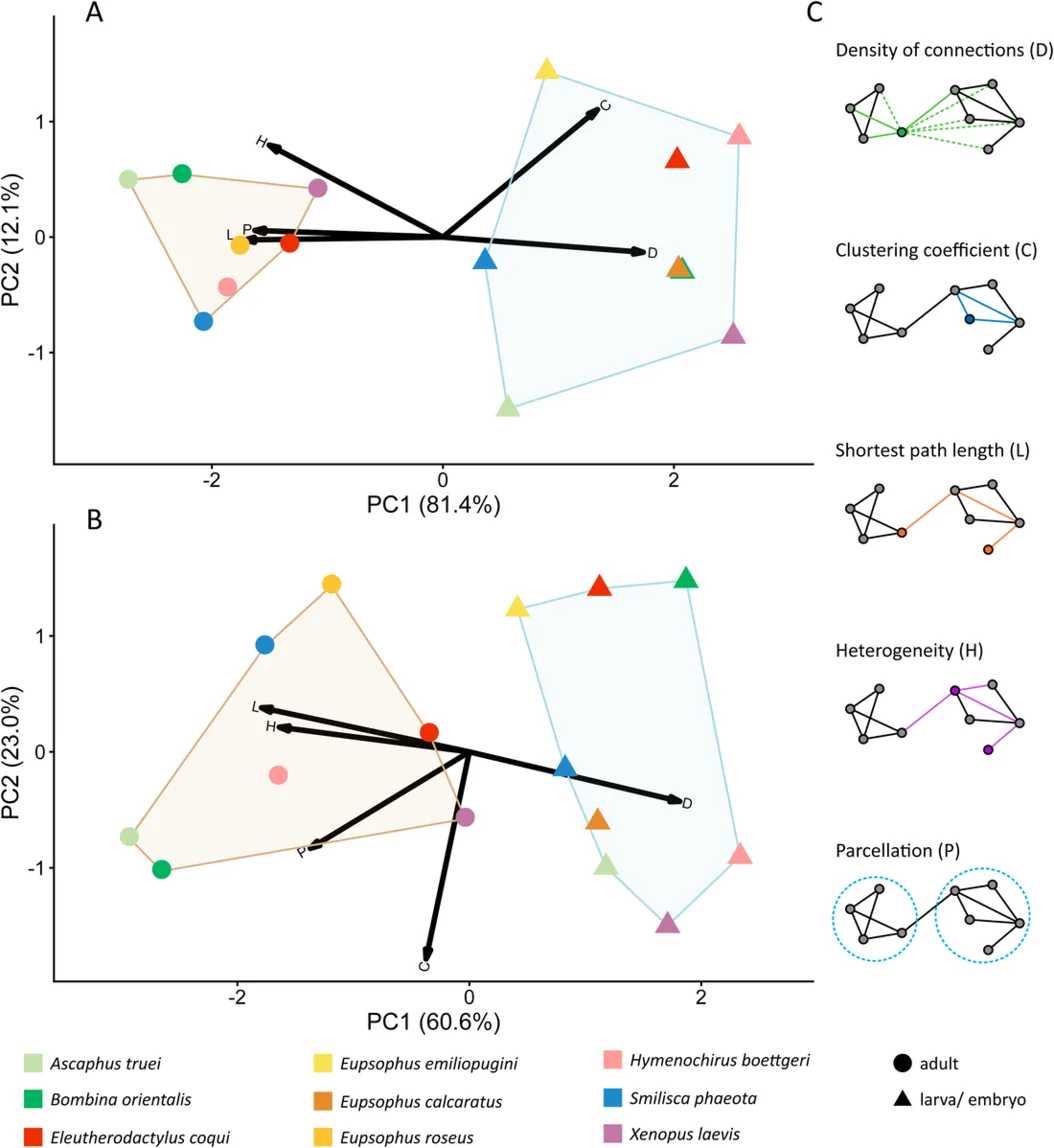
Principal Component Analysis of network parameters calculated from (A) topological and (B) developmental‐topological analyses of adult and larval/embryonic cranial musculoskeletal system. (C) Schematic illustration of the anatomical network parameters used in the analyses, modified from Esteve‐Altava et al. (2018).

GTOM analysis did not recover significant modularity in the topology networks of all tadpoles/embryos and of all adult *X. laevis*. Overall, an average of 5.7 Q‐modules was recovered for adult networks, generally corresponding to the anterior and posterior neurocranium, the paired suspensorium, hyoid and jaws (Figures 4, 5, 6). The number of recovered Q‐modules reflects those integrated in the network. The differing number of modules across species are presented in Table 4.

The PCA separated adult and larval/embryonic topologies into significantly distinct regions of the topological morphospace (PERMANOVA: *F* (1,13) = 30.749, *p* = 0.0003). These distinct differences are mainly described by PC1, which accounts for 81.4% of differences (Figure 7). Wilcoxon signed‐rank test showed that the Euclidean distances between larvae and their corresponding adults are significantly greater than zero (V = 36, *p* = 0.007), indicating that larval topology is significantly more similar to other larvae than to their corresponding adults. Similarly, adults are significantly more similar to other adults than to their larvae (V = 36, *p* = 0.007).

### 3.5 Changes of Developmental‐Topology Networks

Developmental‐topology networks reveal patterns broadly consistent with those described from topology networks, with adults showing a higher number of nodes (N) and links (K), except for *A. truei*, which shows a decrease of links with metamorphosis. Tadpoles/embryos display higher density of connections (D), while adults have a higher mean shortest path length (L). Clustering coefficient (C) does not describe changes of topology during metamorphosis, but rather species‐specific differences (Figure 7). Heterogeneity (H) increases in all but *Eleutherodactylus*. Similar to topology networks, parcellation (P) also increased with metamorphosis, describing higher modularity.

GTOM analysis recovered an average of 6.4 Q‐modules in adults compared to 4.75 in tadpoles/embryos. As in the topological analysis, these counts reflect modules that are integrated in the network. Module numbers by species and life phase are provided in Table 4. Adult Q‐modules are generally similar to the ones recovered for topological networks. Q‐modules recovered for the tadpoles largely correspond to the neurocranium, larval jaw and the branchial basket, which is mostly organized into left and right modules. Larval/embryonic module organization varies considerably among species, with no consistency in module boundaries. The ceratohyals, in particular, are associated with different modules depending on the species: the branchial basket, neurocranium or jaw module or even form a separate module altogether (Figures 4, 5, 6).

PCA of developmental‐topology networks confirms significant separation of adults and tadpoles/embryos (PERMANOVA: *F* (1,13) = 11.176, *p* = 0.0004) (Figure 7), where 60.6% of the differences are accounted by PC1. Wilcoxon signed‐rank tests of the Euclidean distances showed significant differences between the life phases (V = 36, *p* = 0.007 for larvae/embryo and V = 33, *p* = 0.021 for adults). This pattern is consistent with the PERMANOVA and highlights the similarity of topology within life phases.

## 4 Discussion

In this study, we describe the cranial musculoskeletal anatomy of larval and adult *Smilisca phaeota* and use AnNA to investigate metamorphic changes of the cranial musculoskeletal topology of anurans. This type of analysis allows the comparison of disparate anatomical systems (Esteve‐Altava et al. 2018; Rawson et al. 2022) and ontogenetic differences (Plateau and Foth 2020) by using network theory to analyze connections within an anatomical system, which provides insights into integration and modularity (Esteve‐Altava et al. 2011; Rasskin‐Gutman and Esteve‐Altava 2014; Strong et al. 2022). The calculated topological parameters allow interpretations of how structures interact within a network, providing insights into the organization of the cranial system. Comparisons of these anatomical systems between pre‐ and post‐metamorphic life phases thus allows us to investigate the adaptive decoupling hypothesis in anurans.

### 4.1 Metamorphosis in *Smilisca phaeota* Is Characterized by Cranial Topological Reorganization and Changes in Modularity

The cranial anatomy of *Smilisca phaeota* has been partially described in previous work, including larval cranial development (Ziermann‐Canabarro and Lukas 2024), adult osteology of the closely related species, *S. baudini* (Trueb 1968) and comparisons within *Smilisca* (Duellman and Trueb 1966). The present study expands on this by providing descriptions of the larval head musculature and the adult cranium, including the musculature. Building on this anatomical basis, we observed changes in the topology described by network parameters across metamorphosis of the omnivorous larval to the predatory adult cranium: density of connections (D) decreased, while mean shortest path length (L) and parcellation (P) increased (Table 4). These changes indicate a decrease in topological complexity and integration, and a strong increase in modularity (Esteve‐Altava et al. 2018). This suggests, that metamorphosis allows the anuran cranial system to reorganize specific to different life phases, reflecting the transition of habitat types and the connected change in feeding mode and respiration (Duellman and Trueb 1994). During metamorphosis, the entire cranium is remodeled, for example, the rostral cartilages disappear entirely (Ziermann‐Canabarro and Lukas 2024), the branchial basket undergoes major transformation and forms the adult hyoid plate and associated structures (Hanken and Summers 1988). Additionally, bones form, contributing new structures (nodes) and muscular insertion sites to the anatomical system. In *S. phaeota*, as well as the other examined species, these topological transformations are associated with a cranial reorganization characterized by a decrease of topological complexity and integration and an increase in modularity (Table 4).

In evolutionary developmental biology, modules are considered to consist of tightly integrated units that are only loosely connected or integrated with other units of the system (Klingenberg 2008; Kuratani 2009; Wagner et al. 2007). In network theory, modules are defined as groups of nodes (representing units) that have more links (connections) among themselves than with external nodes (Esteve‐Altava 2020; Plateau and Foth 2020; Werneburg et al. 2019). This gross overlap in defining modules based on some kind of interaction allows to interpret modules derived from AnNA within a morphological or biological context (Rasskin‐Gutman and Esteve‐Altava 2014). However, these interpretations have to be made with caution. Connectivity‐based modularity can reflect common developmental origin, as the elements that develop from the same precursor tend to stay close to each other and eventually become linked as development proceeds, as well as functional groupings of the anatomical system, such as joints or contact surfaces to distribute stresses. Modules derived from AnNA must therefore be reevaluated post hoc to identify their explanatory value within a biological context. Evidence for the biological relevance of connectivity modules comes from a morphological study of the larval cranium of *Hymenochirus*, which identified the hyobranchial apparatus as distinct from the ceratohyals (Sokol 1962) (Figure 5A–D). A modular configuration that is independently reproduced in the developmental‐topology networks presented here. AnNA of other morphological systems similarly recovered connectivity modules that correspond to previously described functional or developmental units, for example, the bipartite division of facial and cranial modules in the human skull (Esteve‐Altava et al. 2013) and the division of the vertebrate limb into several subunits (Esteve‐Altava et al. 2018; Molnar et al. 2017).

A comparison with morphometric studies further underscores this potential. A large‐scale morphometric covariance analysis of adult anuran crania in 172 species identified the suspensorium as a key module, composed of the quadratojugal, pterygoid and squamosal (Bardua et al. 2020). Using AnNA, we identified a comparable connectivity module defined by the quadratojugal, pterygoid and the angulosplenial. The squamosal was identified as part of the suspensorium module in all species except *E. coqui*. The complete composition of the suspensorium module varied across species, often including additional skeletal elements, reflecting the individual modularity recovered by AnNA.

Differences in the exact composition of the modules between the two approaches likely results from methodological differences between covariance analyses and AnNA. Covariance analysis of the crania examine the change of the skeleton between species resulting in the identification of covariation modules and are based on the form and connections between skeletal elements via direct contacts (Bardua et al. 2020). Connectivity‐based Q‐modules in contrast incorporate connections via bony articulations, ligaments and muscles, but not the forms of skeletal elements. The inclusion of muscular connections introduces additional links, especially to the lower jaw, are often not included in landmark‐based morphometry, which is apparent in the recovered functional association of the suspensorium and the jaw with AnNA. Similarly, the recovery of a jaw module using covariance analysis (Bardua et al. 2020) further demonstrates the complementary value of AnNA and other morphometric approaches in the study of modularity.

Interestingly, the topology analysis recovered highly variable modules, with the ceratohyals in particular showing inconsistent associations across species. This inconsistency might either reflect a methodological artifact that results from the low number of nodes or indicate a general lack of modularity within tadpole crania or it might instead reflect taxon‐specific differences in feeding and reproductive modes. Generally, the ceratohyal is found as a separate module or in association with the branchial basket (Figures 3, 4, 5, 6). In *H. boettgeri*, however, it is associated with the jaw (Figure 5C), which might reflect the predatory feeding mode (Sokol 1962). Similarly, in endotrophic species, the ceratohyals are closely associated with the jaw. This might reflect the developmental repatterning of the cranium that occurs with the loss of a feeding larva (Hanken et al. 1997; Kerney et al. 2010; Ziermann and Diogo 2014). Further studies on modularity in species with different feeding and developmental modes can help to test the previous results from the present study and gain further insights into the relationship between tadpole feeding mode and cranial modularity.

Our analyses show that, during metamorphosis, the number of connectivity modules generally increases from tadpole to the corresponding adult stage. However, there is considerable variation in the number of modules among the analyzed species. No increase was observed in the developmental‐topology networks of *A. truei*, in which both adult and larval forms exhibit four connectivity modules (Figure 4C,D). The low number of recovered connectivity modules may be explained by the relatively poorly ossified cranium of adult *A. truei* (Altig 1969). This results in many unlinked skeletal elements, creating for example low connectivity in the anterior neurocranium, insufficient to combine into a connectivity module (Altig 1969; Moore and Townsend Jr. 2003).

Interestingly, no significant connectivity modules in larval topology networks (without developmental information) were recovered, as the threshold of Q_max_ (0.3) is not met (Clauset et al. 2004; Werneburg et al. 2019). At least two explanations are possible. First, the apparent low modularity in tadpoles indicates that their cranial system functions and evolves as a highly integrated unit without significant topological modularity. The integrated tadpole cranium may reflect the functional coupling of the jaws and branchial basket, both of which are involved in feeding and respiration movements as well as locomotion in some tadpoles living in fast flowing streams (e.g., *A. truei*) (Wassersug and Hoff 1979). Small changes to skeletal elements can affect the entire highly integrated cranial system. In contrast, the high modularity of the adult cranium may compartmentalize morphological changes to within individual modules, without necessarily affecting the entire cranium.

Second, the lack of modularity may partly reflect methodological limitations. In networks with few nodes, community detection may fail to recover real modular organization as significant modularity values (Q_max_), potentially underestimating modularity (Esteve‐Altava 2020; Fortunato and Hric 2016). Networks showing recovered modules despite non‐significant Q_max_ values are provided in Supporting Material (Figures S1.4–S1.7). Additionally, AnNA as applied in this study does not account for the type or the number of links between nodes. Links are encoded as binary connections regardless of whether the link represents a single suture or a joint and multiple muscles. Skeletal elements with multiple connections likely experience more selective pressure than elements with a single link, which almost certainly influences the recovery of modularity. Incorporating the type and the number of connections into future AnNA therefore would improve the characterization and the study of modularity in anatomical systems.

### 4.2 AnNA Reveals a Topological Reorganization During Anuran Metamorphosis Independent of the Larval Feeding Mode

In this study, we applied two types of AnNA to characterize cranial topology. A topological analysis, consistent with widespread use of AnNA, and a developmental‐topological analysis that incorporates information on the developmental origin of structures. Differences between pre‐ and post‐metamorphic topologies are more pronounced in the topological analysis. Because many skeletal elements of the tadpole cranium fuse, their topology networks contain few nodes, whereas fusion of skeletal elements is less common in adults, resulting in similar network sizes of post‐metamorphic networks. Larger differences in network size of tadpoles/embryos may explain the greater variation in the resulting topological parameters describing these networks (Rasskin‐Gutman and Esteve‐Altava 2014). Although the two approaches differ in their detailed topological variation, both recover a clear distinction between pre‐ and post‐metamorphic cranial systems.

In the topology networks and developmental‐topology networks, each adult cranial network occupies a distinct place within the topological morphospace (Figure 7). Tadpoles show a comparable pattern, with the exception of the topology networks of *B. orientalis* and *E. calcaratus*, which overlap. Despite their phylogenetic distance (Figure S1.8) and differing feeding modes, the exotrophic grazer *B. orientalis* and the endotrophic *E. calcaratus* show an unexpected similarity. One possibility to interpret the close clustering of tadpole/embryo topologies is that high morphological disparity of tadpole crania is linked to a high integration of its single structures that may generally favor more global evolutionary changes of the cranial system. This interpretation mainly rests on the low cranial modularity as characterized by a high density of connections (D), low mean shortest path length (L), high heterogeneity (H) and high parcellation (P).

The species studied show different feeding modes. Along with more generalized tadpoles, the predatory tadpole of *H. boettgeri* and the microphagous filter feeding *X. laevis* (Seale et al. 1982; Sokol 1962) were included in our analysis (Figure 5). We also incorporated endotrophic tadpoles of terrestrial indirect‐developing *Eupsophus* (Vera Candioti et al. 2005, 2011) and an endotrophic embryo of the terrestrial direct‐developing *Eleutherodactylus coqui* (Westrick et al. 2022) (Figure 6). Assigning specific cranial topologies in larval/embryonic anurans to specific feeding or developmental modes requires broader taxonomic sampling. Endotrophic species show distinct alterations in their development, like the reduction of oral structures and modifications in the branchial basket (Altig and Johnston 1989; Kerney et al. 2010; Vera Candioti et al. 2011), which is reflected in their cranial topology. Studies on endotrophic species with a terrestrial indirect and direct development indicate that rather than a complete loss of the tadpole phase, their development can be viewed as a mosaic of ontogenetic rearrangements, including potential recapitulation or conservation, reduction, loss and heterochronic shifts of ancestral biphasic characters, along with morphological innovation (Callery et al. 2001; Wassersug 1975). In endotrophic tadpoles of *Eupsophus*, the cranial topology of an exotrophic ancestor appears to be conserved, congruent with the reported similarity of cranial morphology between *Eupsophus* tadpoles and exotrophic species (Vera Candioti et al. 2005, 2011).

In direct‐developing anurans, metamorphosis is not lost, but occurs during embryonic development and in a more gradual way compared to its climactic course in biphasic anurans (Callery et al. 2001). Several tadpole‐specific traits are lost in these species, including the development of cement glands, neuromasts, the lateral line system and keratinous beaks (Fang and Elinson 1996, 1999; Schlosser et al. 1999). Other tadpole‐specific traits, such as rudimentary gills, the operculum, the tail, specialized neurons and aspects of skin development, as well as aspects of cranial ontogeny, are maintained to varying degrees (Callery et al. 2001; Callery and Elinson 2000; Goldberg et al. 2020; Hanken et al. 1992; Naumann et al. 2021; Schlosser and Roth 1997; Schweiger et al. 2017). Notably, cranial and musculoskeletal traits are reduced to a state resembling that of an exotrophic tadpole at the metamorphic climax, for example the rod‐shaped palatoquadrate or the form of the hyoid (Elinson 2007; Hanken et al. 1992, 1997). In the topological morphospace, crania of endotrophic embryos/larvae cluster closely with exotrophic species, showing a high similarity of topological parameters. This pattern does not necessarily show a shared underlying cranial topology between exotrophic and endotrophic species. Rather, it could indicate that exotrophic and endotrophic crania are both highly integrated systems, either because of a functional integration (feeding, respiration, locomotion) in exotrophic species or developmental processes in the larva/embryo of endotrophic species.

### 4.3 Anatomical Network Analysis Supports the Adaptive Decoupling Hypothesis for the Crania of Pre‐ and Post‐Metamorphic Anurans

Studying species with complex life cycles raises the question to what degree traits separated by metamorphosis coevolve. This question is captured within the ADH, which proposes that evolutionary adaptations of distinct phases of complex life cycles are largely decoupled from each other (Istock 1967; Moran 1994).

We interpret the clear distinction between pre‐ and post‐metamorphic cranial topologies in anurans as support for the ADH. In tadpoles, the high integration and low modularity of the cranium might reflect the tight coupling of feeding and respiration, facilitated by skeletal structures shared across the cranial system (Wassersug and Hoff 1979). Because of this integration, morphological changes to individual skeletal elements tend to affect the entire cranial system rather than remain confined to specific areas. Given the great diversity of larval feeding modes, with many linked to specific cranial adaptations (Altig and Johnston 1989; Montaña et al. 2019), whole‐system changes may have driven this diversification and could help explain the high morphological disparity in the crania of pre‐metamorphic anurans.

In adult anurans, by contrast, the high modularity of the cranium allows individual modules to specialize somewhat independently. This may have facilitated the evolution of distinct feeding mechanisms, without requiring major changes to the rest of the cranial system, as seen, for example, in the evolution of aquatic suction feeding in Pipidae, which differs strongly from terrestrial feeding involving a tongue and biting jaws (Carreño and Nishikawa 2010; Sokol 1969). Modular organization may have similarly allowed specializations of sensory structures and their associated cranial elements, consistent with evidence in cichlid fishes that brain and sensory organ morphology can shape cranial structures (Conith et al. 2023). Semi‐independent specialization of sensory organs and associated cranial structures might have coevolved with shifts in activity patterns and habitat use without substantially interfering with other cranial functions. Altogether, this modularity may have permitted specializations across different parts of the post‐metamorphic cranium without negatively affecting the entire cranial system, and may thereby have supported the diversification of the anurans.

Support for ADH has also been shown in the closely related Caudata, based on morphometric analyses of the cranium and mandible (Fabre et al. 2020; Louppe et al. 2025). Studies regarding body form and vertebral column diversification, by contrast, show correlations between life phases (Bonett and Blair 2017). In anurans specifically, evidence for adaptive decoupling is more ambiguous. Correlated evolution across life phases has been suggested on the basis of the genetic background in body size and locomotor traits (Watkins 2001), as well as behavioral continuity across metamorphosis (Wilson and Krause 2012). Studies of size traits further support correlation between life phases, although Dehling and Sinsch (2019) note that correlations between life phases may depend on taxon sampling and phylogenetic scale. Extended analyses of size traits, however, reveal less consistent patterns (Phung et al. 2020). Blouin (1992) found that the genetic basis of whole‐body growth and differentiation showed both correlated and decoupled traits within the same study, highlighting that correlation and decoupling are not exclusive. Overall, the literature tends to support adaptive decoupling, with respect to some aspects of the genetic basis of morphology (Phillips 1998; Wollenberg Wollenberg Valero et al. 2017), locomotor traits (Johansson et al. 2010), and body shape changes (Almeida‐Silva and Vera Candioti 2024; Sherratt et al. 2017). Together, these findings suggest that while correlations between life phases do exist, decoupling of traits plays an important role in anuran evolution. Adaptive decoupling in anurans operates thereby on individual traits rather than entire life phases. The distinct topologies of tadpoles/embryos and adults, consistent across species with different feeding and reproductive modes, provide morphological evidence for the adaptive decoupling of the cranial system.

## 5 Conclusions

This study provides morphological descriptions of the cranial musculoskeletal system of larval and adult *Smilisca phaeota* and applies Anatomical Network Analysis (AnNA) to quantify changes in cranial topology across metamorphosis, complementing established morphometric analyses (Bardua et al. 2020) by examining the organization of the cranial system. Both topological and developmental‐topological analyses reveal consistent metamorphic changes across all examined species, with the topological analysis recovering more pronounced topological shifts. Tadpole crania are characterized by high integration and low modularity, whereas adult crania are considerably more modular. The topological distinction between life phases, observed across species with different feeding and developmental modes, supports the adaptive decoupling of the anuran cranial system during ontogeny. Our results reinforce the idea that metamorphosis enabled the largely independent evolution of larval and adult traits and the specialized adaptations with each life stage.

## Author Contributions

**Lorenz Guschker:** data curation, formal analysis, investigation, methodology, software, validation, visualization, writing – original draft preparation. **Maximilian A. Stüwe:** data curation, methodology, software, validation, writing – review and editing. **Hendrik Müller:** conceptualization, supervision, validation, writing – review and editing. **Benjamin Naumann:** conceptualization, methodology, project administration, resources, supervision, validation, writing – review and editing.

## Funding

The authors have nothing to report.

## Conflicts of Interest

The authors declare no conflicts of interest.

## Supporting information


Supporting File 1

Supporting File 2

Supporting File 3

Supporting File 4

## Data Availability

The data that support the findings of this study are available in the supporting material of this article. 3D‐models of larval and adult *Smilisca phaeota* are available in Supporting Material S4. Original data used and/or analyzed during the current study are available from the corresponding authors on request.
