# Supplementary material for: Anatomical Network Analyses of Pre‐ and Post‐Metamorphic Cranial Systems Support the Adaptive Decoupling Hypothesis in Anura
Source: J Morphol. 2026 Oct 29;287(11):e70185. doi: 10.1002/jmor.70185 (PMC13717468; doi:10.1002/jmor.70185)

Supplementary Material S1

Figures S1.1 Dendrograms of developmental-topology networks.

Figures S1.2 Dendrograms of topology networks.

Figure S1.3 Principal Component Analysis of network parameters (N,K,D,C,L,H,P) from **A** topological and **B** developmental-topological analyses of adult and larval/embryonic cranial musculoskeletal system. N and K were redimensioned by taking their square root following Rawson et al. (2022). **C** Schematic illustration of the anatomical network parameters used in the analyses, modified from Esteve-Altava et al. (2018).

Figure S1.4 Topology networks and identified Q-modules of **A** larval and **B** adult *Smilisca phaeota*. Developmental-topology networks with identified Q-modules of **C** larval and **D** adult *Smilisca phaeota*. Abbreviations of the skeletal elements for the networks are found in Table 5.

Figure S1.5 Anatomical networks and connectivity modules (Q-modules) of *Ascaphus truei* (**A**-**D**) and *Bombina orientalis* (**E**-**H**), shown for larvae (**A**,**C**,**E**,**G**) and adults (**B**,**D**,**F**,**H**). For each species, the upper network represents topological analysis (**A**,**B**,**E**,**F**) and the lower network represents development-topological analysis (**C**,**D**,**G**,**H**). Abbreviations of the skeletal elements for the networks are found in Table 5.

Figure S1.6 Anatomical networks and connectivity modules (Q-modules) of *Hymenochirus boettgeri* (**A**-**D**) and *Xenopus laevis* (**E**-**H**), shown for larvae (**A**,**C**,**E**,**G**) and adults (**B**,**D**,**F**,**H**). For each species, the upper network represents topological analysis (**A**,**B**,**E**,**F**) and the lower network represents development-topological analysis (**C**,**D**,**G**,**H**). Abbreviations of the skeletal elements for the networks are found in Table 5.

Figure S1.7 Anatomical networks and connectivity modules (Q-modules) of embryonic (**A**,**C**) and adult *Eleutherodactylus coqui* (**B**,**D**), larval *Eupsophus calcaratus* (**E**,**G**), adult *Eupsophus roseus* (**F**,**J**) and larval *Eupsophus emiliopugini* (**H**,**K**). For each species, the upper network represents topological analysis (**A**,**B**,**E**,**F**,**H**) and the lower network represents development-topological analysis (**C**,**D**,**G**,**J**,**K**). Abbreviations of the skeletal elements for the networks are found in Table 5.

Figure S1.8 Current phylogeny of anurans following Liedtke et al. (2022). Highlighted families were included in taxon sampling.

References mentioned in Supplementary Material:

Esteve-Altava, B., Molnar, J. L., Johnston, P., Hutchinson, J. R., & Diogo, R. (2018). Anatomical network analysis of the musculoskeletal system reveals integration loss and parcellation boost during the fins-to-limbs transition. *Evolution*, *72*(3), 601-618.

Liedtke, H. C., Wiens, J. J., & Gomez-Mestre, I. (2022). The evolution of reproductive modes and life cycles in amphibians. *Nature communications*, *13*(1), 7039.

Rawson, J. R., Esteve-Altava, B., Porro, L. B., Dutel, H., & Rayfield, E. J. (2022). Early tetrapod cranial evolution is characterized by increased complexity, constraint, and an offset from fin-limb evolution. *Science Advances*, *8*(36), eadc8875.

Figures S1.1 Dendrograms of topology networks.

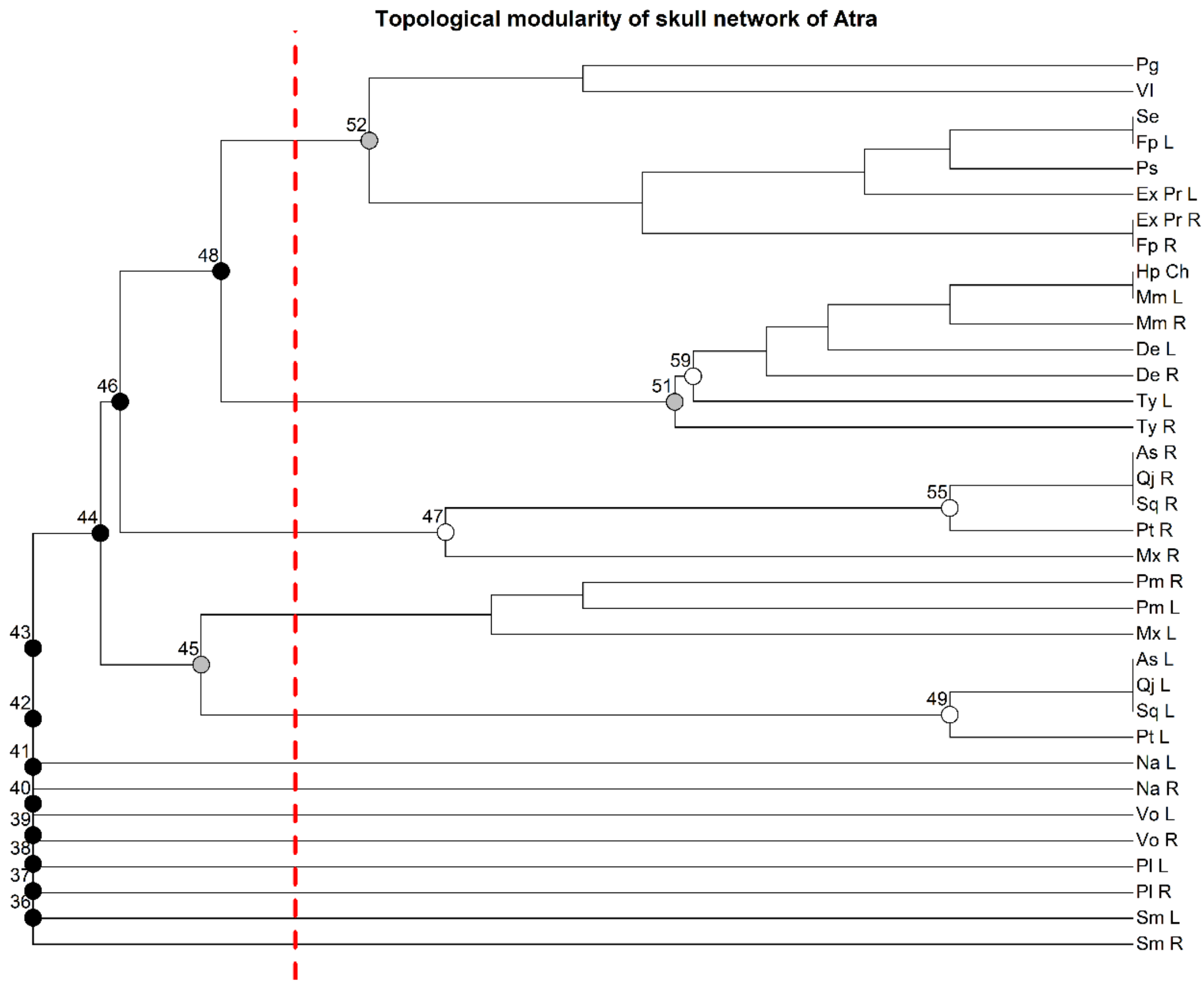

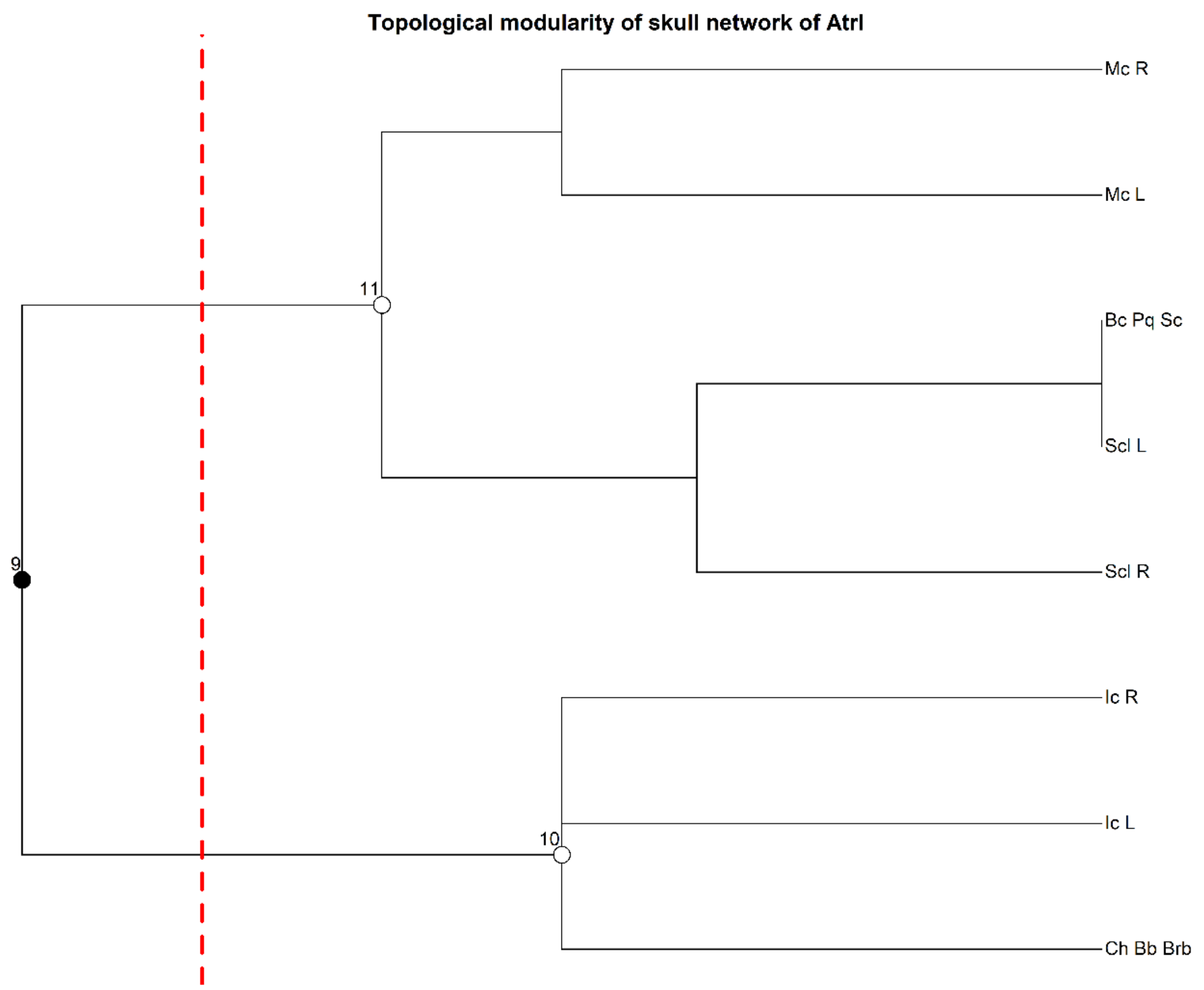
Topological modularity of skull network of Atrl
Mc R
Mc L
11
Bc Pq Sc
Scl L
9
Scl R
Ic R
Ic L
10
Ch Bb Brb

Topological modularity of skull network of Bora

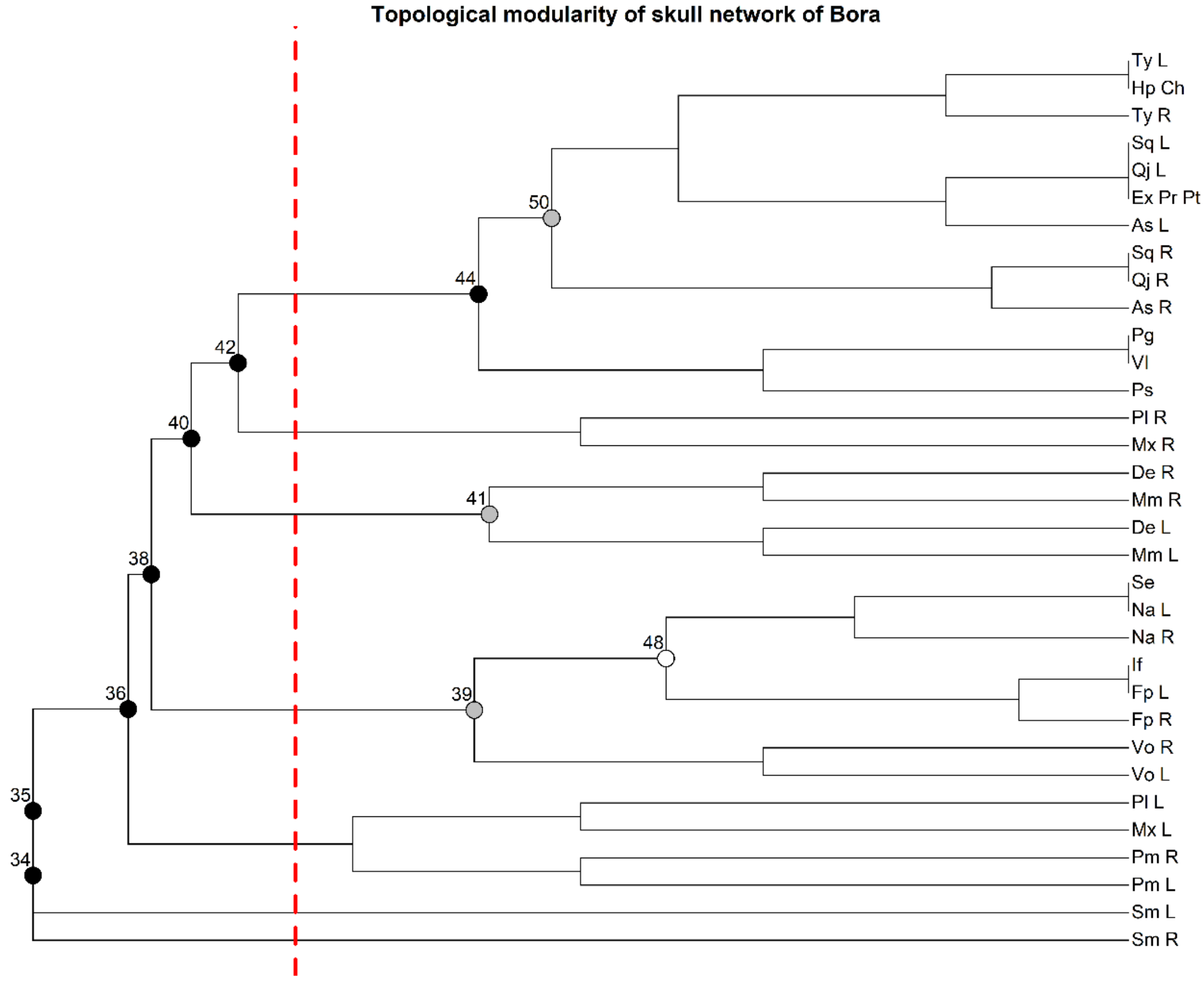

Topological modularity of skull network of BorI

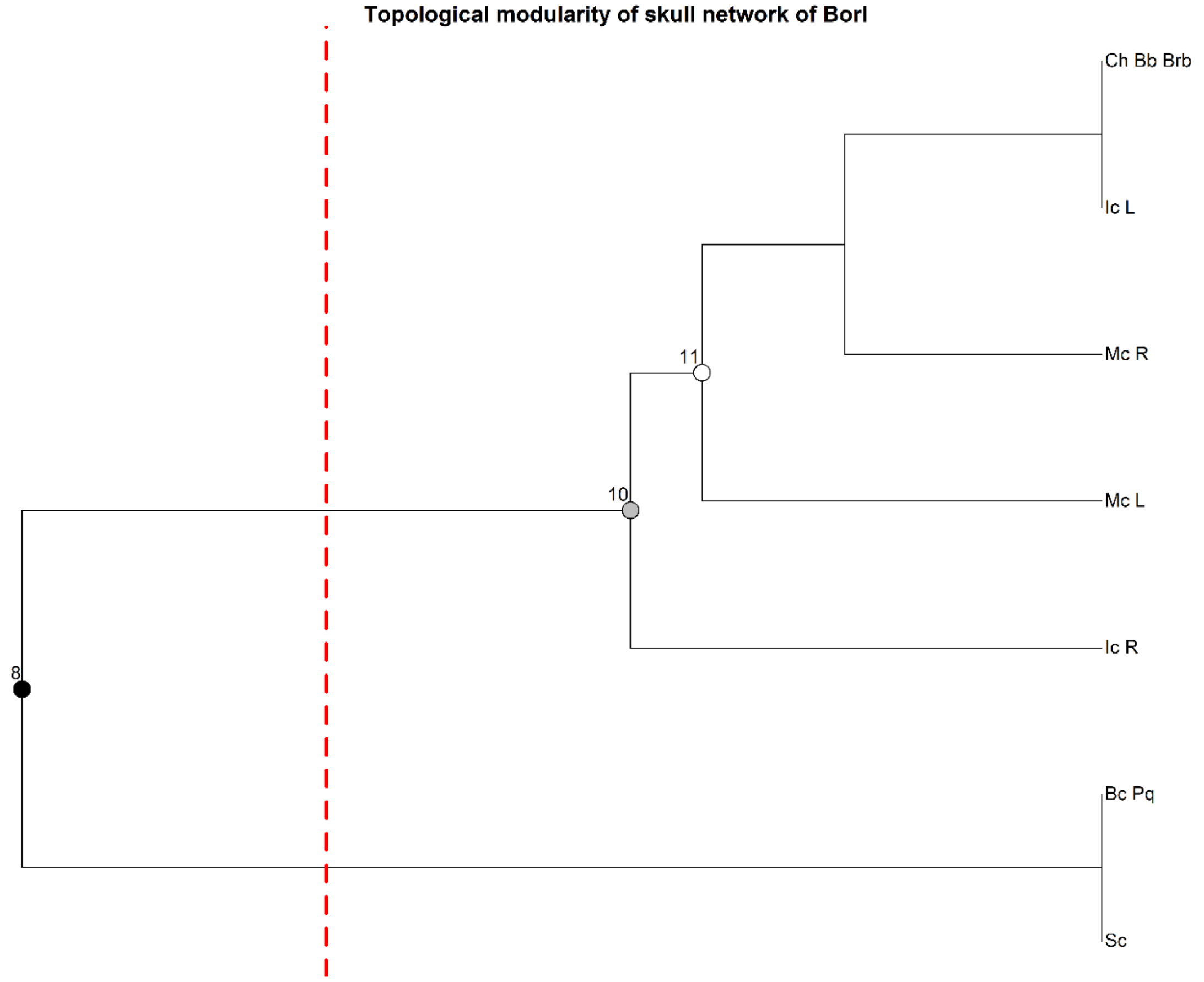

Topological modularity of skull network of Ecoa

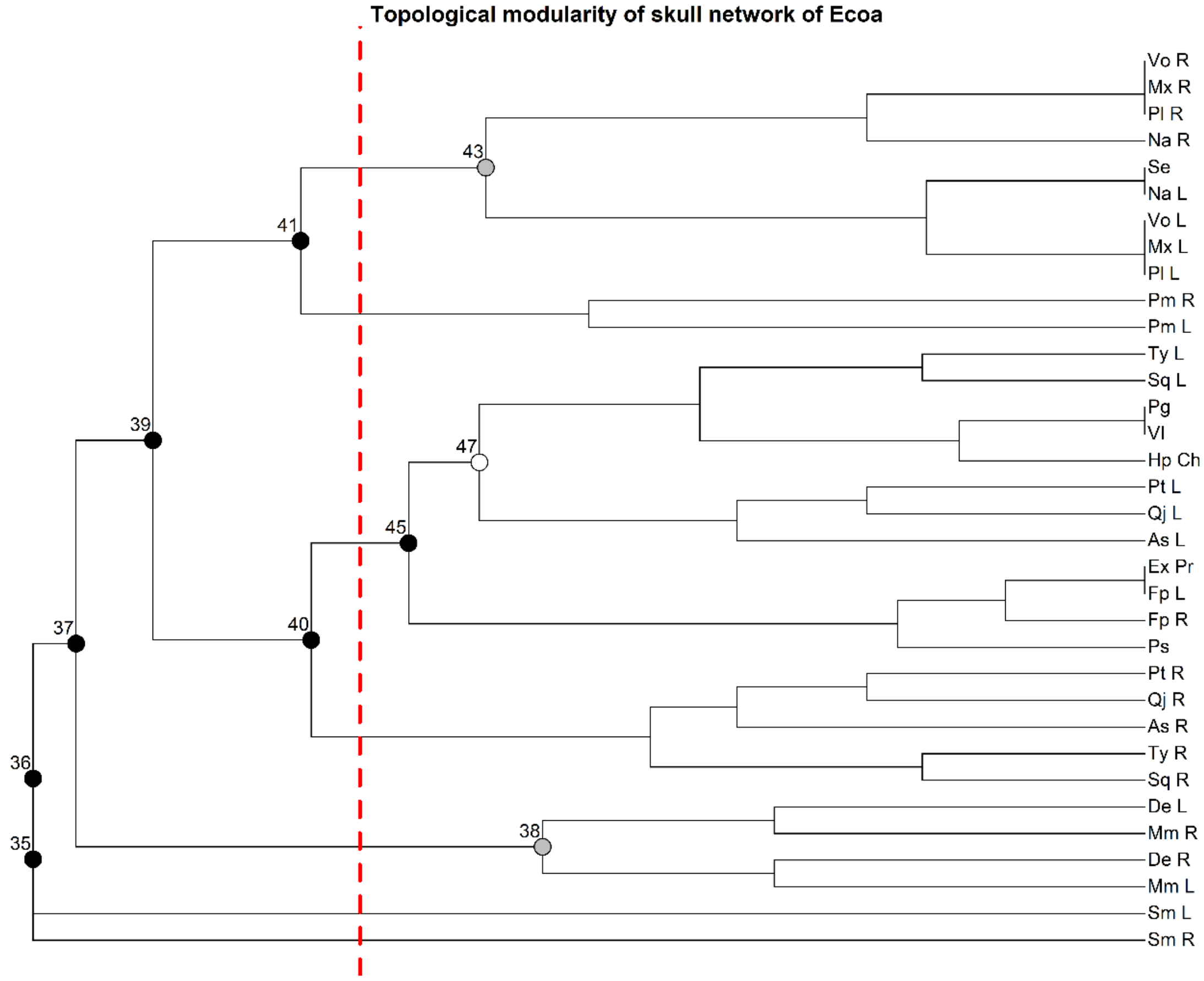

Topological modularity of skull network of Ecol

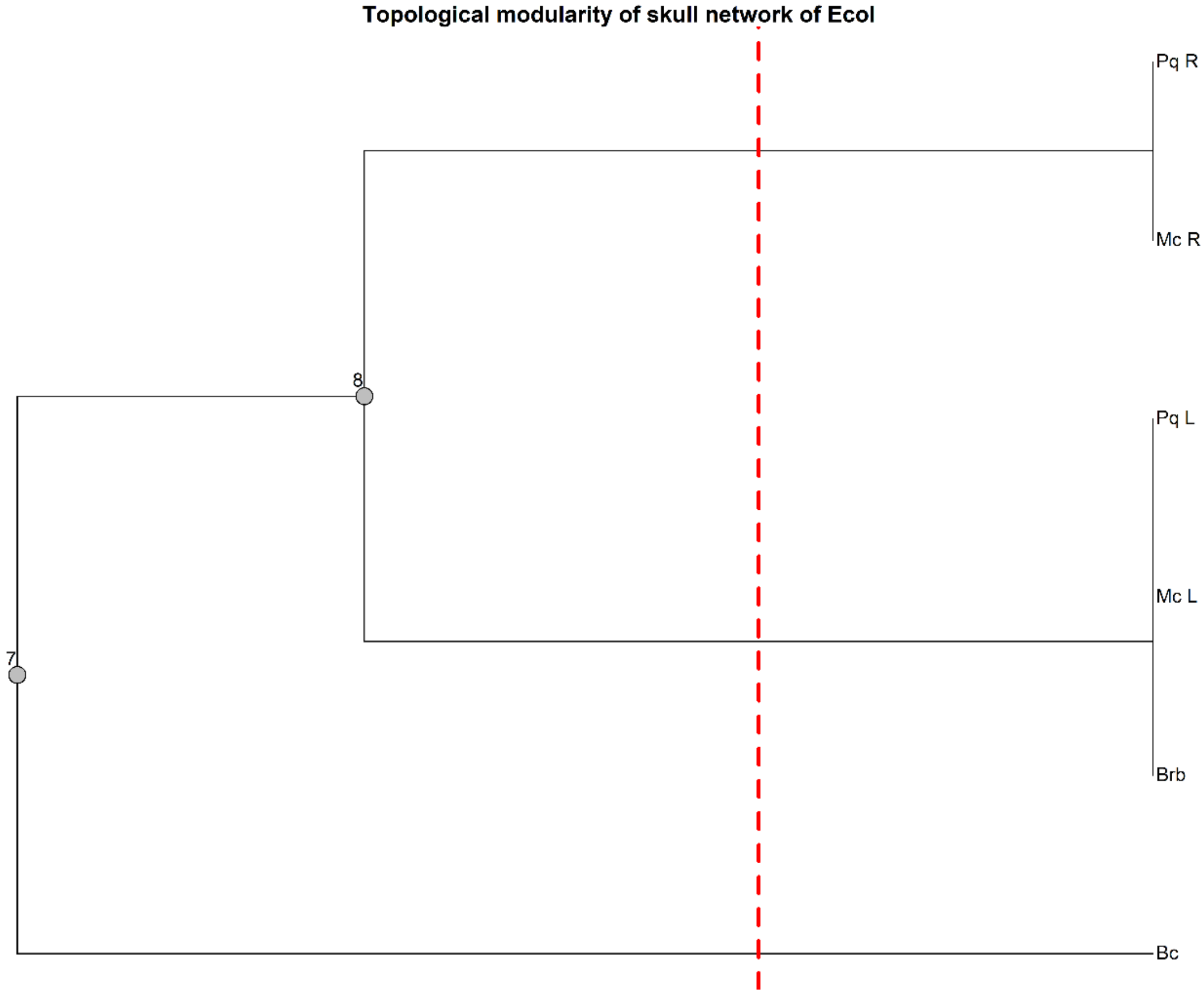

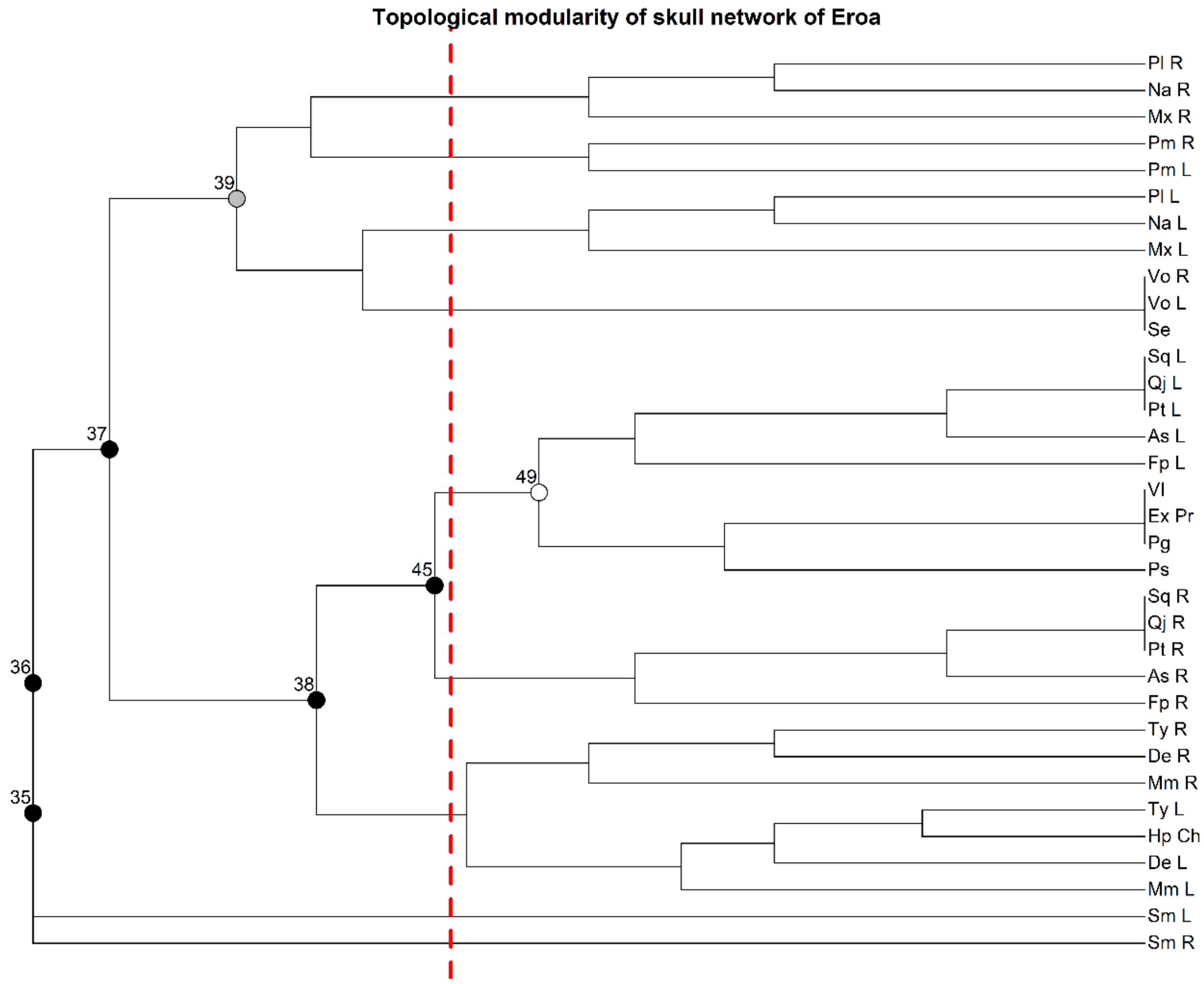
Topological modularity of skull network of Eroa
Pl R
Na R
Mx R
Pm R
Pm L
Pl L
Na L
Mx L
Vo R
Vo L
Se
Sq L
Qj L
Pt L
As L
Fp L
Vl
Ex Pr
Pg
Ps
Sq R
Qj R
Pt R
As R
Fp R
Ty R
De R
Mm R
Ty L
Hp Ch
De L
Mm L
Sm L
Sm R
39
37
49
45
36
38
35

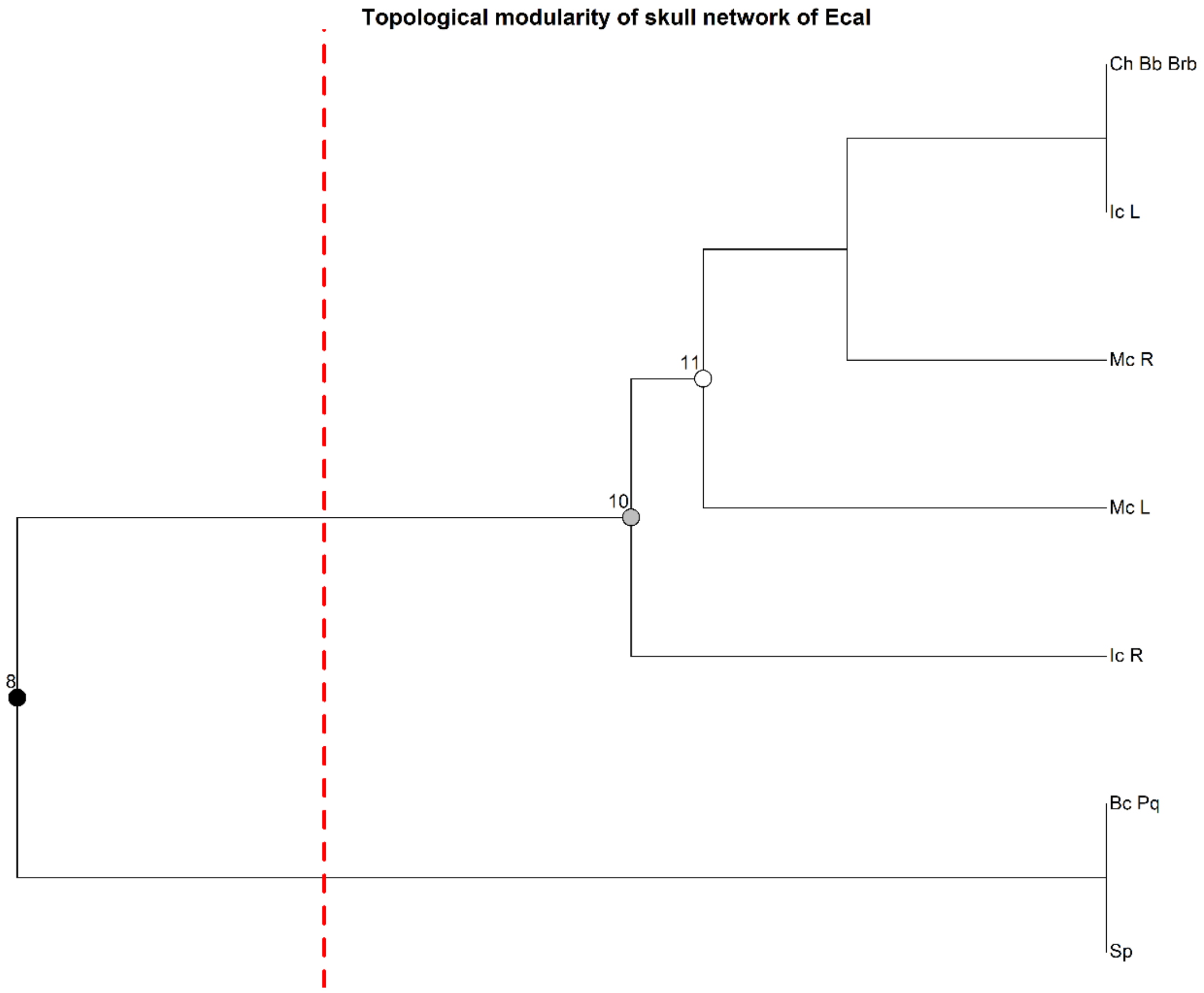
Topological modularity of skull network of Ecal
Ch Bb Brb
Ic L
Mc R
11
Mc L
10
Ic R
8
Bc Pq
Sp

Topological modularity of skull network of Eeml

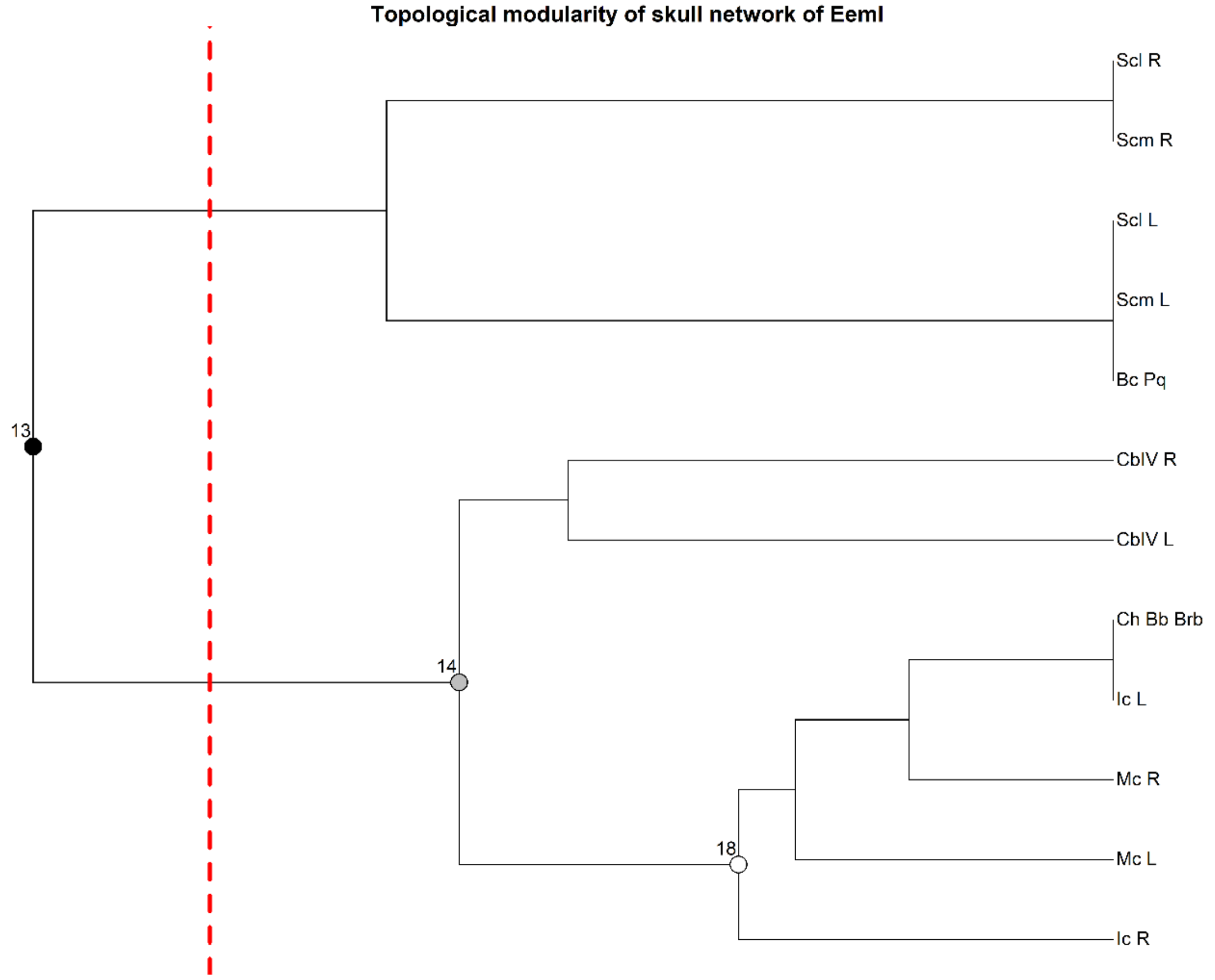

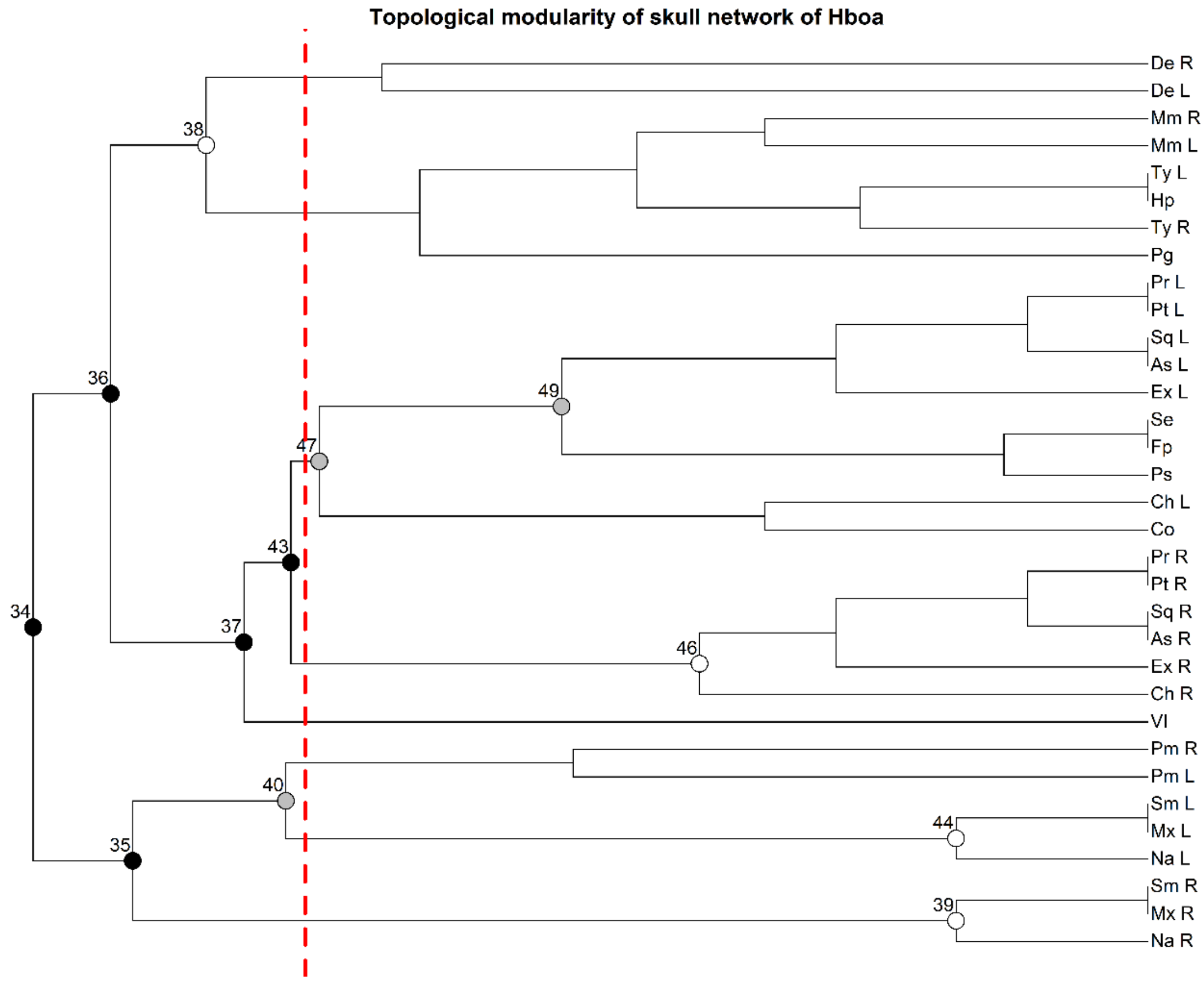
Topological modularity of skull network of Hboa
38
36
34
37
43
47
49
46
40
35
44
39
De R
De L
Mm R
Mm L
Ty L
Hp
Ty R
Pg
Pr L
Pt L
Sq L
As L
Ex L
Se
Fp
Ps
Ch L
Co
Pr R
Pt R
Sq R
As R
Ex R
Ch R
Vl
Pm R
Pm L
Sm L
Mx L
Na L
Sm R
Mx R
Na R

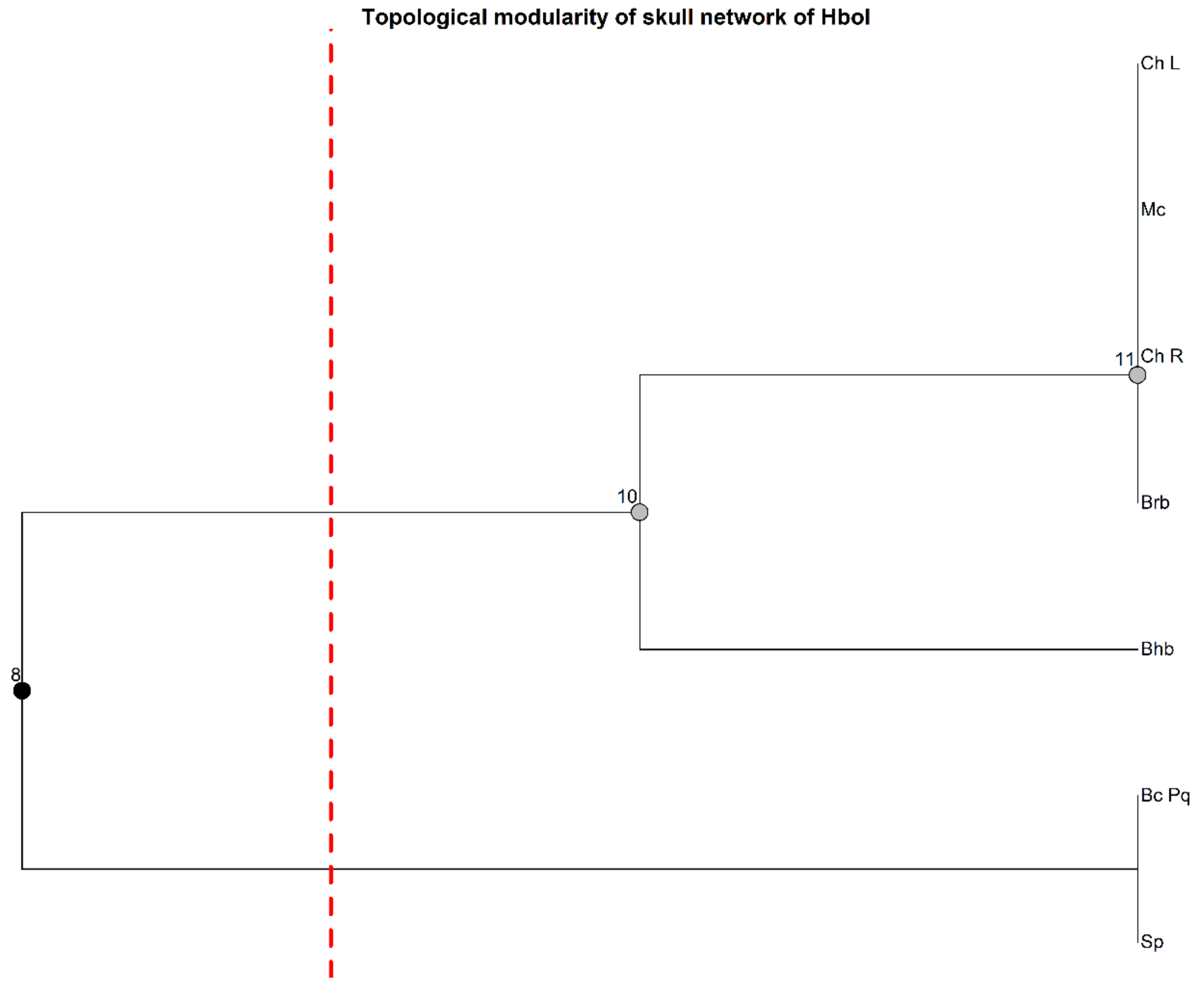
Topological modularity of skull network of Hbol
Ch L
Mc
11
Ch R
10
Brb
Bhb
8
Bc Pq
Sp

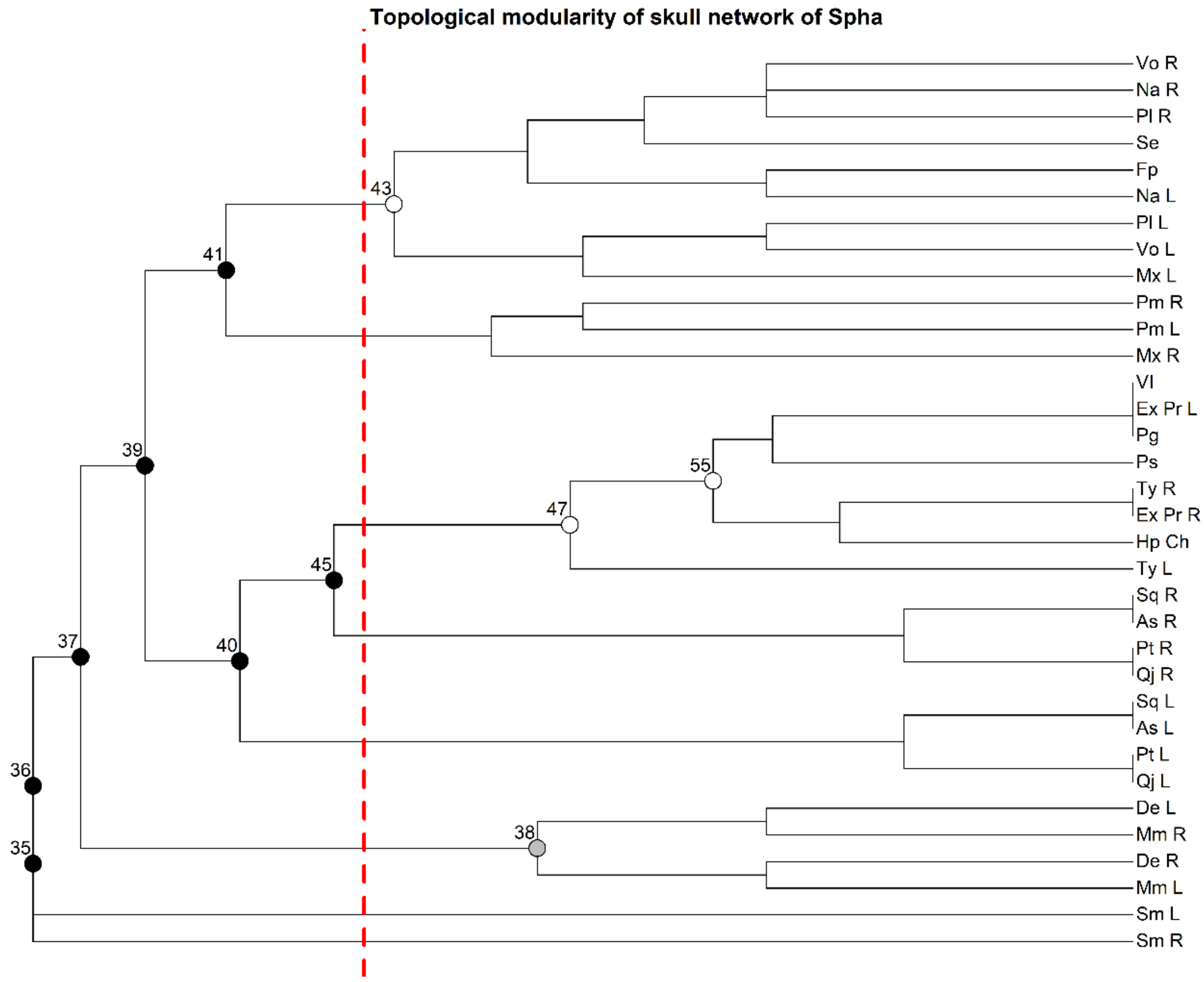
Topological modularity of skull network of Spha
Vo R
Na R
Pl R
Se
Fp
Na L
Pl L
Vo L
Mx L
Pm R
Pm L
Mx R
Vl
Ex Pr L
Pg
Ps
Ty R
Ex Pr R
Hp Ch
Ty L
Sq R
As R
Pt R
Qj R
Sq L
As L
Pt L
Qj L
De L
Mm R
De R
Mm L
Sm L
Sm R
43
41
39
55
47
45
37
40
36
38
35

Topological modularity of skull network of SphI

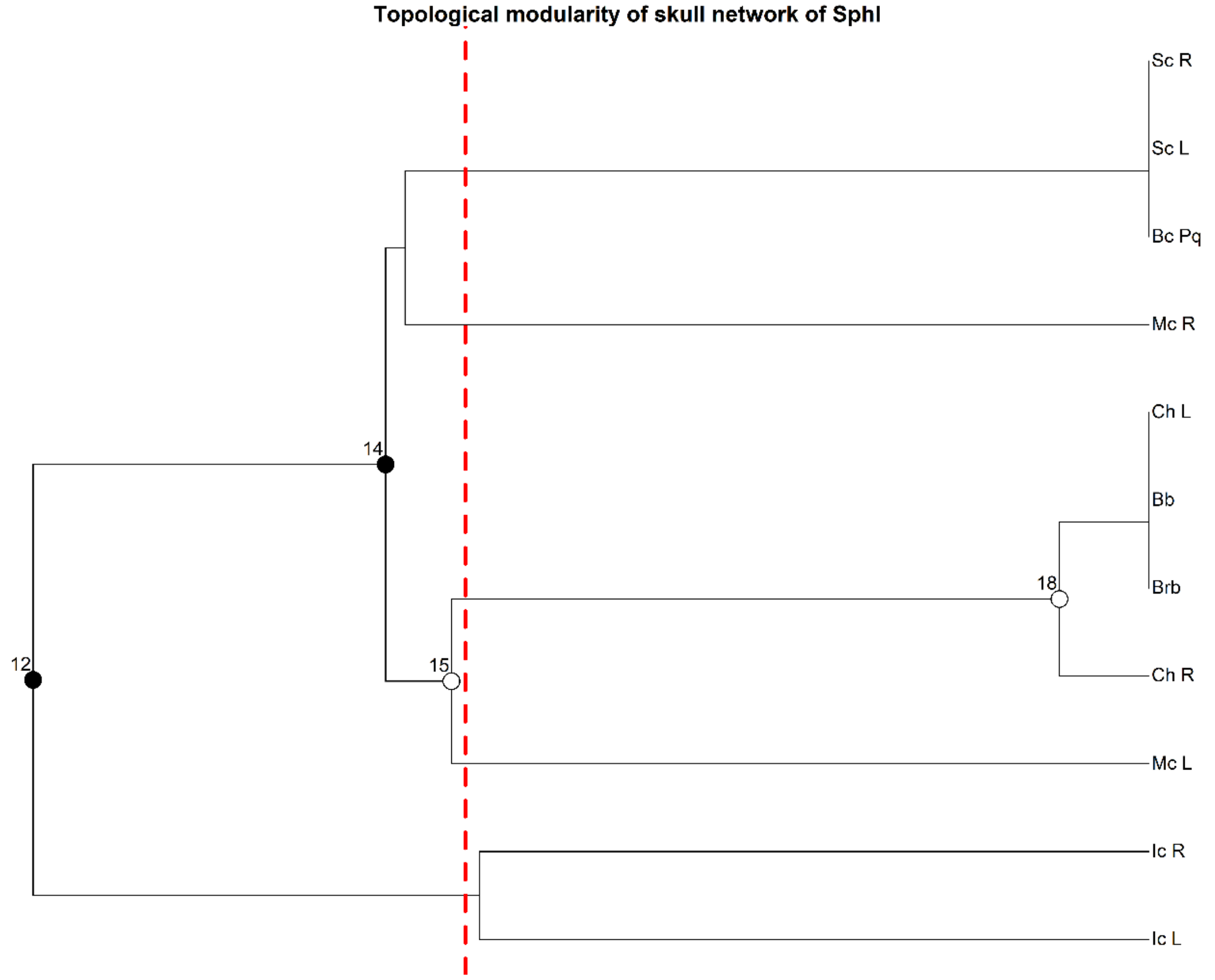

Topological modularity of skull network of Xlaa

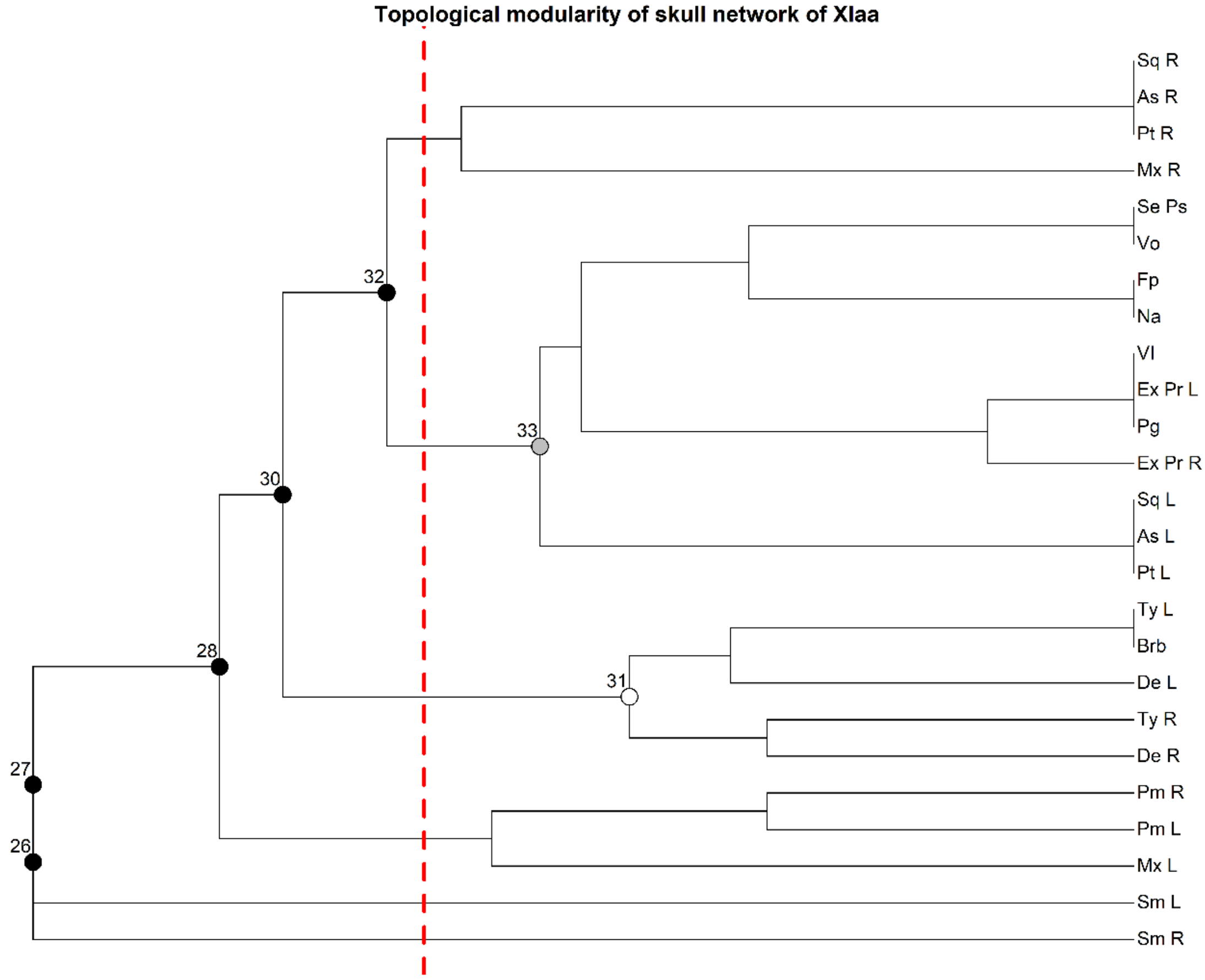

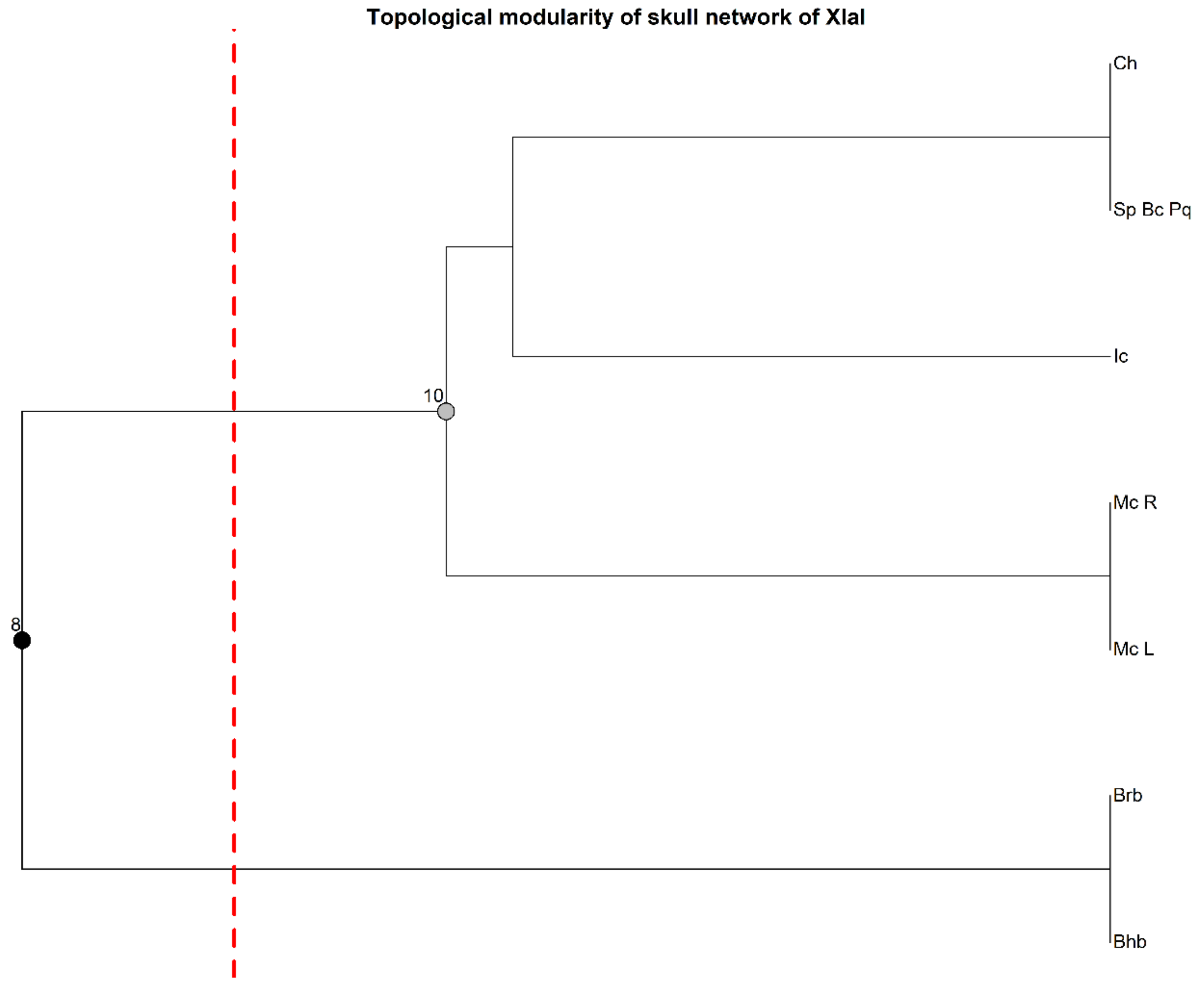
Topological modularity of skull network of Xlal
Ch
Sp Bc Pq
Ic
10
Mc R
8
Mc L
Brb
Bhb

Figures S1.2 Dendrograms of developmental-topology networks.

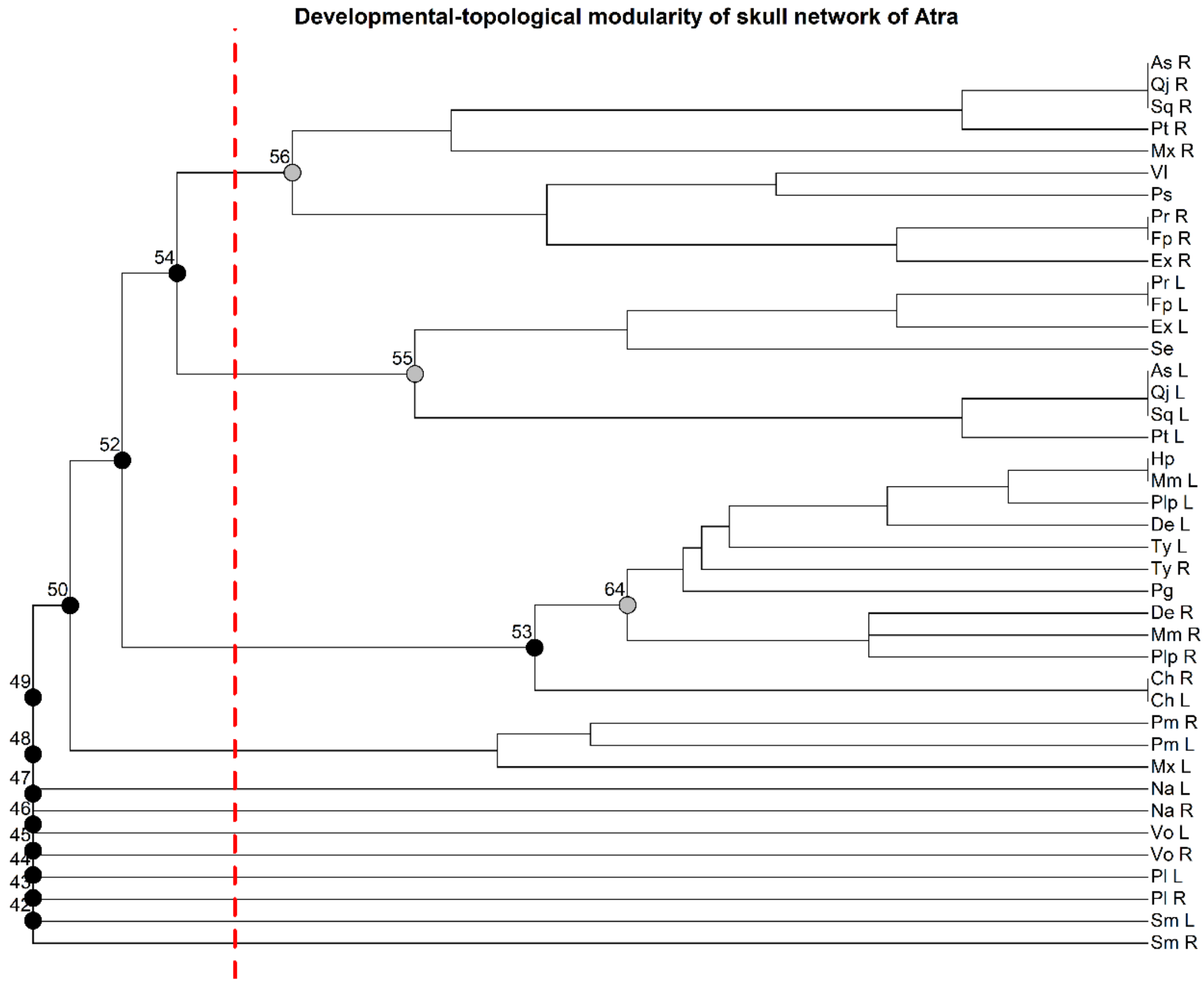

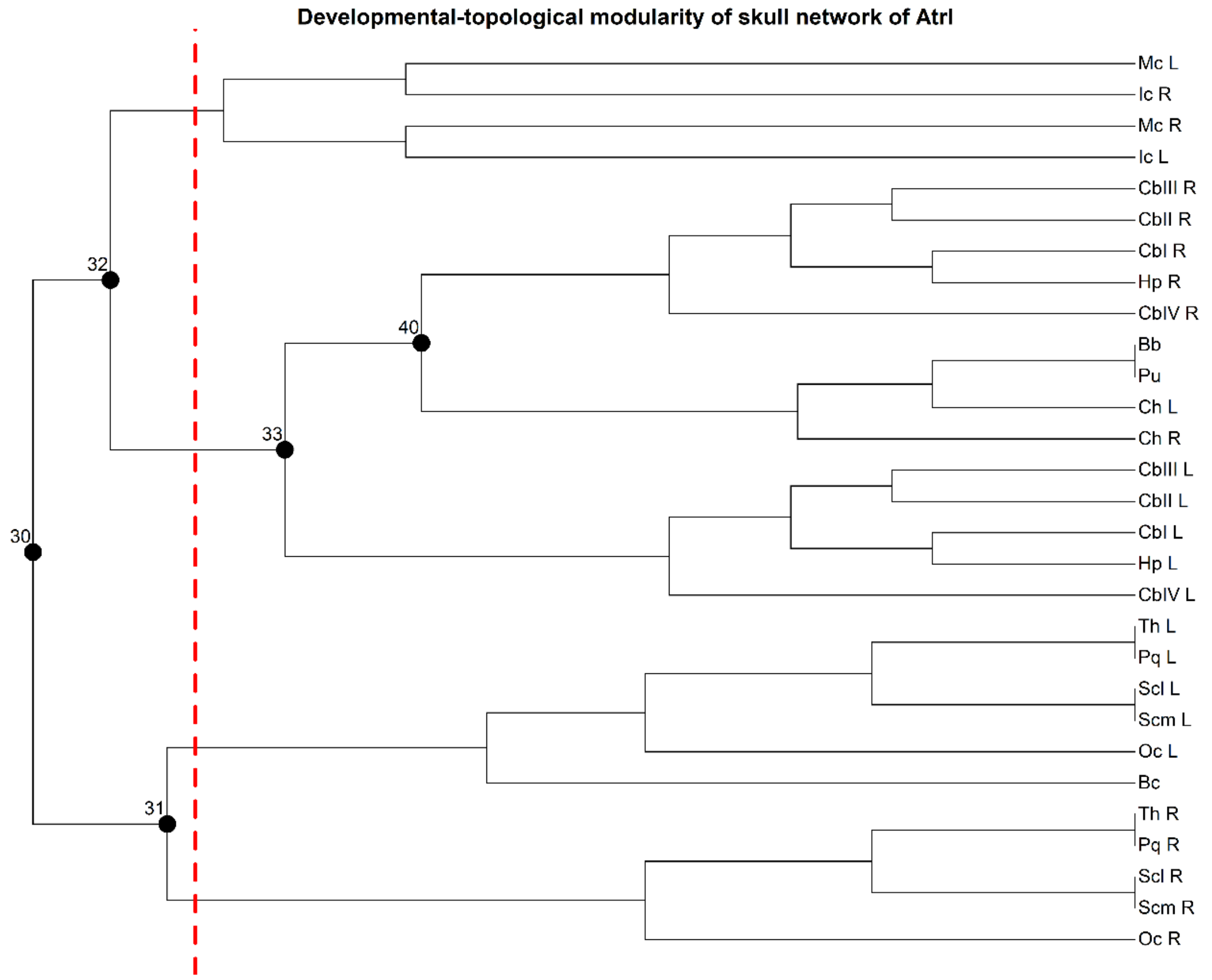
Developmental-topological modularity of skull network of Atrl
Mc L
Ic R
Mc R
Ic L
CbIII R
CbII R
CbI R
Hp R
CbIV R
Bb
Pu
Ch L
Ch R
CbIII L
CbII L
CbI L
Hp L
CbIV L
Th L
Pq L
Scl L
Scm L
Oc L
Bc
Th R
Pq R
Scl R
Scm R
Oc R
30
31
32
33
40

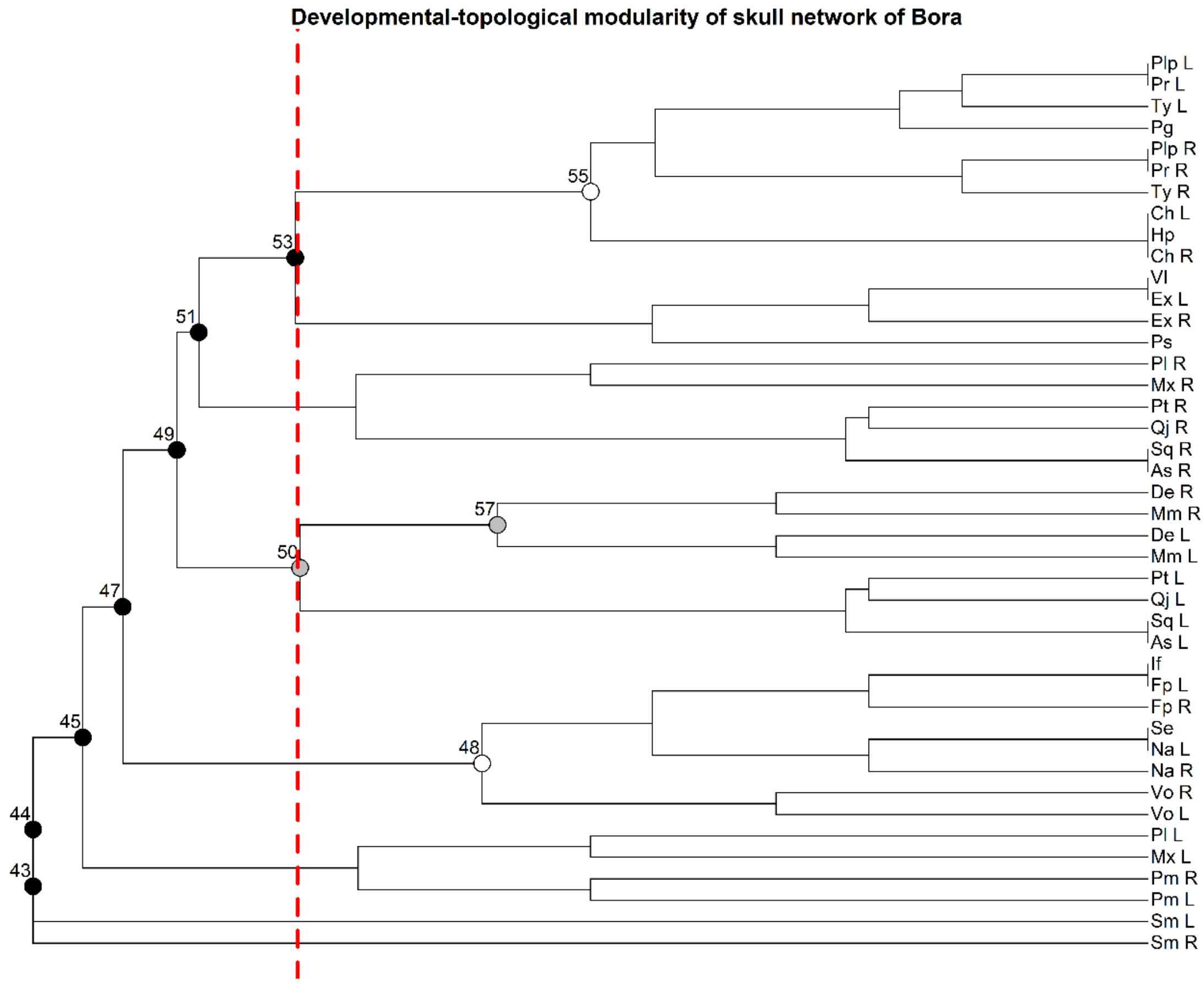

Developmental-topological modularity of skull network of Bora
55
53
51
49
57
50
47
45
48
44
43
Plp L
Pr L
Ty L
Pg
Plp R
Pr R
Ty R
Ch L
Hp
Ch R
Vl
Ex L
Ex R
Ps
Pl R
Mx R
Pt R
Qj R
Sq R
As R
De R
Mm R
De L
Mm L
Pt L
Qj L
Sq L
As L
If
Fp L
Fp R
Se
Na L
Na R
Vo R
Vo L
Pl L
Mx L
Pm R
Pm L
Sm L
Sm R

Developmental-topological modularity of skull network of Borl

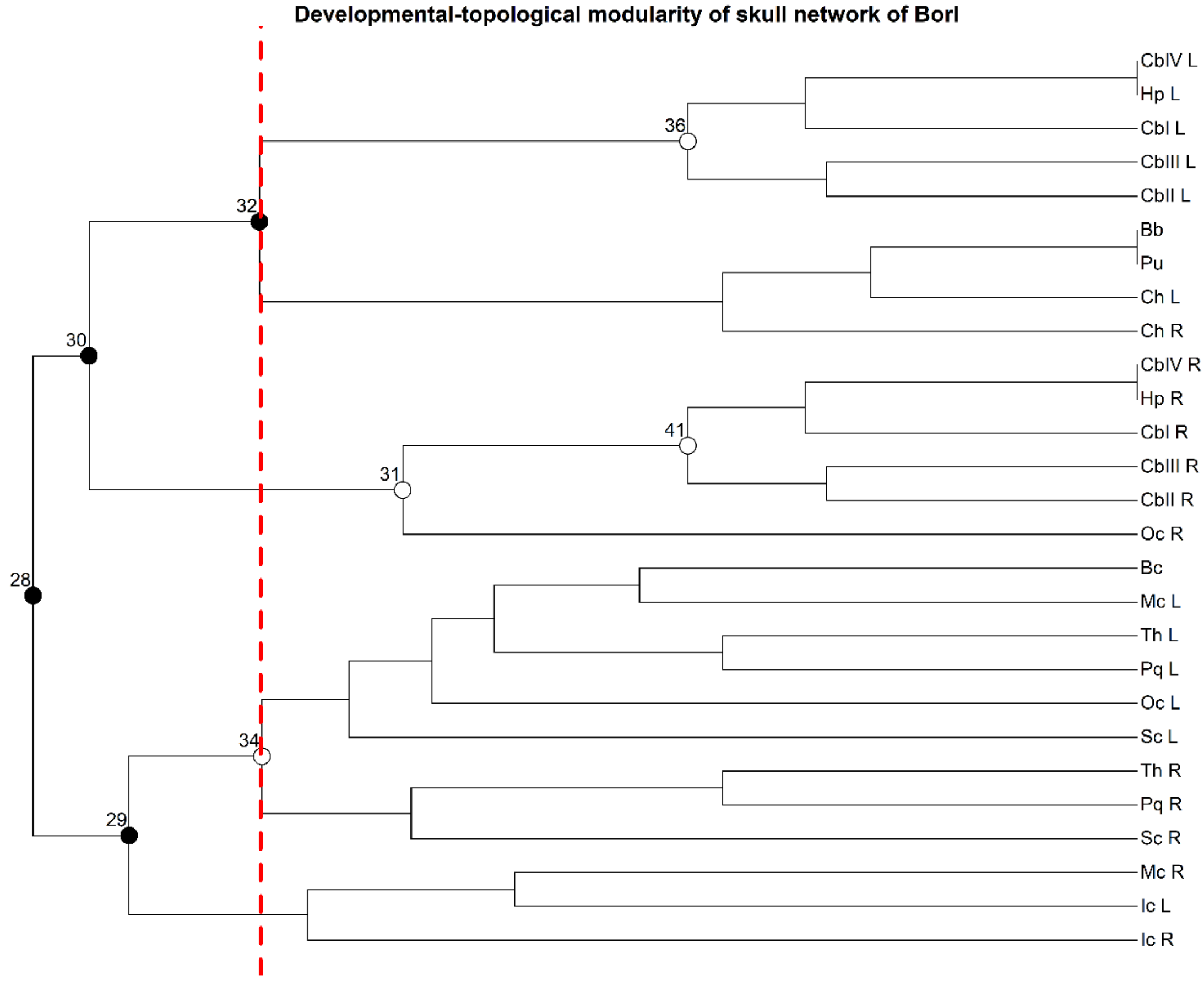

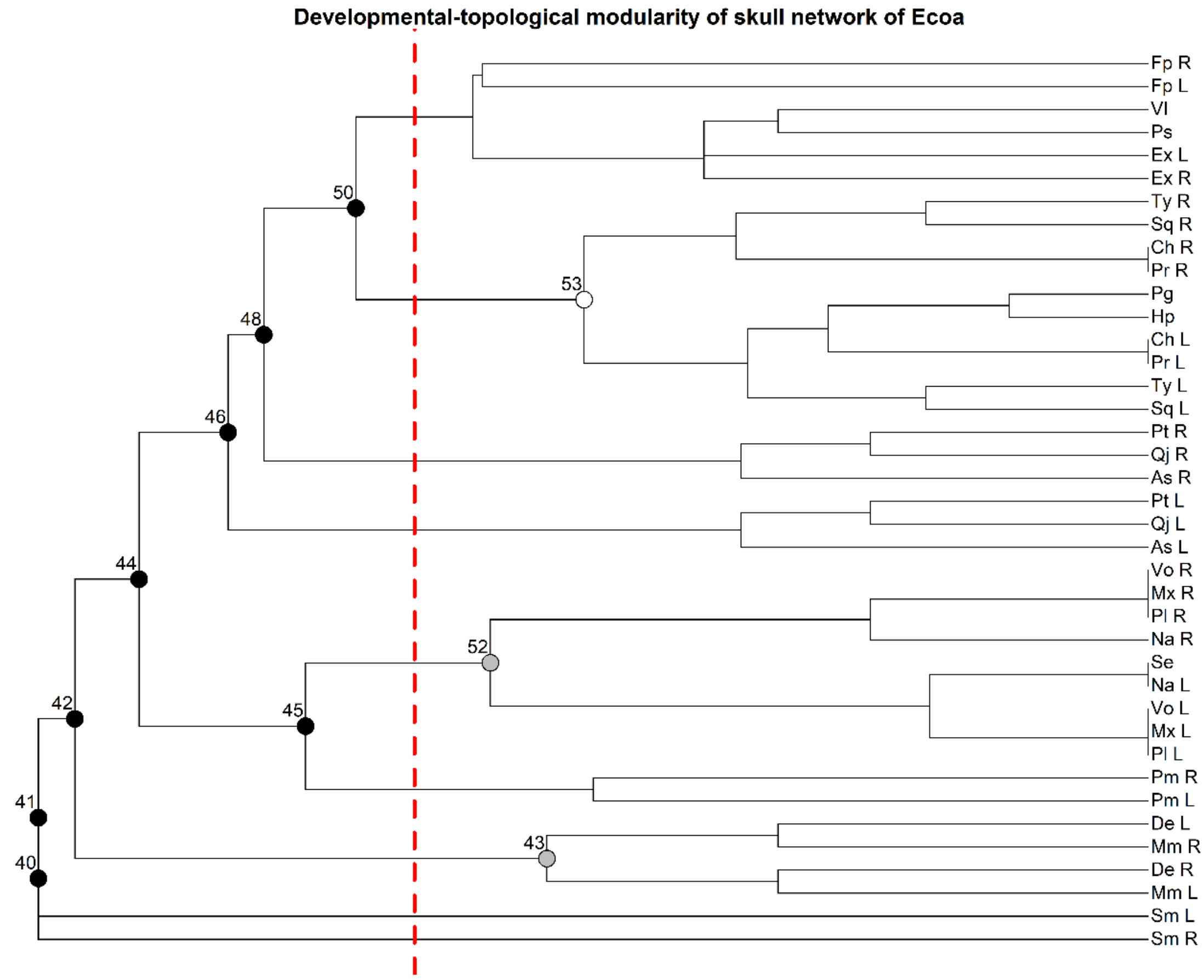

Developmental-topological modularity of skull network of Ecoa
Fp R
Fp L
Vl
Ps
Ex L
Ex R
Ty R
Sq R
Ch R
Pr R
Pg
Hp
Ch L
Pr L
Ty L
Sq L
Pt R
Qj R
As R
Pt L
Qj L
As L
Vo R
Mx R
Pl R
Na R
Se
Na L
Vo L
Mx L
Pl L
Pm R
Pm L
De L
Mm R
De R
Mm L
Sm L
Sm R
50
53
48
46
44
52
42
45
41
43
40

Developmental-topological modularity of skull network of Ecol

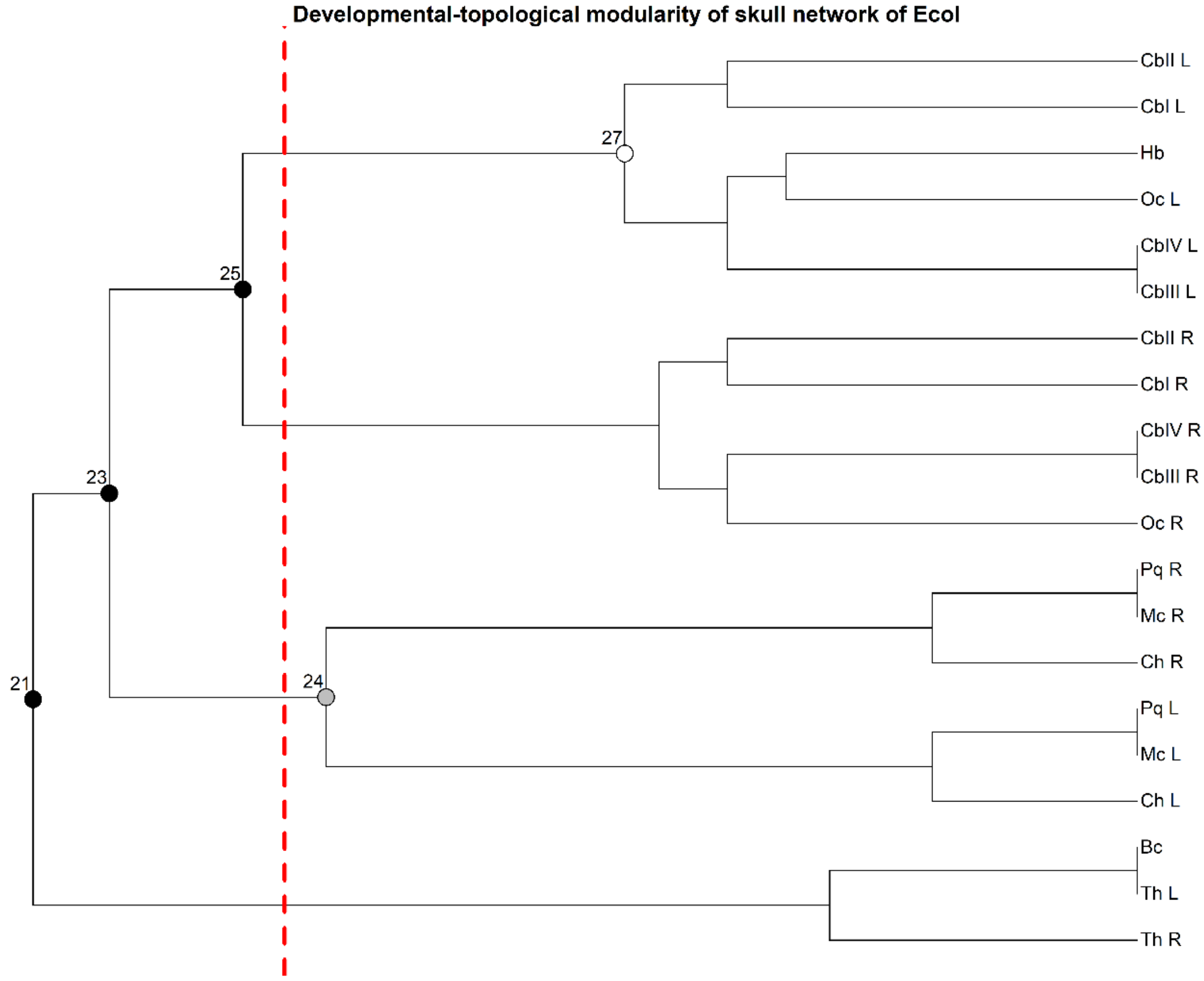

Developmental-topological modularity of skull network of Eroa

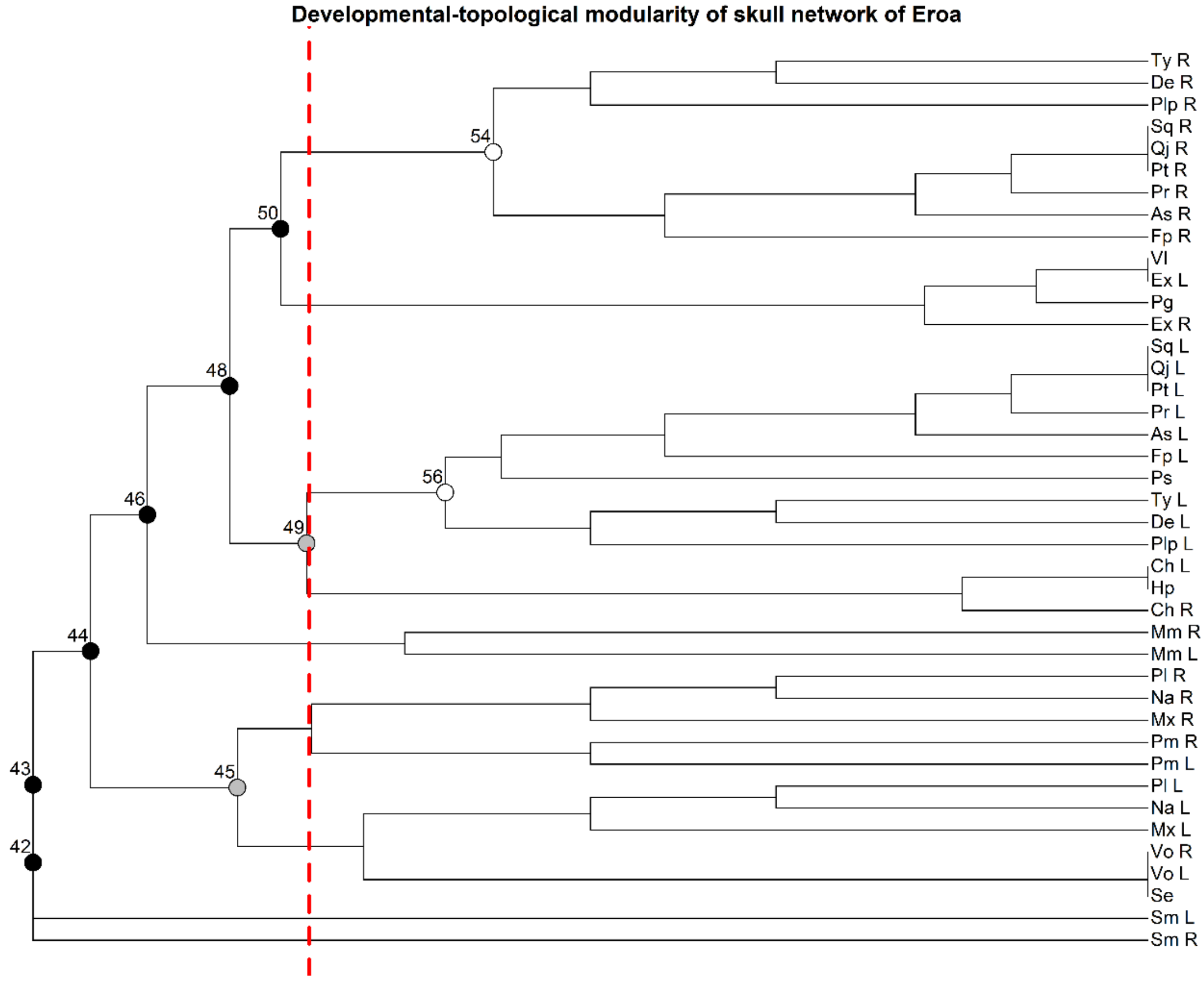

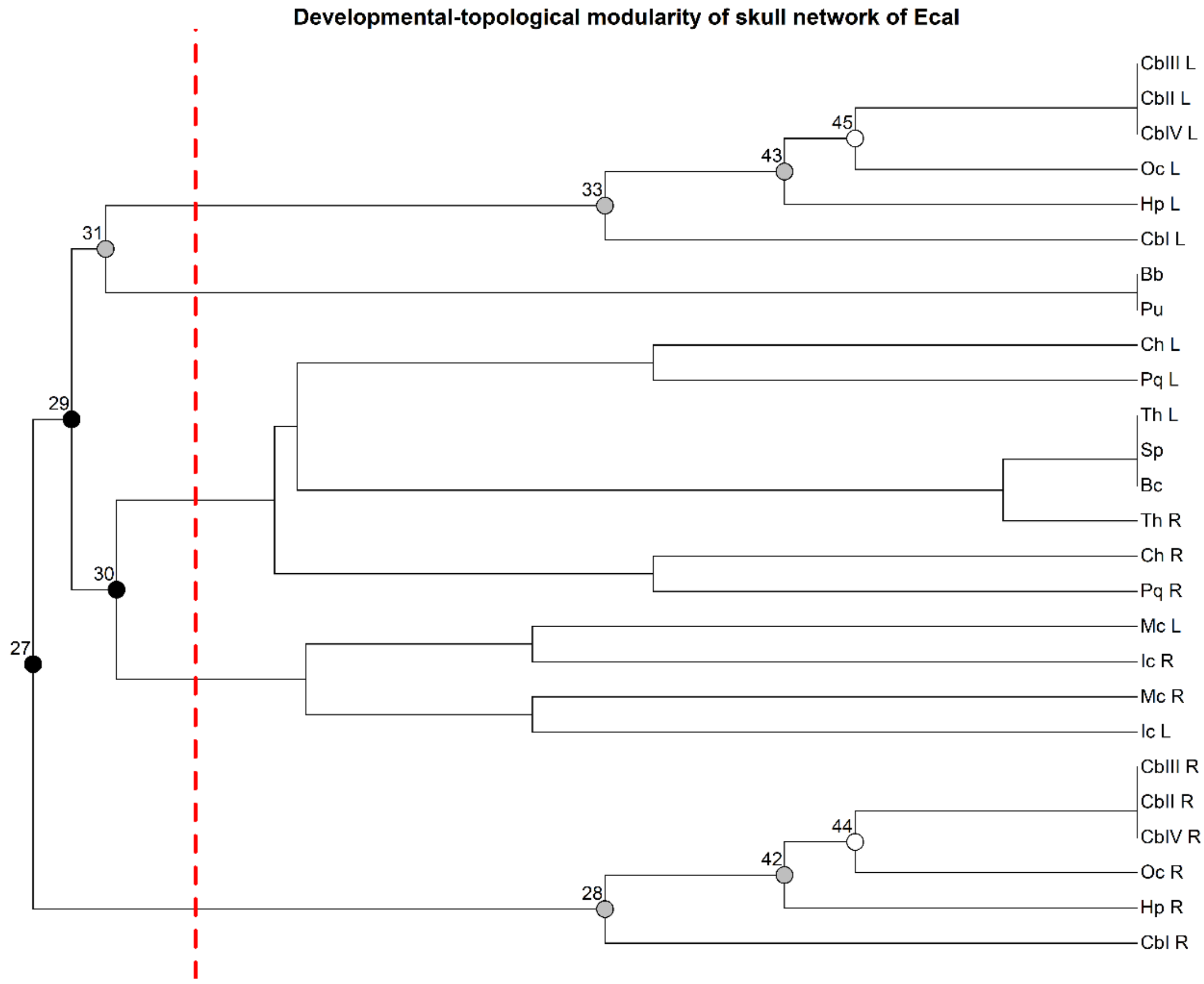
Developmental-topological modularity of skull network of Ecal
CbIII L
CbII L
CbIV L
Oc L
Hp L
CbI L
Bb
Pu
Ch L
Pq L
Th L
Sp
Bc
Th R
Ch R
Pq R
Mc L
Ic R
Mc R
Ic L
CbIII R
CbII R
CbIV R
Oc R
Hp R
CbI R
45
43
33
31
29
30
27
44
42
28

Developmental-topological modularity of skull network of Eeml

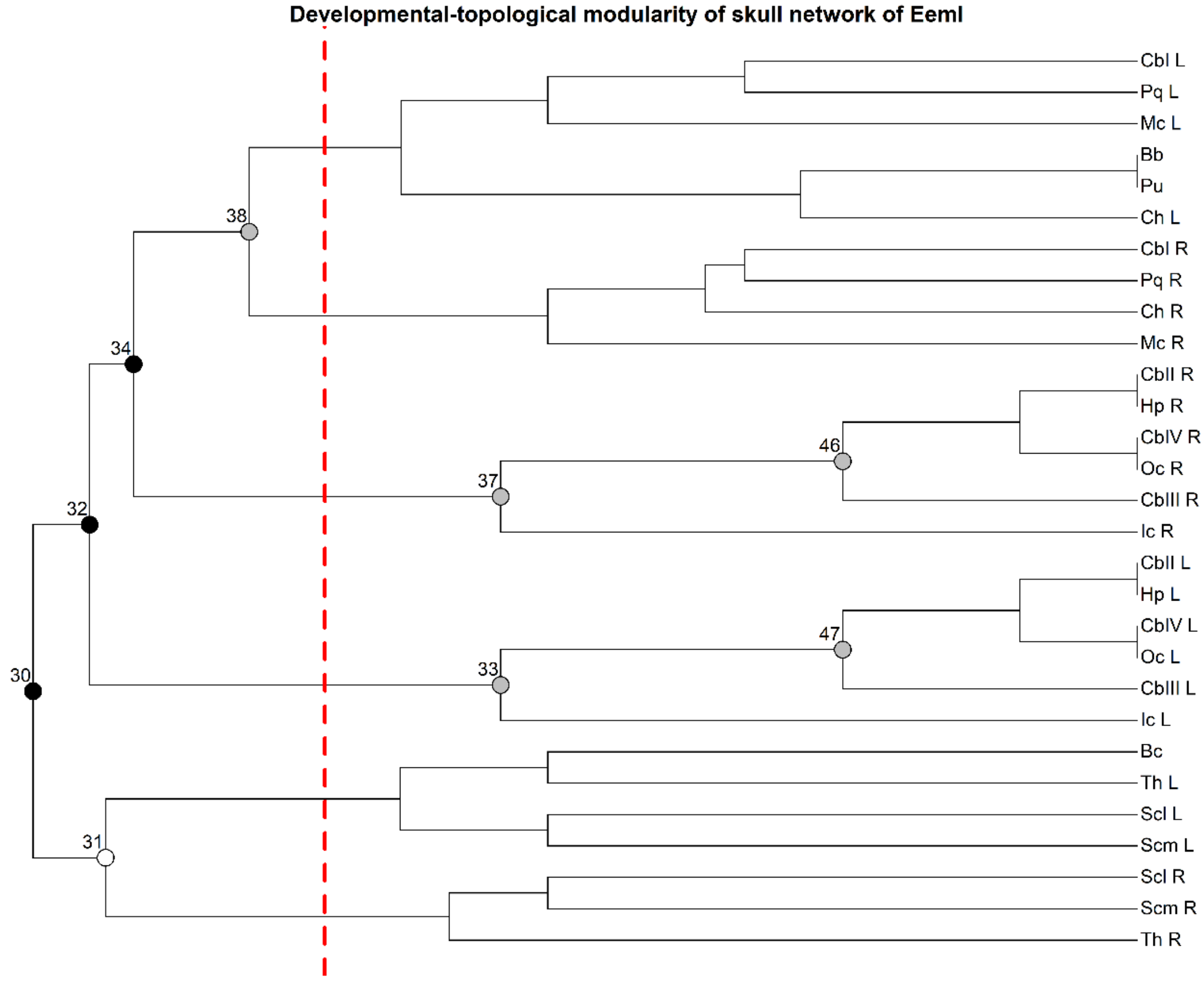

Developmental-topological modularity of skull network of Hboa

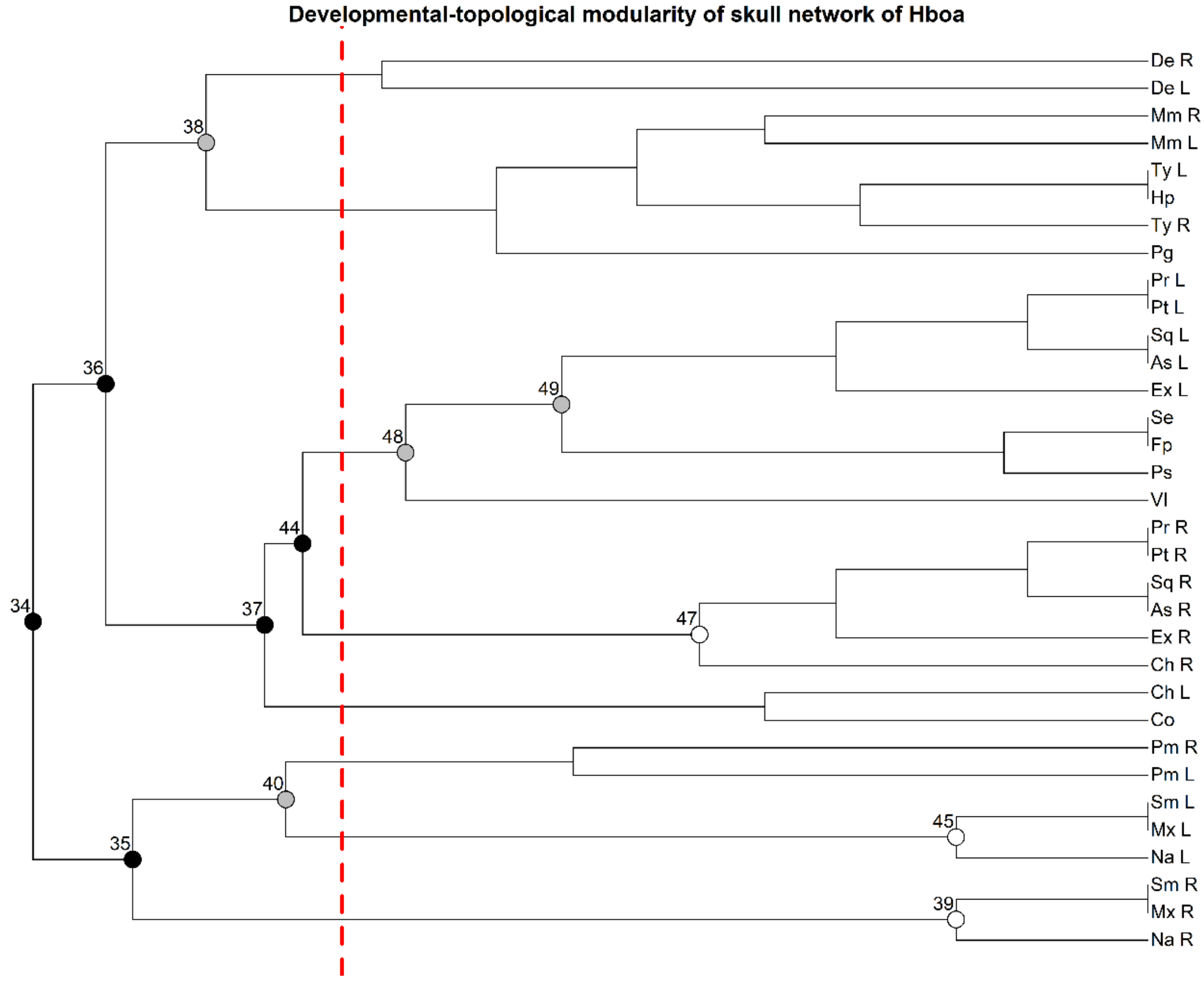

Developmental-topological modularity of skull network of Hbol

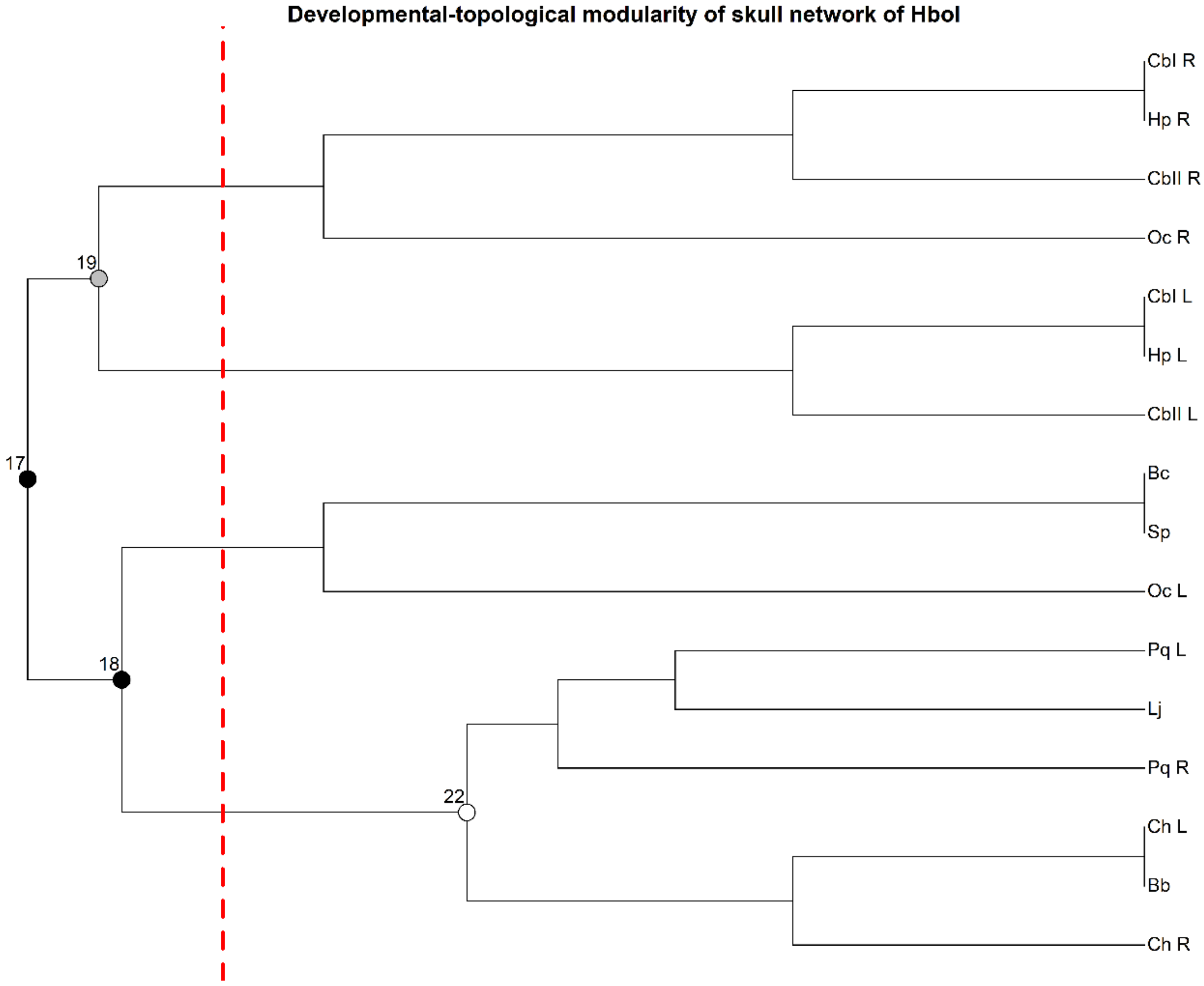

Developmental-topological modularity of skull network of Spha

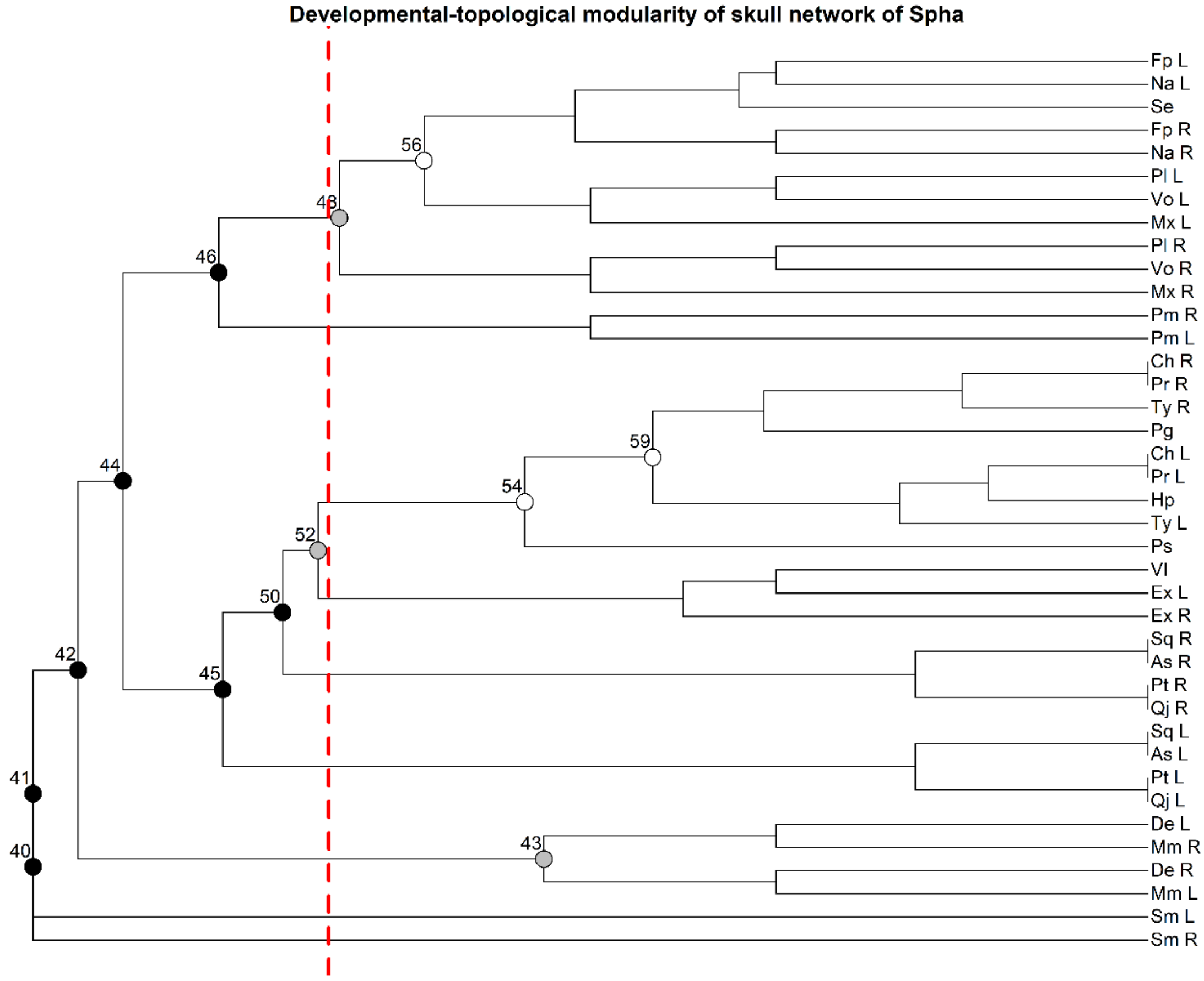

Developmental-topological modularity of skull network of SphI

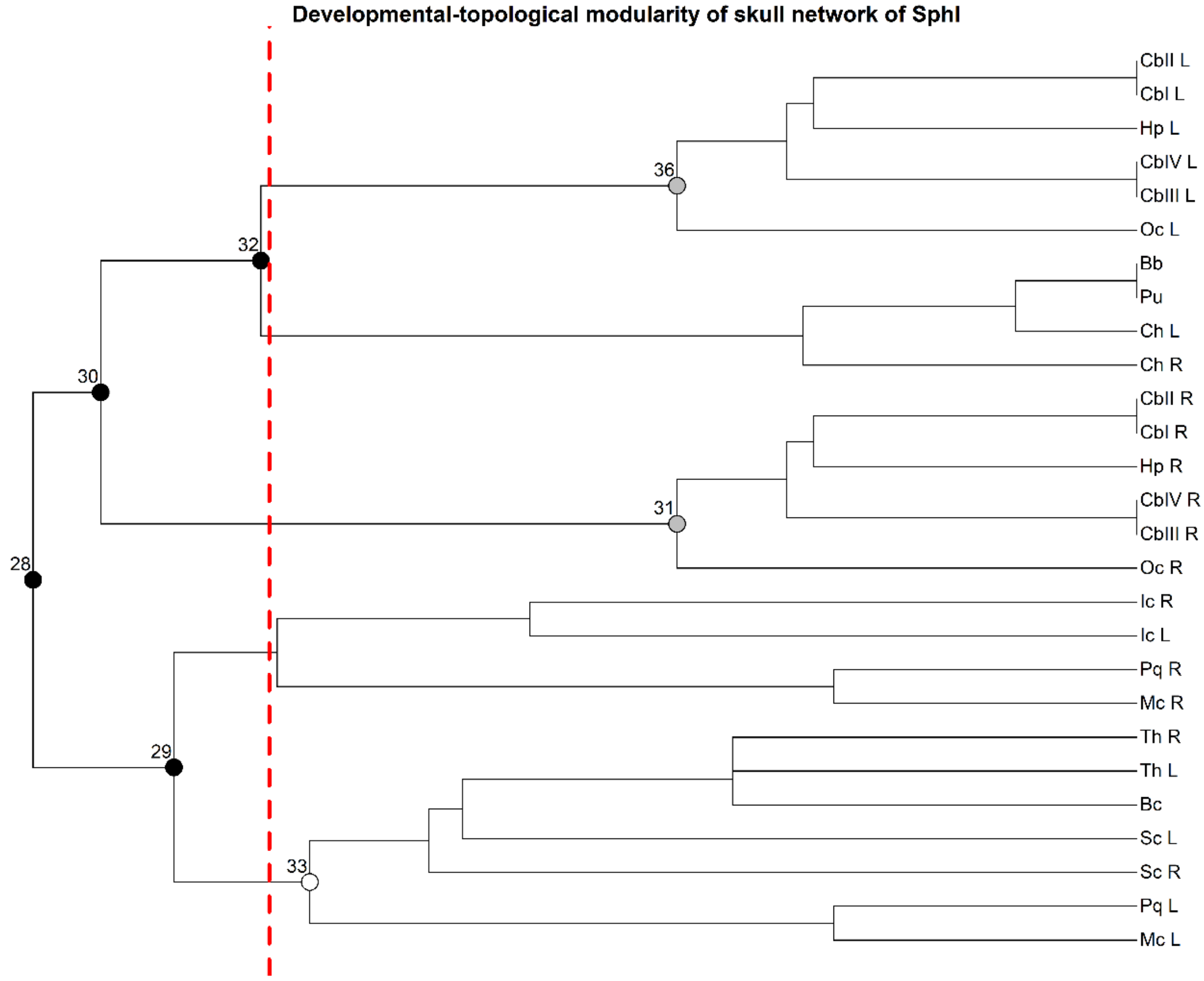

Developmental-topological modularity of skull network of Xlaa

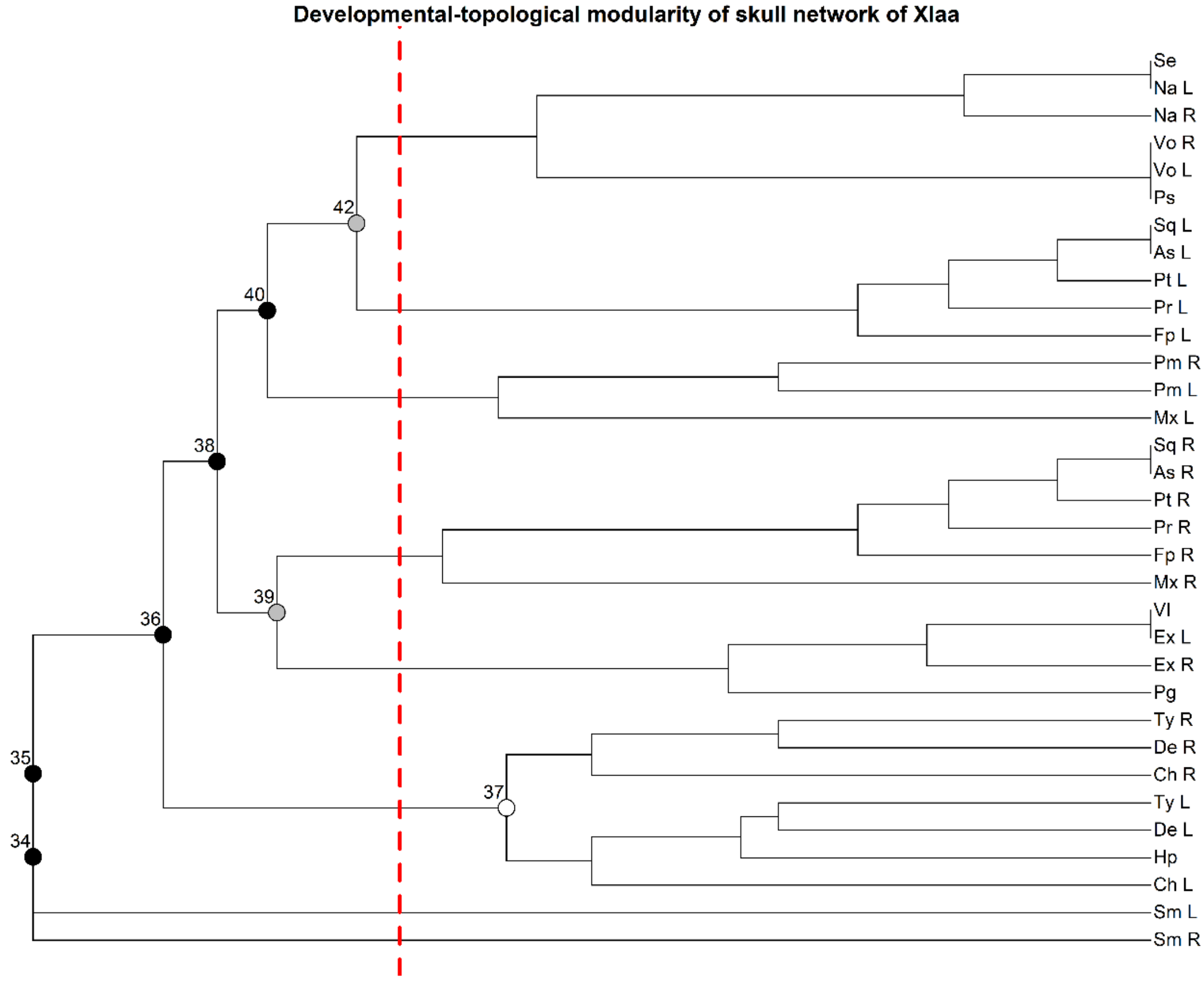

Developmental-topological modularity of skull network of Xlal

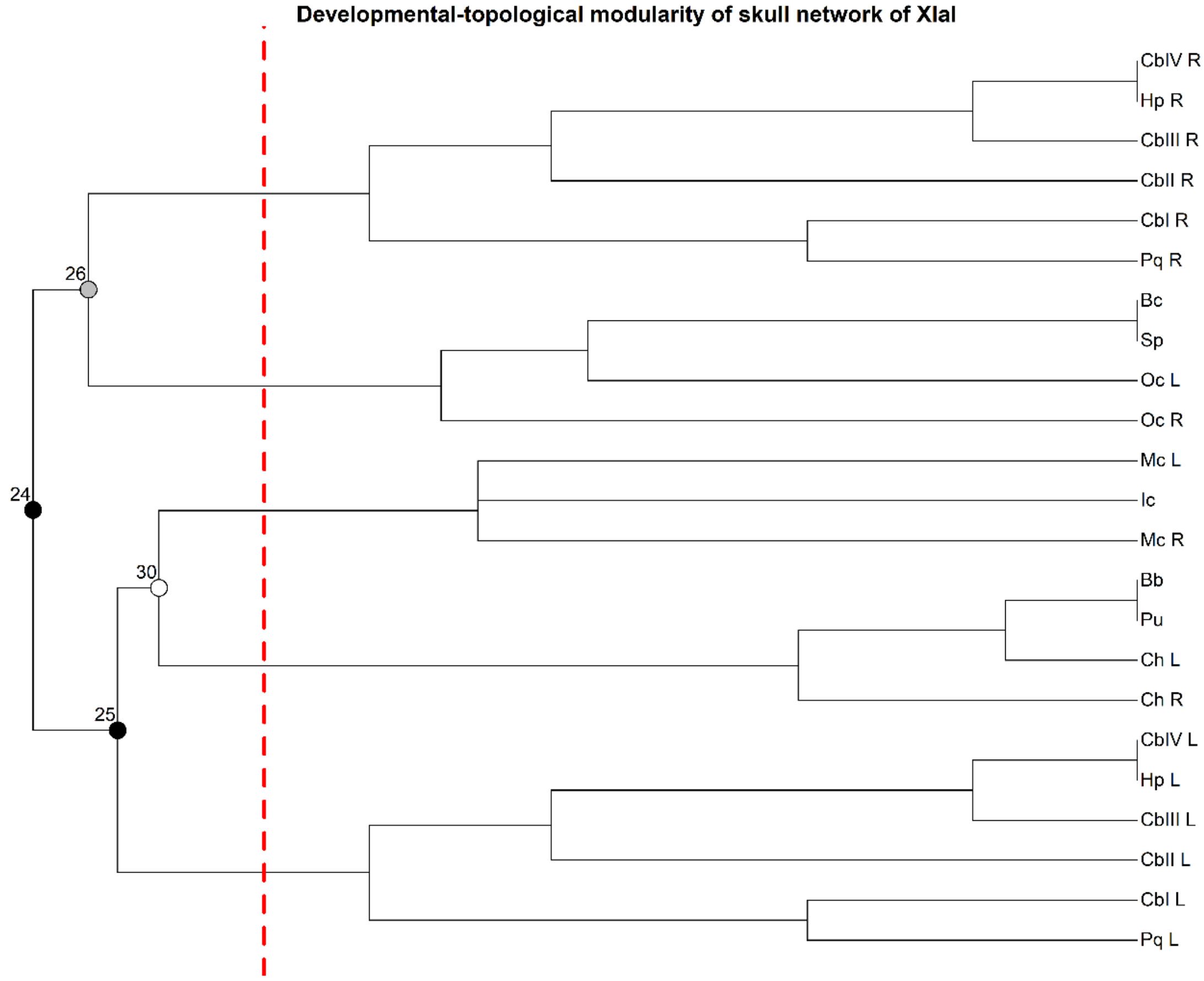

Figure S1.3 Principal Component Analysis of network parameters including N and K.

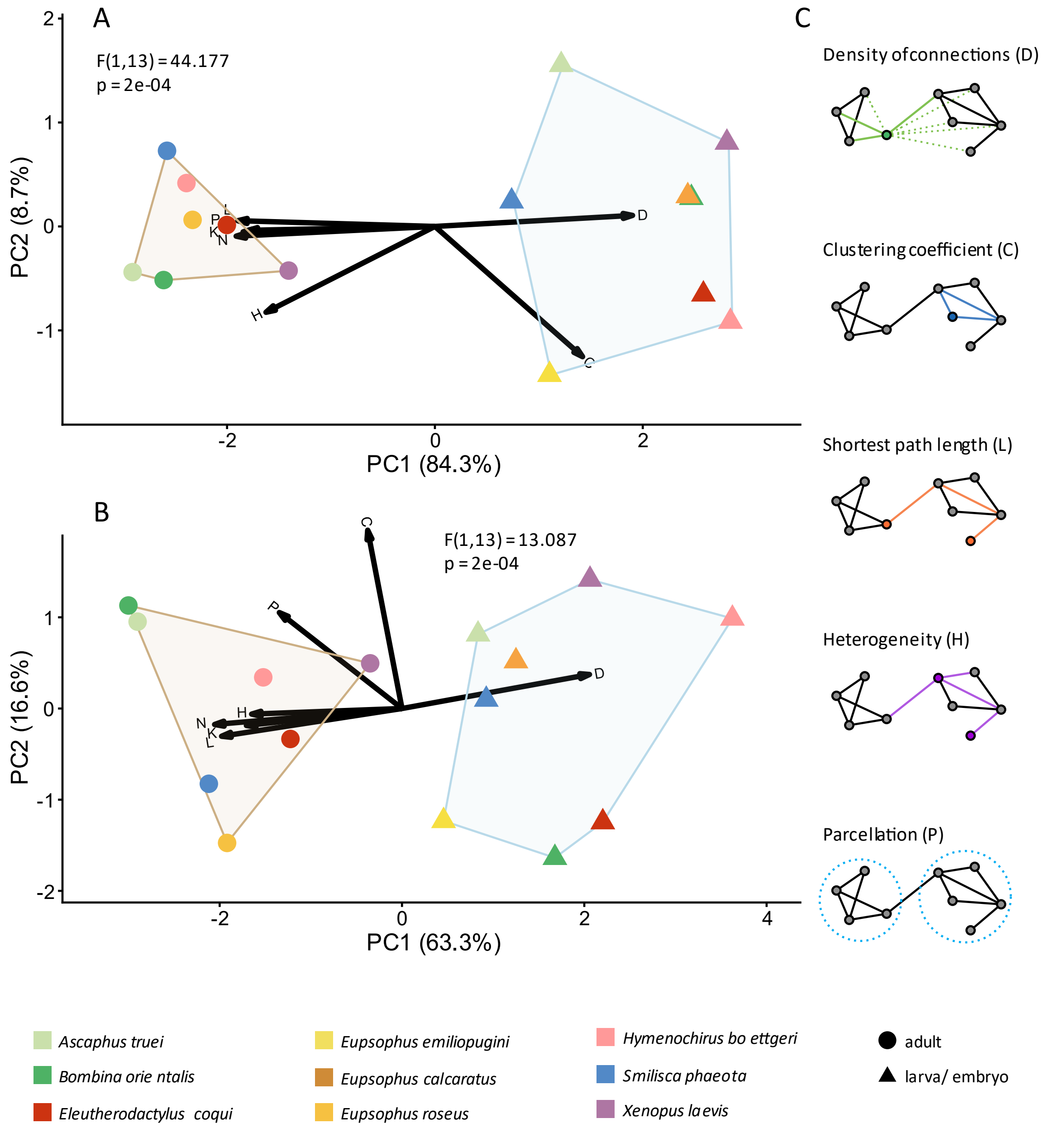

Figure S1.4 Anatomical networks and Q-modules of *Smilisca phaeota*.

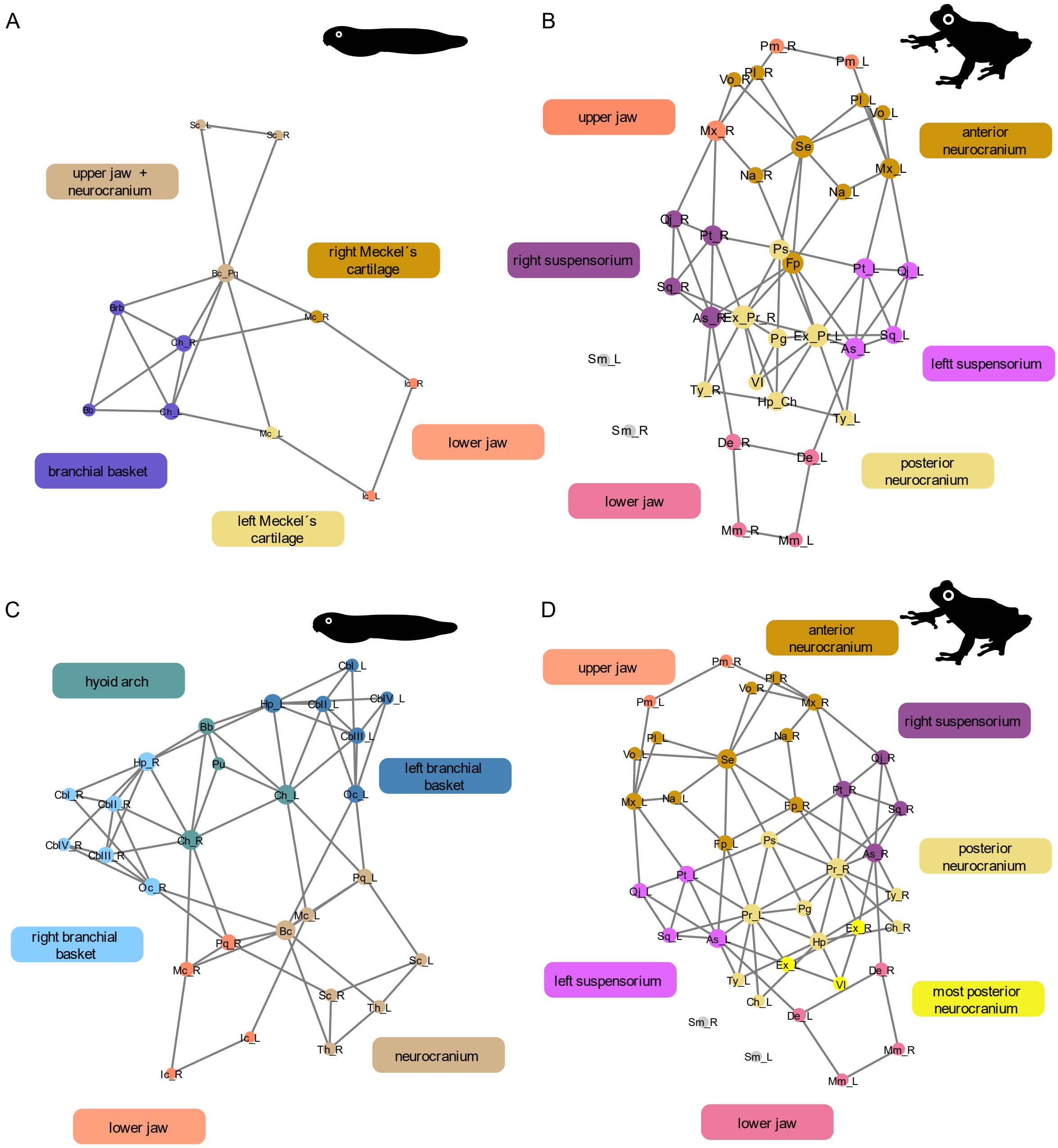

Figure S1.5 Anatomical networks and Q-modules of *Ascaphus truei* und *Bombina orientalis*.

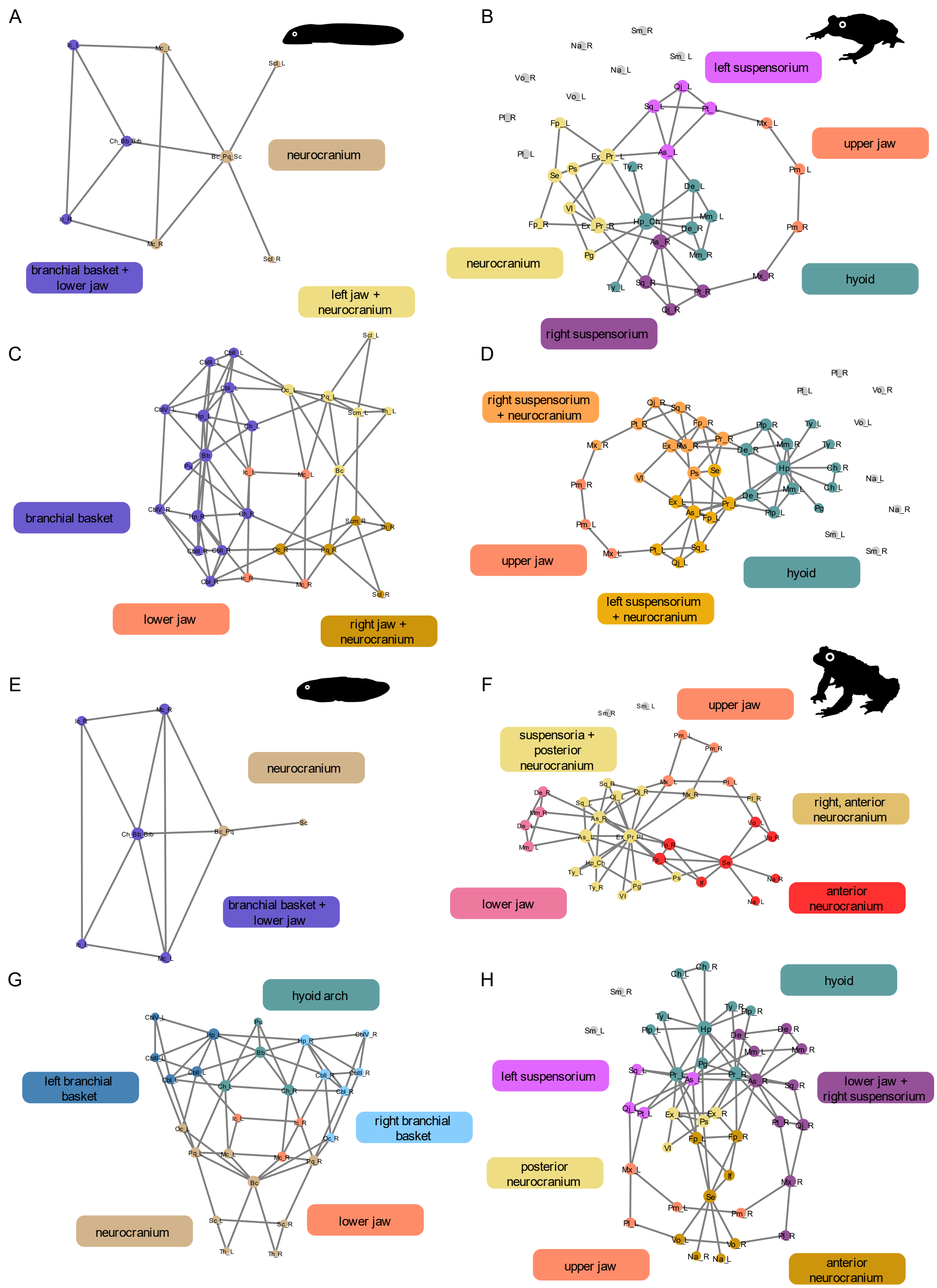

Figure S1.6 Anatomical networks and Q-modules of *Hymenochirus boettgeri* and *Xenopus laevis*.

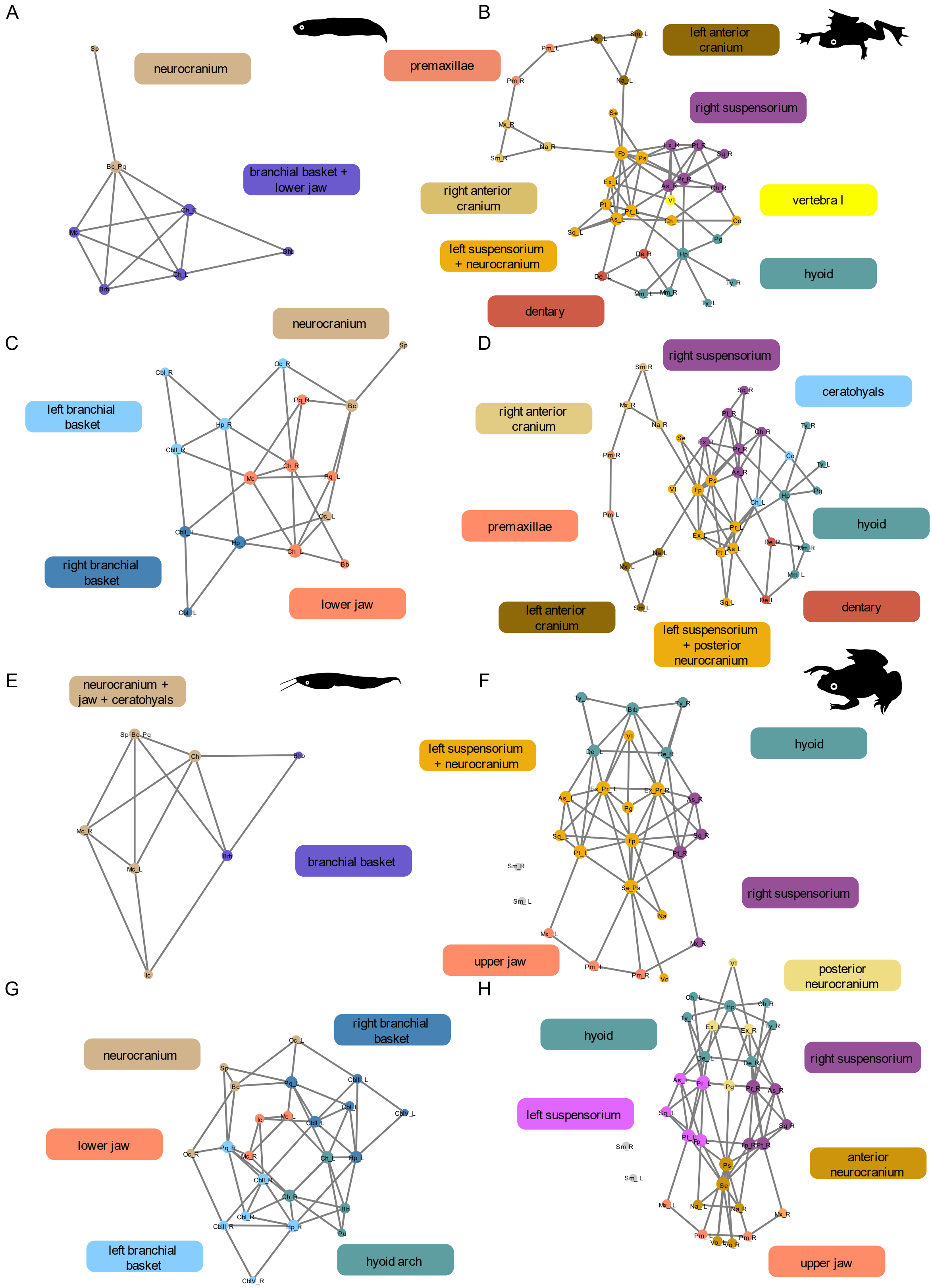

Figure S1.7 Anatomical networks and Q-modules of *Eleutherodactylus coqui* and *Eupsophus roseus*, *E. calcaratus* and *E. emiliopugini*.

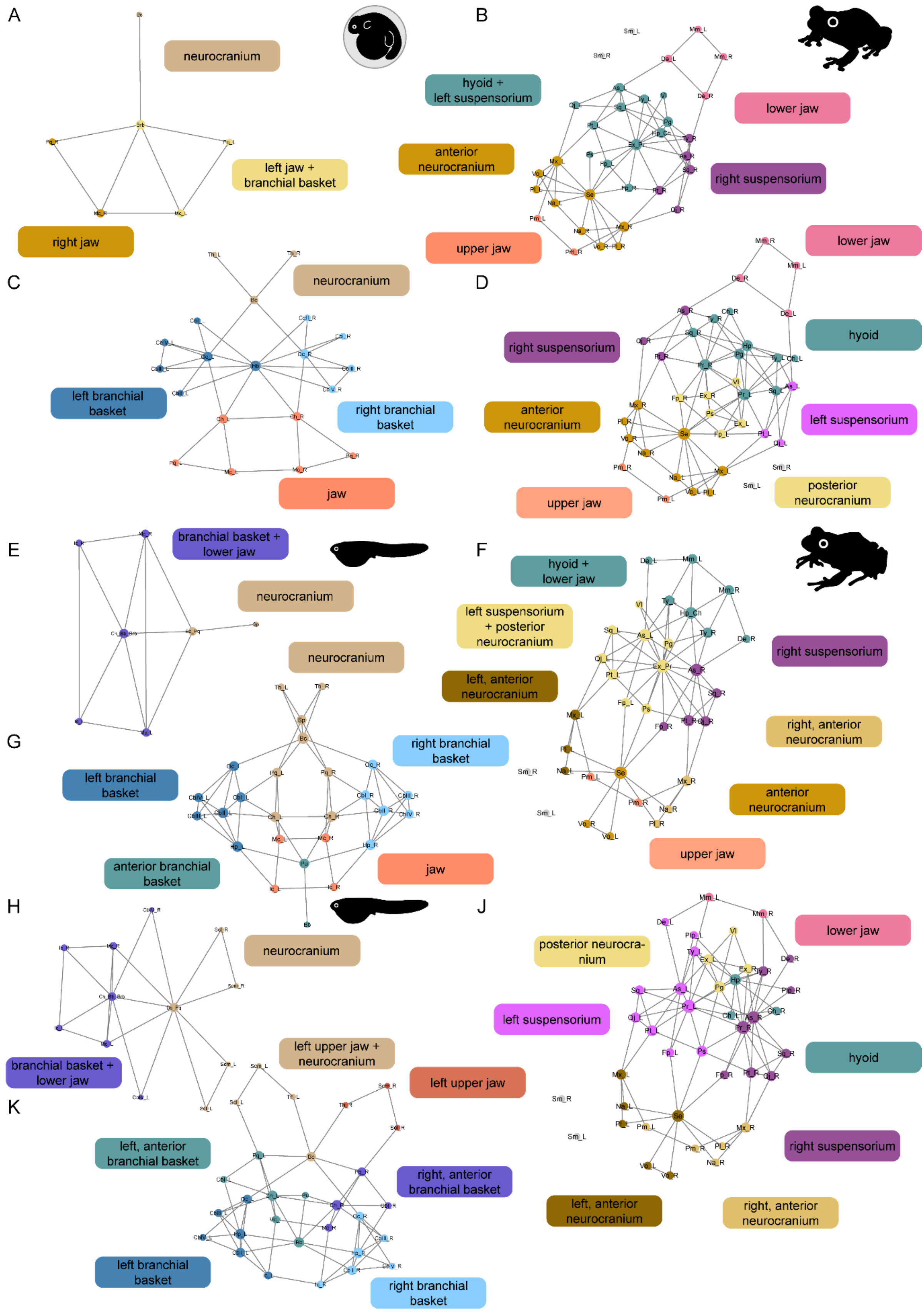

Figure S1.8 Current phylogeny of anurans following Liedtke et al. (2022).

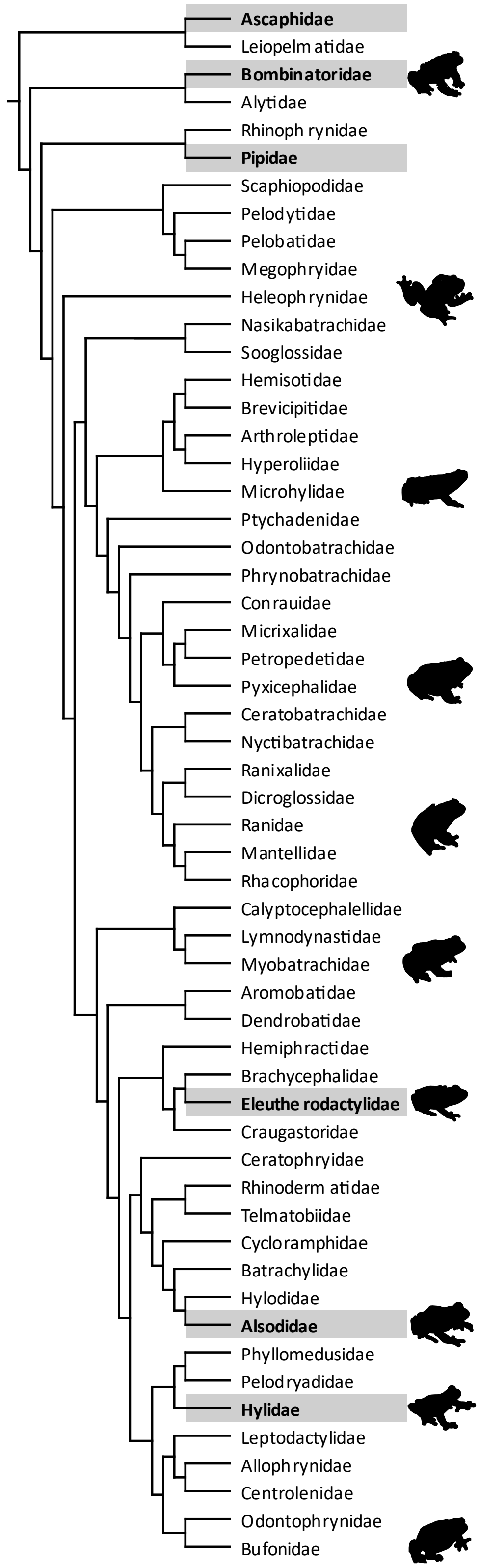

Supplement: Supplementary file 1 [file JMOR-287-e70185-s003.pdf]
