# Supplementary material for: Anatomical Network Analyses of Pre‐ and Post‐Metamorphic Cranial Systems Support the Adaptive Decoupling Hypothesis in Anura
Source: J Morphol. 2026 Oct 29;287(11):e70185. doi: 10.1002/jmor.70185 (PMC13717468; doi:10.1002/jmor.70185)

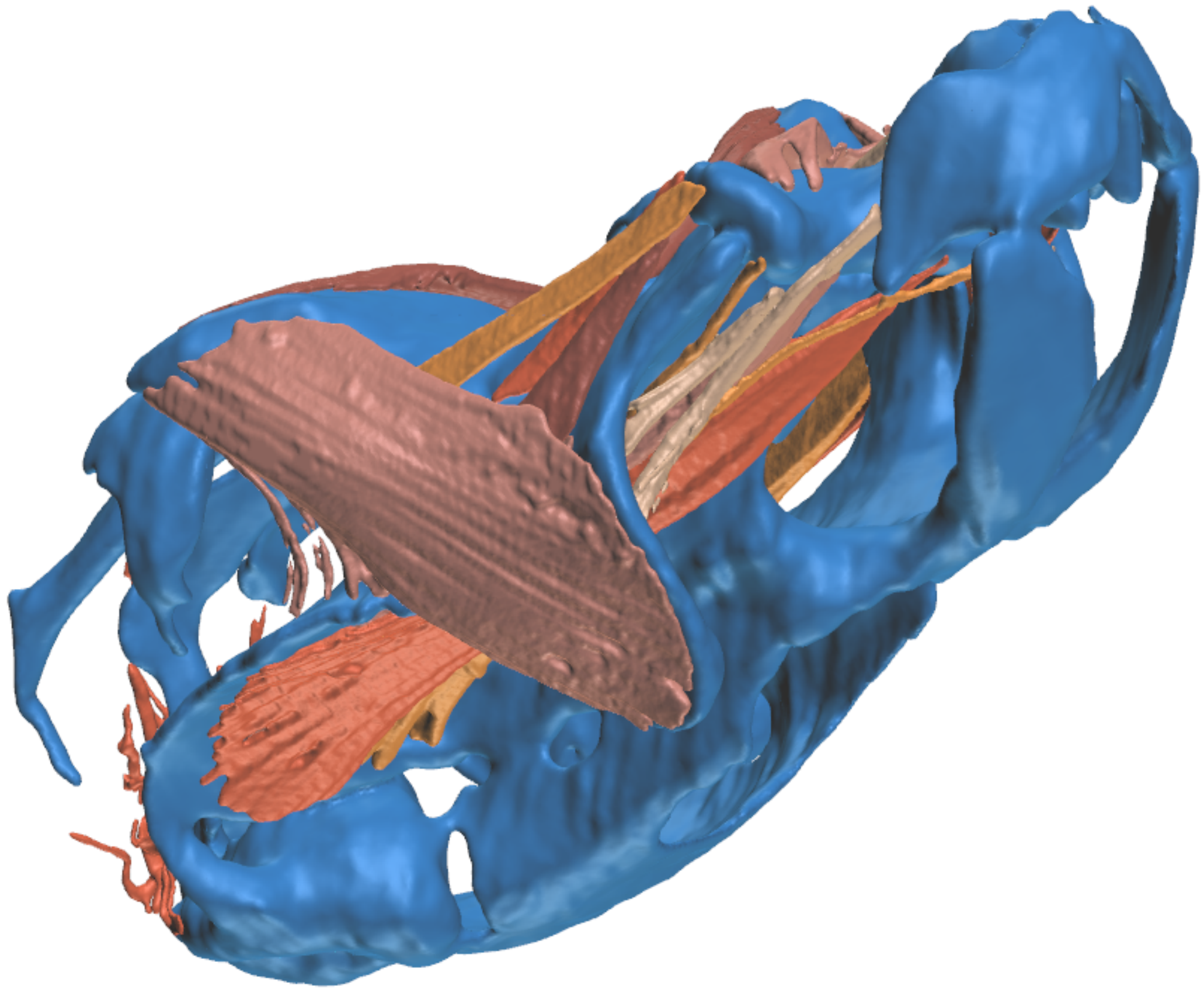

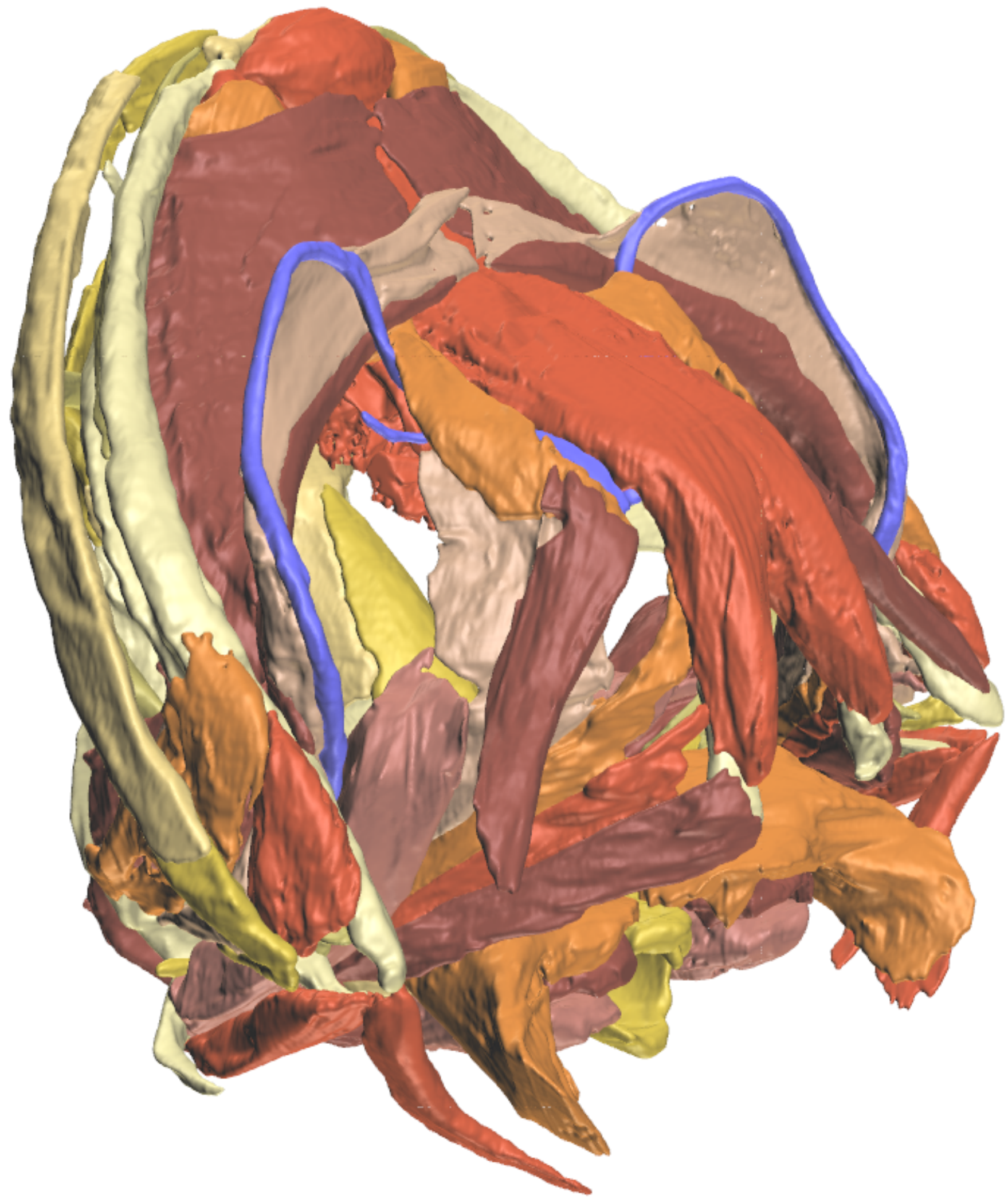

Supplement: Supplementary file 2 [file JMOR-287-e70185-s004.pdf]
