# Supplementary material for: Anatomical Network Analyses of Pre‐ and Post‐Metamorphic Cranial Systems Support the Adaptive Decoupling Hypothesis in Anura
Source: J Morphol. 2026 Oct 29;287(11):e70185. doi: 10.1002/jmor.70185 (PMC13717468; doi:10.1002/jmor.70185)
Supplement: Supplementary file 3 [file JMOR-287-e70185-s001.docx]

\# R-Script for "Anatomical networks of the cranial system of the cranial system support the Adaptive Decoupling Hypothesis in Anura"

\# Guschker, Lorenz; Stüwe, Maximilian A.; Müller, Hendrik; Naumann, Benjamin

\# Version 4.5.2

\# 16.06.2026

\# run through the code twice, once for topological analysis and once for developmental-topological analysis

\### loading in the packages ###

library(igraph)	# network analysis and visualization

library(ggplot2)	# data visualizations

library(writexl)	# export data frames to excel files

library(GGally)	# extension to 'ggplot2'

library(network)	# classes for relational data

library(sna)	# tools for social network analysis

library(intergraph)	# coercion routines for network data objects

library(RColorBrewer) # ColorBrewer palettes

library(readxl)	# read excel files

library(ape)	# analyses of phylogenetics and evolution

library(phytools) # phylogenetic tools for comparative biology (and other things)

library(vegan)	# community ecology package

library(tools)	# tools for package development

library(ggfortify)	# data visualization tools for statistical analysis results

library(ggpubr)	# customizing ggplots

library(tidyverse)	# transform data

\### custom functions ###

\# statistical test of modules, originally nonfunctional from Werneburg et al. 2019, modified and patched in Rawson et al. 2022 by defining the functions separately

community.significance.test <- function(graph, vids, ...) {

subgraph <- induced_subgraph(graph, vids)

in.degrees <- igraph::degree(subgraph)

out.degrees <- igraph::degree(graph, vids) - in.degrees

w.test <- wilcox.test(in.degrees, out.degrees, alternative="greater")

return(w.test)

}

#function used from "Cranial Integration and Disparity Among Bones Discriminate Between Primates and Non-Primate Mammals", from Esteve-Altava 2021

heterogeneity<-function(graph){

deg<-igraph::degree(graph)

res<-sd(deg, na.rm=TRUE)/mean(deg, na.rm=FALSE)

return(res)

}

\# function: GTOM (from http://labs.genetics.ucla.edu/horvath/GTOM/old/gtom.R), extracted from Werneburg et al. 2019

GTOMmdist1 = function(adjmat1,m=1){

if (m!=round(abs(m))){

stop("m must be a positive integer", call.=TRUE);}

if (any(adjmat1!=0 & adjmat1!=1)){

stop("The adjacency matrix must be binary", call.=TRUE);}

B <- adjmat1;

if (m>=2) {

for (i in 2:m) {

diag(B) <- diag(B) + 1;

B = B %*% adjmat1;}} # number of paths with length at most m connecting each pair

B <- (B>0); # m‐step reachability matrix

diag(B) <- 0; # exclude each node being its own neighbor

B <- B %*% B; # number of common k‐step neighbors that each pair of nodes share

Nk <- diag(B); # number of common k‐step neighbors that each node possesses

B <- B +adjmat1;

diag(B) <- 1;

denomTOM=outer(Nk,Nk,FUN="pmin")+1-adjmat1;

diag(denomTOM) <- 1;

1 - B/denomTOM	# turn the GTOM matrix into a dissimilarity

}

#function to prepare adjacency matrices, from Werneburg et al. 2019

na.zero <- function (x) {

x[is.na(x)] <- 0

return(x)

}

\# function to calculate parcellation index, from Rawson et al. 2022

parcellation_index <- function(membership){

Nm <- vector()

for (i in 1:max(membership)){

Nm[i] <- length(which(membership==i))

}

res <- 1-sum((Nm/length(membership))^2)

return(res)

}

\# pairwise PERMANOVA for statistical test of groups used in PCA, from Rawson et al. 2022

pairwise.adonis <- function(x, factors, sim.method = 'euclidean', p.adjust.m ='fdr')

{

library(vegan)

co = combn(unique(factors),2)

pairs = c()

F.Model =c()

R2 = c()

p.value = c()

DF1 = c()

DF2 = c()

for(elem in 1:ncol(co)){

ad = adonis(x[factors %in% c(co[1,elem],co[2,elem]),] ~ factors[factors %in% c(co[1,elem],co[2,elem])] , method =sim.method, permutations = 10000);

pairs = c(pairs,paste(co[1,elem],'vs',co[2,elem]));

F.Model =c(F.Model,ad$aov.tab[1,4]);

DF1 =c(DF1,ad$aov.tab[1,1]);

DF2 =c(DF2,ad$aov.tab[2,1]);

R2 = c(R2,ad$aov.tab[1,5]);

p.value = c(p.value,ad$aov.tab[1,6])

}

p.adjusted = p.adjust(p.value,method=p.adjust.m)

pairw.res = data.frame(pairs,DF1,DF2,F.Model,R2,p.value,p.adjusted)

return(pairw.res)

}

\### loading in data ###

\# loaded will be one Excel file containing the adjacency matrices

\# topological and developmental-topological analyses have separate excel files

\# every sheet will contain one individual species and life stage

\# first column contains mirrored description of the node

file <- ".xlsx"	# name of your file

sheets <- excel_sheets(file)	#get the names of all sheets

\# read each sheet and convert it to matrix

matrix <- lapply(sheets, function(sheet) {

df <- read_excel(file, sheet = sheet)

df <- as.data.frame(df)

rownames(df) <- df[[1]]

df[[1]] <- NULL

as.matrix(df)

})

names(matrix) <- sheets

matrices <- list(matrix$Ecoa, matrix$Ecol, matrix$Spha, matrix$Sphl, matrix$Xlaa, matrix$Xlal,

matrix$Bora, matrix$Borl, matrix$Atra, matrix$Atrl, matrix$Hboa, matrix$Hbol,

matrix$Eroa, matrix$Eeml, matrix$Ecal)

#creates names for all networks

names(matrices) <- c("gecoa", "gecol", "gspha", "gsphl", "gxlaa", "gxlal", "gbora", "gborl",

"gatra", "gatrl", "ghboa", "ghbol", "geroa", "geeml", "gecal")

graphs <- lapply(matrices, graph_from_adjacency_matrix, mode = "max") # convert each matrix into an igraph object

list2env(graphs, envir = .GlobalEnv) # export each graph into global environment

\##### Calculating and Compiling the rest of the network parameters #####

\### GTOM Analysis ###

\# Analysis is adapted from "Unique skull network complexity of Tyrannosaurus rex among land vertebrates", Werneburg et al. 2019

\# Loading the Data for the GTOM Analysis for adult and larval Anuran species (Eleutherodactylus, Smilisca, Xenopus, Bombina, Ascaphus, Hymenochirus, Eupsophus)

\# The file loaded is the same as before, but the first column of all adjacency matrix (name of skeletal element) is cut, as not necessary for following analysis

species <- c("Ecoa","Ecol","Spha","Sphl", "Xlaa","Xlal","Bora","Borl","Atra", "Atrl","Hboa","Hbol","Eroa","Ecal","Eeml") #Listing the names of the Sheets

tab_names <- excel_sheets(path = ".xlsx")#specifies the data path of the file. if you set the working directory to the source file location and have the file in there you will just need to write the file name there

mat.list <-	lapply(tab_names, function(x) {

df <- read_excel(path = ".xlsx", sheet = x, col_names = T) # "sheet = x" is the command to load in all the sheets

df[, -1] #cuts first column (name of skeletal elements)

})

adj.list <-	lapply(mat.list, data.matrix, rownames.force=TRUE)

pre.graph.list <- lapply(adj.list, graph_from_adjacency_matrix, mode="undirected")

graph.list <-	pre.graph.list

names(graph.list) <-	species

\## Community structure (modularity) ##

\# calculate GTOM and hierarchical clustering

adj.list <- lapply(adj.list, na.zero)

tom.list <- lapply(adj.list, GTOMmdist1, m=1)

dist.tom.list <- lapply(tom.list, as.dist)

hclust.list <- lapply(dist.tom.list, hclust, method="average")

for (i in 1:length(species)){hclust.list[[i]]$labels <- V(graph.list[[i]])$name}

phylo.list <- lapply(hclust.list, as.phylo)

\# function: jackknife of modularity Q value

jackknife_Q = function(graph, membership){

Qi <- vector()

for (j in 1:ecount(graph)){

g <- delete_edges(graph, j)

Qi[j] <- modularity(g, membership)

}

ss <- sum((Qi-mean(Qi))^2)

n <- (ecount(graph)-1)/ecount(graph)

Q.error <- sqrt(n*ss)

return(Q.error)

}

\# best partition based on Q max

best.partition <- vector()

for (i in 1:length(species)){

Qvalue <- vector()

for (j in 1:vcount(graph.list[[i]])){

Qvalue[j] <- modularity(graph.list[[i]], cutree(hclust.list[[i]], k=j))

}

best.partition[i] <- which(Qvalue==max(Qvalue))

}

\# partition based on statistical significance

Ho_modules_pvalue <- list()

for (i in 1:length(species)){

graph <- graph.list[[i]]

tree <- phylo.list[[i]]

tipN <- vcount(graph)

intN <- tipN-1

Ho_modules_pvalue_v <- vector()

for (j in 1:intN){

Ho_m <- extract.clade(tree, node=(tipN+j)) # get all subtrees

is_tip <- Ho_m$edge[,2] <= length(Ho_m$tip.label) #somehow currently an error in here!

ordered_tips <- Ho_m$edge[is_tip, 2]

who <- Ho_m$tip.label[ordered_tips]

index <- which((V(graph)$name %in% who)==TRUE)

Ho_mp <- community.significance.test(graph, vids=index)

Ho_modules_pvalue_v[j] <- Ho_mp$p.value

}

Ho_modules_pvalue[[i]] <- Ho_modules_pvalue_v

}

print(Ho_modules_pvalue)

#creating the dendrogram plots

for (i in 1:length(species)) {

#Defining the TIFF file name

file_name <- paste0("Topological_dendrogram_of_", species[i], ".tiff") #change name for developmental-topology analysis

tiff(file_name, width = 30, height = 24, units = "cm", res = 600)

#Plotting the tree

plotTree(phylo.list[[i]], lwd=1, mar=c(4.1,1.1,1.1,1.1))

#p‐value groups

sign <- which(Ho_modules_pvalue[[i]] < 0.05)

bs <- Ho_modules_pvalue[[i]][sign]

co <- c("black", "grey", "white")

p <- character(length(sign))

p[bs < 0.001] <- co[1]

p[bs >= 0.001 & bs < 0.01] <- co[2]

p[bs >= 0.01 & bs < 0.05] <- co[3]

nodelabels(node = (sign + vcount(graph.list[[i]])), pch = 21, bg = p, cex = 2)

nodelabels(phylo.list[[i]]$node.label, node = (sign + vcount(graph.list[[i]])), adj = c(1.1, -0.7), frame = "none")

#Qmax groups

obj <- ltt(phylo.list[[i]], plot = FALSE)

k <- best.partition[i]

h <- mean(obj$times[c(which(obj$ltt == k), which(obj$ltt == (k + 1)))])

lines(rep(h, 2), par()$usr[3:4], col = "red", lty = "dashed", lwd = 3.5)

\# Adding title

title(main = paste("Topological modularity of skull network of", species[i]))

\# Closing TIFF device to save the plot

dev.off()

}

#grouping the elements by module

members.list <- list()

for (i in 1:length(species)){

n <- 2

p <- 0

end <- FALSE

while (end==FALSE){

members <- cutree(hclust.list[[i]], n)

for (j in 1:n){p[j] <- community.significance.test(graph.list[[i]], vids=(members==j))$p.value}

if (all(p<0.05)==TRUE){

n <- n+1

} else {

n <- n-1

p <- 0

members <- cutree(hclust.list[[i]], n)

for (j in 1:n){p[j] <- community.significance.test(graph.list[[i]], vids=(members==j))$p.value}

end <- TRUE

}

}

members.list[[i]] <- members

}

\# modules information

modules <- matrix(NA, nrow=length(species), ncol=3)

rownames(modules) <- species

colnames(modules) <- c("Q Modules", "Qmax", "Qmax error")

for (i in 1:length(species)){

membership <- cutree(hclust.list[[i]], k=best.partition[i])

modules[i,1] <- best.partition[i]

modules[i,2] <- modularity(graph.list[[i]], membership)

modules[i,3] <- jackknife_Q(graph.list[[i]], membership)

}

modules1 <- as.data.frame(modules)

modules1$ID <- species

write_xlsx(modules1, "GTOM_modules_.xlsx") #readout to excel

#gathering identity of each modules for easier identification and coloration

#extracts Q-module membership of one species

membership <- cutree(hclust.list[[i]], k = best.partition[i])

names(membership) <- V(graph.list[[i]])$name

#stores all memberships in a list

Qmax.members.list <- list()

for (i in 1:length(species)) {

membership <- cutree(hclust.list[[i]], k = best.partition[i])

names(membership) <- V(graph.list[[i]])$name

Qmax.members.list[[i]] <- membership

}

names(Qmax.members.list) <- species

#creates a data frame, that features every node of every species and its module affiliation

module_df <- bind_rows(

lapply(names(Qmax.members.list), function(sp) {

membership <- Qmax.members.list[[sp]]

data.frame(

Species = sp,

Node = names(membership),

Module = membership

)

})

)

write_xlsx(module_df, "Qmax_modules_per_node.xlsx") # readout to Excel

\### Network Parameters####

parameters <-	matrix(NA, nrow=length(species), ncol=8)

rownames(parameters) <-	species

colnames(parameters) <-	c("N", "K", "D", "C", "L", "A","H","P")

parameters[,1] <-	mapply(vcount, graph.list)

parameters[,2] <-	mapply(ecount, graph.list)

parameters[,3] <-	mapply(edge_density, graph.list)

parameters[,4] <- mapply(transitivity, graph.list, type="average", isolates="NaN")

parameters[,5] <-	mapply(mean_distance, graph.list, directed=FALSE)

parameters[,6] <-	mapply(assortativity_degree, graph.list, directed=FALSE)

\### FUNCTION 'heterogeneity': CALCULATES H

parameters[,7] <-	mapply(heterogeneity, graph.list)

\### FUNCTION 'parcellation_index': CALCULATES P

ite <- 1

communities <- list()

for (i in 1:length(species)){

saveSpin <- list()

saveQ <- vector()

set.seed(73)

for (n in 1:ite){

saveSpin[[n]] <- leading.eigenvector.community(graph.list[[i]])

saveQ[n] <- modularity(saveSpin[[n]])

}

index <- which.max(saveQ)

communities[[i]] <- saveSpin[[index]]

}

communities_skeletal <- communities

for (i in 1:length(species)){

parameters[i,8] <- parcellation_index(membership(communities[[i]]))

}

parameters_skeletal <- as.data.frame(parameters)

parameters_skeletal$ID <- species

write_xlsx(parameters_skeletal, path = "GTOM_parameters.xlsx") # readout to Excel

\### Plotting the Networks with the module data ###

\# this will be used to color the nodes in their respective module

#custom color palette

pal <- c( "1"="yellow","2" = "lightgoldenrod", "3"="darkgoldenrod3", "4"="darkgoldenrod2", "20" ="tan1", "21" ="#8f6808", "22" = "#e1bf6d", "5"="firebrick1", "6"="palevioletred2","8"="salmon1", "9"="mediumorchid1", "32" = "#9c47b2", "11"="cadetblue","12"="skyblue1","13"="slateblue","14"="tan","15"="steelblue","16"="coral3", "23" ="#c77162", "24" = "#e5927f", "25" = "#90c963", "26" = "#7ca759", "17"="gray90","18"="gray80","19"="gray60")

#file identity is derived from "Qmax_modules_per_node.xlsx", it describes the affiliation to a module and allows coloring based on identity

identity_all <- read.csv2("Identity_all.csv", header = TRUE, row.names = NULL)

identity <- lapply(identity_all, function(col) col[!is.na(col)]) #creates a list without NAs of the data frame

\# adult Eleutherodactylus

set.seed(30) # stops randomizing network output

#set.seed(XX) for developmental-topology analysis

ecoa <- ggnet2(gecoa, size ="degree", label = T, edge.size = 0.75 , node.color = identity$Ecoa)+

scale_size_discrete("",range=c(5, 10), breaks = seq(12,1,-1))+

scale_color_manual(values=pal)

print(ecoa)

\# readouts as tiffs and svgs

ggsave("network_Eco_adult_modules_colorized_28.tiff", width = 30, height = 30, units = "cm", dpi = 1200)

ggsave(file = "network_Eco_adult_modules_colorized_28.svg", plot = ecoa, width = 20, height = 20, units = "cm")

\# embryonic Eleutherodactylus

set.seed(6)

#set.seed(XX)

ecol <- ggnet2(gecol, size ="degree", label = T, edge.size = 0.75 , node.color = identity$Ecol)+

scale_size_discrete("",range=c(5, 10), breaks = seq(12,1,-1))+

scale_color_manual(values=pal)

print(ecol)

ggsave("network_Eco_embryo_modules_colorized_6.tiff", width = 30, height = 30, units = "cm", dpi = 1200)

ggsave(file = "network_Eco_embryo_modules_colorized_6.svg", plot = ejol, width = 20, height = 20, units = "cm")

\# adult Smilisca

set.seed(32)

#set.seed(XX)

spha <- ggnet2(gspha, size ="degree", label = T, edge.size = 0.75 , node.color = identity$Spha)+

scale_size_discrete("",range=c(5, 10), breaks = seq(12,1,-1))+

scale_color_manual(values=pal)

print(spha)

ggsave("network_Sph_adult_modules_colorized_32.tiff", width = 30, height = 30, units = "cm", dpi = 1200)

ggsave(file = "network_Sph_adult_modules_colorized_32.svg", plot = spha, width = 20, height = 20, units = "cm")

\# larval Smilisca

set.seed(10)

#set.seed(XX)

sphl <- ggnet2(gsphl, size ="degree", label = T, edge.size = 0.75 , node.color = identity$Sphl)+

scale_size_discrete("",range=c(5, 10), breaks = seq(12,1,-1))+

scale_color_manual(values=pal)

print(sphl)

ggsave("network_Sph_larva_modules_colorized_10.tiff", width = 30, height = 30, units = "cm", dpi = 1200)

ggsave(file = "network_Sph_larva_modules_colorized_10.svg", plot = sphl, width = 20, height = 20, units = "cm")

\# adult Xenopus

set.seed(21)

#set.seed(XX)

xlaa <- ggnet2(gxlaa, size ="degree", label = T, edge.size = 0.75 , node.color = identity$Xlaa)+

scale_size_discrete("",range=c(5, 10), breaks = seq(12,1,-1))+

scale_color_manual(values=pal)

print(xlaa)

ggsave("network_Xla_adult_modules_colorized_21.tiff", width = 30, height = 30, units = "cm", dpi = 1200)

ggsave(file = "network_Xla_adult_modules_colorized_21.svg", plot = xlaa, width = 20, height = 20, units = "cm")

\# larval Xenopus

set.seed(10)

#set.seed(XX)

xlal <- ggnet2(gxlal, size ="degree", label = T, edge.size = 0.75 , node.color = identity$Xlal)+

scale_size_discrete("",range=c(5, 10), breaks = seq(12,1,-1))+

scale_color_manual(values=pal)

print(xlal)

ggsave("network_Xla_larva_modules_colorized_10.tiff", width = 30, height = 30, units = "cm", dpi = 1200)

ggsave(file = "network_Xla_larva_modules_colorized_10.svg", plot = xlal, width = 20, height = 20, units = "cm")

\# adult Bombina

set.seed(50)

#set.seed(XX)

bora <- ggnet2(gbora, size ="degree", label = T, edge.size = 0.75 , node.color = identity$Bora)+

scale_size_discrete("",range=c(5, 10), breaks = seq(12,1,-1))+

scale_color_manual(values=pal)

print(bora)

ggsave("network_Bor_adult_modules_colorized_48.tiff", width = 30, height = 30, units = "cm", dpi = 1200)

ggsave(file = "network_Bor_adult_modules_colorized_48.svg", plot = bora, width = 20, height = 20, units = "cm")

\# larval Bombina

set.seed(10)

#set.seed(XX)

borl <- ggnet2(gborl, size ="degree", label = T, edge.size = 0.75 , node.color = identity$Borl)+

scale_size_discrete("",range=c(5, 10), breaks = seq(12,1,-1))+

scale_color_manual(values=pal)

print(borl)

ggsave("network_Bor_larva_modules_colorized_2.tiff", width = 30, height = 30, units = "cm", dpi = 1200)

ggsave(file = "network_Bor_larva_modules_colorized_2.svg", plot = borl, width = 20, height = 20, units = "cm")

\# adult Ascaphus

set.seed(15)

#set.seed(XX)

atra <- ggnet2(gatra, size ="degree", label = T, edge.size = 0.75 , node.color = identity$Atra)+

scale_size_discrete("",range=c(5, 10), breaks = seq(12,1,-1))+

scale_color_manual(values=pal)

print(atra)

ggsave("network_Atr_adult_modules_colorized_14.tiff", width = 30, height = 30, units = "cm", dpi = 1200)

ggsave(file = "network_Atr_adult_modules_colorized_14.svg", plot = atra, width = 20, height = 20, units = "cm")

\# larval Ascaphus

set.seed(10)

#set.seed(XX)

atrl <- ggnet2(gatrl, size ="degree", label = T, edge.size = 0.75 , node.color = identity$Atrl)+

scale_size_discrete("",range=c(5, 10), breaks = seq(12,1,-1))+

scale_color_manual(values=pal)

print(atrl)

ggsave("network_Atr_larva_modules_colorized_10.tiff", width = 30, height = 30, units = "cm", dpi = 1200)

ggsave(file = "network_Atr_larva_modules_colorized_10.svg", plot = atrl, width = 20, height = 20, units = "cm")

\# adult Hymenochirus

set.seed(30)

#set.seed(XX)

hboa <- ggnet2(ghboa, size ="degree", label = T, edge.size = 0.75 , node.color = identity$Hboa)+

scale_size_discrete("",range=c(5, 10), breaks = seq(12,1,-1))+

scale_color_manual(values=pal)

print(hboa)

ggsave("network_Hbo_adult_modules_colorized_29.tiff", width = 30, height = 30, units = "cm", dpi = 1200)

ggsave(file = "network_Hbo_adult_modules_colorized_29.svg", plot = hboa, width = 20, height = 20, units = "cm")

\# larval Hymenochirus

set.seed(15)

#set.seed(XX)

hbol <- ggnet2(ghbol, size ="degree", label = T, edge.size = 0.75 , node.color = identity$Hbol)+

scale_size_discrete("",range=c(5, 10), breaks = seq(12,1,-1))+

scale_color_manual(values=pal)

print(hbol)

ggsave("network_Hbo_larva_modules_colorized_13.tiff", width = 30, height = 30, units = "cm", dpi = 1200)

ggsave(file = "network_Hbo_larva_modules_colorized_13.svg", plot = hbol, width = 20, height = 20, units = "cm")

\# adult Eupsophus roseus

set.seed(20)

#set.seed(XX)

eroa <- ggnet2(geroa, size ="degree", label = T, edge.size = 0.75 , node.color = identity$Eroa)+

scale_size_discrete("",range=c(5, 10), breaks = seq(12,1,-1))+

scale_color_manual(values=pal)

print(eroa)

ggsave("network_Ero_adult_modules_colorized_19.tiff", width = 30, height = 30, units = "cm", dpi = 1200)

ggsave(file = "network_Ero_adult_modules_colorized_19.svg", plot = eroa, width = 20, height = 20, units = "cm")

\# larval Eupsophus emiliopugini

set.seed(13)

#set.seed(XX)

eeml <- ggnet2(geeml, size ="degree", label = T, edge.size = 0.75 , node.color = identity$Eeml)+

scale_size_discrete("",range=c(5, 10), breaks = seq(12,1,-1))+

scale_color_manual(values=pal)

print(eeml)

ggsave("network_Eem_larva_modules_colorized_13.tiff", width = 30, height = 30, units = "cm", dpi = 1200)

ggsave(file = "network_Eem_larva_modules_colorized_13.svg", plot = eeml, width = 20, height = 20, units = "cm")

\# larval Eupsophus calcaratus

set.seed(20)

#set.seed(XX)

ecal <- ggnet2(gecal, size ="degree", label = T, edge.size = 0.75 , node.color = identity$Ecal)+

scale_size_discrete("",range=c(5, 10), breaks = seq(12,1,-1))+

scale_color_manual(values=pal)

print(ecal)

ggsave("network_Eca_larva_modules_colorized_19.tiff", width = 30, height = 30, units = "cm", dpi = 1200)

ggsave(file = "network_Eca_larva_modules_colorized_19.svg", plot = ecal, width = 20, height = 20, units = "cm")

\### PCA of GTOM parameters ###

\# pairwise PERMANOVA and ggbiplot, from Rawson et al 2022

\# creates PCAs of previously extracted network parameters, a new column age specifies life phase

gtom <- read.csv2(".csv", header = TRUE, row.names = NULL) # your file, first column species, second column age (adult, larva), following the parameters

str(gtom) #check structure

{

gtom$ID <- as.factor(gtom$ID)

gtom$age <- as.factor(gtom$age)

#makes sure, the parameters are numeric for further analysis

cols_to_convert <- c("N", "K", "D", "C", "L", "A", "H", "P")

gtom[cols_to_convert] <- lapply(gtom[cols_to_convert], as.numeric)

}

decorana(gtom[,c(3,4,5,6,7,9,10)]) #checks dimension of parameters

\# PCA

pca.gtomwo <- prcomp(gtom [c(5,6,7,9,10)], center = TRUE, scale. = TRUE) # excludes parameters N,K and A

summary(pca.gtomwo)

\# Fine tuning of visualization of PCA

\# p1 describes PCA including all parameters of GTOM except A, N and K

\# adjust axis of PCA

minx<-min(pca.gtomwo$x[,1])-0.25

maxx<-max(pca.gtomwo$x[,1])+0.25

miny<-min(pca.gtomwo$x[,2])-0.25

maxy<-max(pca.gtomwo$x[,2])+0.25

p1<-ggbiplot::ggbiplot(pca.gtomwo, choices= 1:2, obs.scale=1,var.scale=1,ellipse=FALSE,alpha=0) +

#geom_text(aes(label=networks),size=3) +

scale_shape_discrete(name=NULL) +

stat_chull(aes(color = gtom$age , fill = gtom$age), alpha = 0.1, geom = "polygon", show.legend = FALSE) +

geom_point(aes(shape= gtom$age,color = gtom$ID), size = 5) +

theme_classic() +

theme(legend.direction='vertical',legend.position='right', axis.text=element_text(size=12),

axis.title=element_text(size=14)) +

coord_fixed(xlim=c(minx,maxx),ylim=c(miny,maxy)) +

scale_color_manual(values = c("Eco" = "#cc3311", "Ejo" = "#cc3311", "Bor" = "#4Eb265", "Atr" = "#CAE0AB",

"Sph" = "#5289C7", "Xla" = "#AE76A3", "Hbo" = "#FF9999", "Ero" = "#F6c141",

"Eem" = "#f6df41", "Eca" = "#f6a341",

"adult" = "#ccaf84", "larva" = "#b8d9e9"))+

scale_fill_manual(values =c("adult"="#ccaf84", "larva"="#b8d9e9"))

print(p1)

#saves the PCA plot

ggsave("PCA_of_GTOM_parameters.tiff", width = 20, height = 20, units = "cm", dpi = 1200)

ggsave(file = "PCA_of_GTOM_parameters.svg", plot = p2, width = 20, height = 20, units = "cm")

\# pairwise PERMANOVA comparing adults and larvae excluding N and K

permapcawo<-adonis2(pca.gtomwo$x~gtom$age,method='eu',permutations=10000) # same that using scale(parameters) ~ groups

print(permapcawo)

\## plotting this is not necessary, but visualizes the results of the PERMANOVA

test_text<-paste("F(",permapcawo$Df[1],",",permapcawo$Df[2],")"," = ",round(permapcawo$F[1],3),", p = ",signif(permapcawo$`Pr(>F)`[1],4),sep="")

p2<-ggbiplot:: ggbiplot(pca.gtomwo,obs.scale=1,var.scale=1,ellipse=TRUE,ellipse.prob=0.68,groups=gtom$age,alpha=0) +

scale_color_brewer(name='',type="div",palette="Set1") +

theme_classic() +

theme(legend.direction='horizontal',legend.position='top') +

geom_point(aes(shape=factor(gtom$age),colour=factor(gtom$age)),show.legend=TRUE) +

annotate("text",x= minx + 0.1 ,y= maxy - 0.1,label=test_text,hjust=0,size=4.5) + # CHECK BEST POSITIONS

coord_fixed(xlim=c(minx,maxx),ylim=c(miny,maxy))

print(p2)

ggsave("PCA_of_GTOM_parameters_pairwise_PERMANOVA.tiff", width = 20, height = 20, units = "cm", dpi = 1200)

\### Distance matrix of the GTOM parameters ###

data <- read.csv2(".csv", header = T, row.names = NULL) # your file, first column species, second age (adult/larva), following network parameters

rownames(data) <- data$ID #keeps ID in the analysis by naming the row

data = subset(data, select = -c(ID,N,K,A)) #cuts columns to studied network parameters

#Distance matrix of GTOM parameters C, D, L, H, P between all individuals

dm <- as.matrix(dist(data, method = "euclidean", diag = FALSE, upper = TRUE, p = 2))

fradm <- as.data.frame(dm)

write_xlsx(fradm,"Distance_matrix.xlsx") #readout of distance matrix

\# Define groups

adults <- c("Ecoa", "Spha", "Bora", "Atra", "Hboa", "Xlaa", "Eroa")

larvae <- c("Ecol", "Sphl", "Borl", "Atrl", "Hbol", "Xlal", "Ecal", "Eeml")

pair_map <- data.frame(

adult = c("Ecoa", "Spha", "Bora", "Atra", "Hboa", "Xlaa", "Eroa", "Eroa"),

larva = c("Ecol", "Sphl", "Borl", "Atrl", "Hbol", "Xlal", "Ecal", "Eeml")

)

\# Compute for each larva

resultsLL <- lapply(1:nrow(pair_map), function(i) {

L <- pair_map$larva[i]

A <- pair_map$adult[i]

\# Distance larva to respective adult

d_LA <- dm[L, A]

\# Mean distance larva to other larvae (excluding itself)

mean_LL <- mean(dm[L, larvae[larvae != L]])

data.frame(larva=L, adult=A, d_LA=d_LA, mean_LL=mean_LL, diff=d_LA - mean_LL)

})

resultsLL <- do.call(rbind, resultsLL)

print(resultsLL)

#test whether larval–adult distances were smaller than larval–larval distances using a Wilcoxon signed-rank test on the paired differences (d_LA – mean_LL), positive diff means closer to other larvae, negative closer to the respective adult

wilcox.test(resultsLL$diff, mu = 0, alternative = "greater", exact = FALSE)

#compute for each adult

resultsAA <- lapply(1:nrow(pair_map), function(i) {

L <- pair_map$larva[i]

A <- pair_map$adult[i]

\# Distance larva to respective adult

d_LA <- dm[L, A]

\# Mean distance larva to other larvae (excluding itself)

mean_AA <- mean(dm[A, adults[adults != A]])

data.frame(larva=L, adult=A, d_LA=d_LA, mean_AA=mean_AA, diff=d_LA - mean_AA)

})

resultsAA <- do.call(rbind, resultsAA)

print(resultsAA)

#test whether larval–adult distances were smaller than adult–adult distances using a Wilcoxon signed-rank test on the paired differences (d_LA – mean_LL), positive diff means closer to other larvae, negative closer to the respective adult

wilcox.test(resultsAA$diff, mu = 0, alternative = "greater", exact = FALSE)
